# Engineering Active Metal and Nonmetal Sites in Porous Structures of Metal‐Hydroxide Clusters for Enhanced D_2_/H_2_ Uptake and Separation

**DOI:** 10.1002/advs.202519498

**Published:** 2025-12-14

**Authors:** Zhuozhou Xie, Zhu Zhuo, Zi‐Ang Nan, Qing Li, Wenlin Wu, Yunzhe Zhou, Zi‐Xiu Lu, Jin Liu, Luyao Liu, Wenjing Wang, Daqiang Yuan, You‐Gui Huang

**Affiliations:** ^1^ State Key Laboratory of Structure Chemistry Fujian Institute of Research on the Structure of Matter Chinese Academy of Science Fuzhou Fujian 350002 China; ^2^ University of Chinese Academy of Sciences Beijing 100049 China; ^3^ Xiamen Key Laboratory of Rare Earth Photoelectric Functional Materials Xiamen Institute of Rare Earth Materials Haixi Institutes Chinese Academy of Sciences Xiamen Fujian 361021 China

**Keywords:** chemical affinity quantum sieving, cluster, D_2_/H_2_ separation, open metal site, supramolecular assembly framework

## Abstract

Efficient deuterium separation from hydrogen isotopic mixtures poses a significant challenge due to the nearly identical physicochemical properties of D_2_ and H_2_. Porous supramolecular assembly frameworks (SAFs) have emerged as promising candidates to address this issue, as their pore structures can be precisely tailored to achieve efficient hydrogen isotope separation. Herein, two metal‐hydroxide cluster‐based SAFs constructed from metal centers (Co^2+^ and Cu^2+^) coordinating with ligand L (HL = 1H‐benzo[f]isoindole‐1,3‐diimine) are reported. Solvothermal reaction of HL with Co(NO_3_)_2_·6H_2_O yielded [Co^II^_12_Co^III^_8_L_12_(*µ*_3_‐OH)_24_]·12(NO_3_) (**1**) which lacks active sites. In contrast, a similar reaction using Cu(ClO_4_)_2_·6H_2_O produced [Cu_20_L_8_(*µ*_3_‐OH)_24_(H_2_O)_8_]·2(C_2_H_6_NH_2_)·5(ClO_4_)·2Cl·3(CHO_2_) (**2**), featuring both active Cu^2+^ and = NH. Activated **2** exhibits the second‐highest D_2_ uptake of 301 cm^3^·g^‒1^ at 77 K and 100 kPa, significantly surpassing that of activated **1** (87 cm^3^·g^‒1^). Furthermore, activated **2** demonstrates exceptionally high D_2_/H_2_ separation efficiency, with a D_2_ retention time of 52 min·g^‒1^ for a D_2_/H_2_/Ne mixture (2.5/2.5/95 vol.%) at a flow rate of 8 mL·min^‒1^. Computational analyses indicate that the superior D_2_/H_2_ uptake and separation of activated **2** can be attributed to the synergistic effect of its active Cu^2+^ and = NH. These findings offer a novel strategy for incorporating multiple active adsorption sites into porous SAFs to optimize D_2_/H_2_ uptake and separation.

## Graphical abstract

Two supramolecular assembly frameworks, constructed from metal‐hydroxide clusters, are synthesized via coordination‐driven self‐assembly mediated by Co^2+^ and Cu^2+^ metal centers. One of them demonstrates exceptional D_2_ and H_2_ uptake capabilities, achieving the second‐highest uptake capacity for D_2_. Breakthrough experiments at 77 K confirm highly efficient D_2_/H_2_ separation, which is attributed to the synergistic effect of open Cu^2+^ sites and active = NH groups.

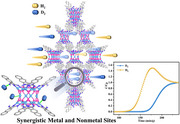

## 1 Introduction

Deuterium (D), a stable isotope of hydrogen, is essential across various scientific and industrial applications, particularly in neutron scattering,^[^ ^1^, ^2^, ^3^ ^]^ isotope tracing,^[^ ^4^, ^5^, ^6^ ^]^ and NMR spectroscopy.^[^ ^7^ ^]^ However, the separation of hydrogen isotopes remains a significant challenge due to their nearly identical physicochemical properties. Conventional methods such as cryogenic distillation and thermal diffusion are often hampered by high energy consumption,^[^ ^8^, ^9^, ^10^, ^11^, ^12^, ^13^ ^]^ which has spurred the search for alternative separation technologies.

Porous material‐based hydrogen isotope separation through kinetic quantum sieving (KQS)^[^ ^14^ ^]^ and chemical affinity quantum sieving (CAQS)^[^ ^15^ ^]^ has attracted considerable interest. For efficient KQS‐based separation, porous materials must feature ultrafine pore apertures.^[^ ^16^, ^17^ ^]^ In contrast, CAQS, first demonstrated in 2013, relies on differences in zero‐point energy (ZPE) to favor the adsorption of the heavier isotope at strong binding sites, providing an alternative mechanism for distinguishing D_2_ and H_2_.^[^ ^15^ ^]^ Unlike KQS, the CAQS effect allows effective hydrogen isotope separation at elevated temperatures (≥ 77 K). Current CAQS‐based materials for D_2_/H_2_ separation depend exclusively on either open metal sites (OMSs)^[^ ^18^, ^19^ ^]^ or nonmetal active centers.^[^ ^20^, ^21^ ^]^ For instance, several metal–organic frameworks (MOFs) with high OMS densities, such as MOF‐74^[^ ^18^ ^]^ and Cu(I)‐MFU‐4l,^[^ ^19^ ^]^ have achieved efficient D_2_/H_2_ separation. However, their susceptibility to moisture and air significantly limits their practical applicability and recyclability.^[^ ^22^, ^23^ ^]^ Alternatively, nonmetal active sites have also been shown to facilitate D_2_/H_2_ discrimination via CAQS.^[^ ^20^, ^21^ ^]^ Nevertheless, the integration of both metal and nonmetal sites within a single porous material to optimize D_2_/H_2_ separation remains largely unexplored,^[^ ^24^ ^]^ representing a highly promising strategy for developing advanced materials with superior D_2_/H_2_ uptake and separation performance.

Recently, we reported a supramolecular assembly framework (SAF) constructed from the metal‐hydroxide cluster [Co^II^_12_Co^III^_8_L″(µ_3_‐OH)_24_(ClO_4_)]·4Cl·7(HCO_2_) (**3**) with extremely narrow pores (∼4.6 Å),^[^ ^25^ ^]^ which shows moderate D_2_/H_2_ uptake and differentializes D_2_ and H_2_ via its small pore aperture. We hypothesized that employing 1H‐benzo[f]isoindole‒1,3‐diimine (HL) with a longer *π*‐conjugated system to replace HL″ as the ligand would yield larger‐pore SAFs. In particular, optimized D_2_/H_2_ uptake and separation may be achieved via CAQS if active adsorption sites can be incorporated into the resulting porous SAFs bearing L ligand.

**Scheme 1:**
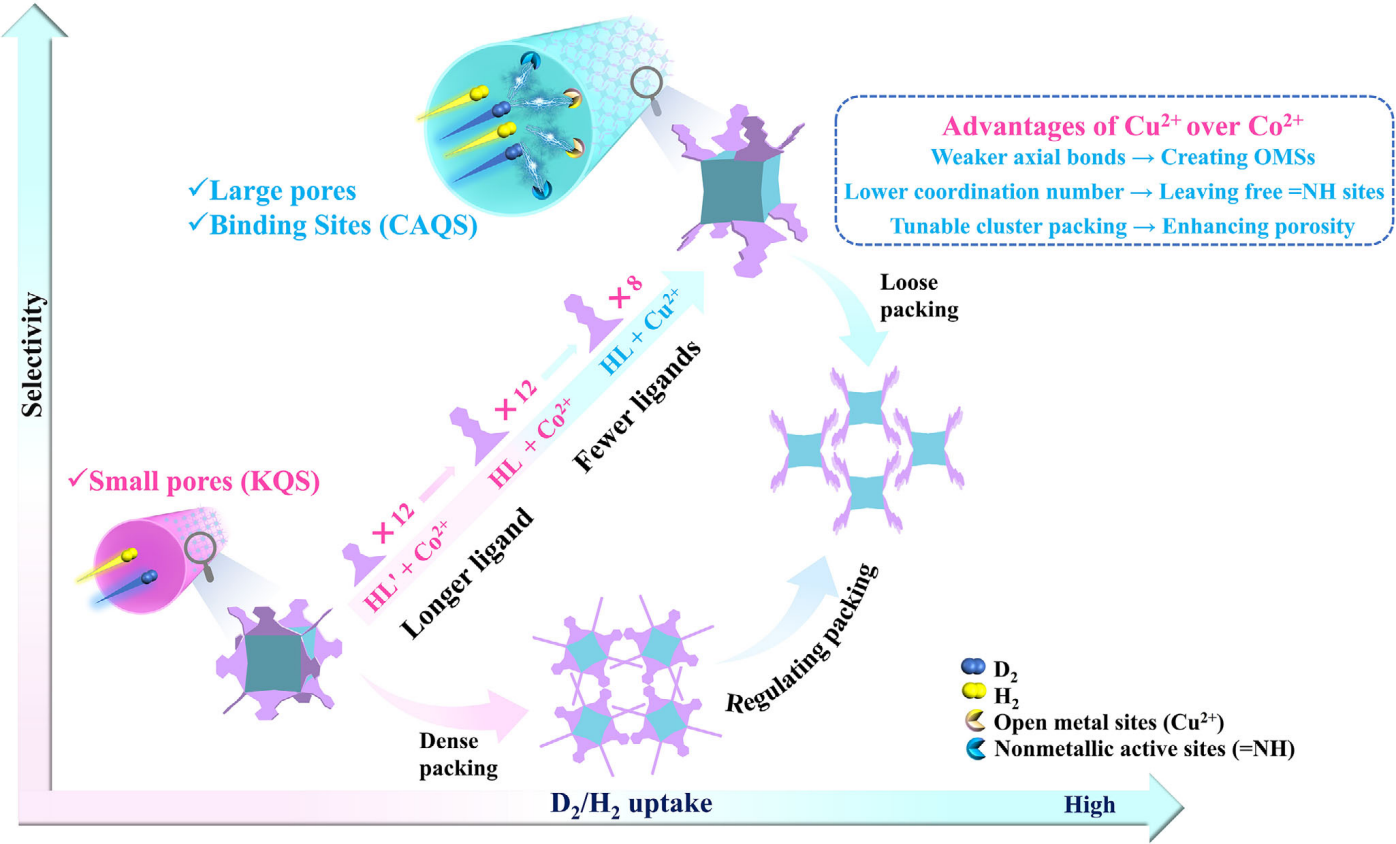
The strategy of engineering pore diameter and incorporating both metal and non‐metal sites into a SAF to optimize D_2_/H_2_ uptake and separation. This strategy is achieved in this work by using copper with a lower coordination number and longer ligand L to replace cobalt and L', respectively, to construct a SAF of metal‐hydroxide clusters.

A key challenge in this approach is the deliberate creation of active adsorption sites. To address this, we propose using copper ions to replace cobalt ions to construct metal‐hydroxide clusters with L (**Scheme** **1**). This strategy offers several potential advantages. First, the Jahn–Teller distortion characteristic of Cu^2+^ ions can weaken axial bonds to solvent molecules, facilitating the formation of open metal sites (OMSs) upon activation.^[^ ^26^ ^]^ Such OMSs serve as high‐affinity adsorption centers, significantly enhancing D_2_/H_2_ uptake. For instance, MOF‐505 exhibits a high H_2_ uptake of 277 cm^3^·g^‒1^ at 77 K and 100 kPa,^[^ ^26^ ^]^ and Cu^2+^–H_2_ interactions have been directly observed in fully desolvated MOFs.^[^ ^27^ ^]^ The strong affinity of coordinatively unsaturated metal sites for hydrogen isotopes is further supported by computational studies.^[^ ^28^ ^]^ Second, the typically lower coordination number of Cu^2+^ ions may allow ligands to retain accessible coordination sites that function as active adsorption centers,^[^ ^20^, ^26^, ^29^ ^]^ while also enabling the formation of clusters with fewer ligands. Finally, the distinct structural characteristics of copper‐based clusters may promote altered packing modes between clusters, potentially leading to SAFs with enhanced porosity. Through this metal‐substitution strategy, the integration of both metal and non‐metal active sites into porous SAFs becomes feasible, offering a promising route for developing high‐performance materials for D_2_/H_2_ uptake and separation.

In this work, we report two SAFs based on metal‐hydroxide cluster obtained via metal centers mediated coordination of ligand L: [Co^II^_12_Co^III^_8_L_12_(*µ*_3_‐OH)_24_]·12(NO_3_) (**1**) and [Cu_20_L_8_(*µ*_3_‐OH)_24_(H_2_O)_8_]·2(C_2_H_6_NH_2_)·5(ClO_4_)·2Cl·3(CHO_2_) (**2**). Both compounds show permanent porosity, and activated **1** exhibits a dense corner‐to‐corner packing, whereas activated **2** shows a loosely organized slipped edge‐to‐edge arrangement. Due to the reduced number of ligands per cluster and its looser packing, activated **2** demonstrates a significantly larger BET surface area compared to activated **1**. More significantly, both active Cu^2+^ and = NH adsorption sites are successfully incorporated into the pores of **2** enabling the second‐highest D_2_ uptake capacity as well as high‐performance D_2_/H_2_ separation.

## 2 Results and Discussion

### 2.1 Crystal Structures and Characterizations of 1 and Activated 1

**Figure 1:**
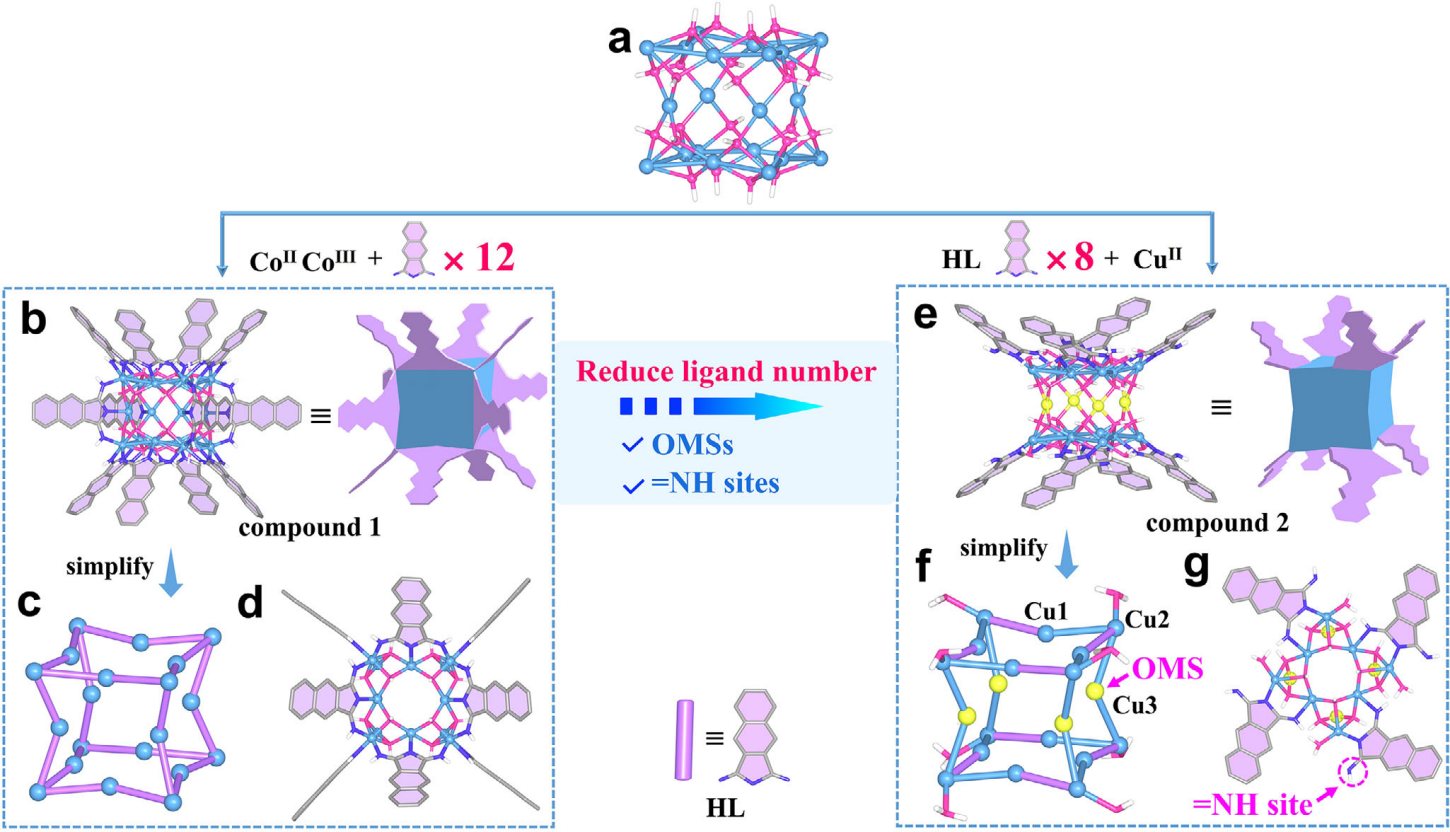
a) [Co_12_^II^Co_8_^III^(*µ*_3_‐OH)_24_]^24+^ cube in compound 1 or [Cu_20_(*µ*_3_‐OH)_24_]^16+^ cube in compound 2. b) [Co^II^_12_Co^III^_8_L_12_(*µ*_3_‐OH)_24_]^12+^ in compound 1. c) Simplified cube core of [Co^II^_12_Co^III^_8_L_12_(*µ*_3_‐OH)_24_]^12+^ in compound 1. d) Top view of the structure of [Co^II^_12_Co^III^_8_L_12_(*µ*_3_‐OH)_24_]^12+^. e) [Cu_20_L_8_(*µ*_3_‐OH)_24_(H_2_O)_8_]^8+^ in compound 2. f) Simplified cube core of [Cu_20_L_8_(*µ*_3_‐OH)_24_(H_2_O)_8_]^8+^. g) Top view of the structure of [Cu_20_L_8_(*µ*_3_‐OH)_24_(H_2_O)_8_]^8+^. Color: blue, Co^II^, Co^III^, Cu^II^; purple, N; pink, O; grey, C; white, H; yellow, OMS.

Our recently reported SAF **3** exhibits only moderate D_2_/H_2_ uptake and separation due to its small pore size (Figure S1a, Supporting Information).^[^ ^25^ ^]^ Therefore, we initially use HL to replace HL' to synthesize SAFs of cobalt‐hydroxide clusters with larger pores (Figure S2, Supporting Information). The solvothermal reaction of Co(NO_3_)_2_·6H_2_O and HL in a mixture of DMF/EtOH (1:1 v/v) with 0.01 mL dimethylamine (DMA) yielded red crystals of **1** (**Figures** **1**b; S3a, Tables S1, S3, and S4, Supporting Information). Single‐crystal X‐ray crystallography (SCXRD) reveals that **1** features fused funnel‐shaped metal‐hydroxide clusters with a cuban‐shaped metal core (Figure 1a–c). X‐ray photoelectron spectroscopy (XPS) reveals that mixed‐valent Co^2+^ and Co^3+^ coexist in **1** (Figure S4, Supporting Information).^[^ ^30^ ^]^ The metal‐hydroxide cluster bearing twelve L ligands is iso‐structural to that in **3**.^[^ ^25^ ^]^ L ligands in **1** are tridentate, with the indole N atom binding to one Co^II^ on an edge and each of the two imine N atoms binding to one apical Co^III^ (Figure 1b,d). Consequently, neither an open metal site nor an active free = NH group exists in the cluster. The clusters pack in a body‐centered cubic (BCC) pattern in a lattice via *π*···*π* interactions (centroid‒centroid distance: 3.76 Å), resulting in a porous SAF (Figures S5a and S6, Supporting Information).

### 2.2 Structure of 2 and D_2_/H_2_ Adsorption and Separation of Activated 1 and 2

**Figure 2:**
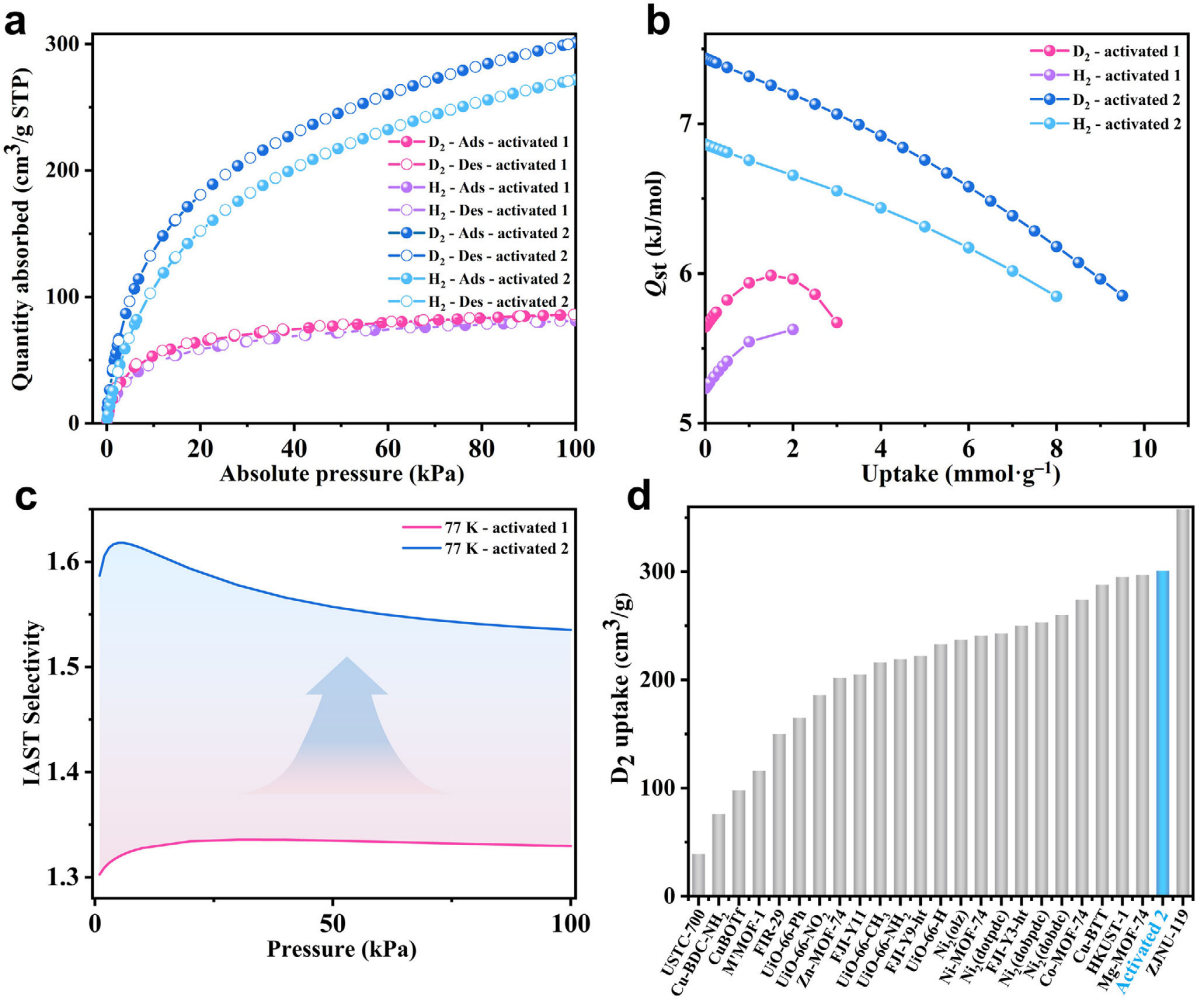
a) D_2_ and H_2_ sorption isotherms of activated 1 and 2 at 77 K. b) Isosteric heats adsorption of D_2_ and H_2_ for activated 1 and 2. c) IAST selectivities of D_2_/H_2_ (50/50 v/v) for activated 1 and 2 at 77 K. d) Comparison of activated 2 (blue) with various other adsorbents on D_2_ adsorption capacity at 77 K and 100 kPa.

Unexpectedly, **1** undergoes an aggressive transition from the space group of *P*2_1_/*n* to *P* $\bar{4}$3*m* upon desolvation, giving rise to activated **1**, as evidenced by both SCXRD and PXRD measurements (Tables S1, S5, and S6, Figure S7, Supporting Information). This transition may be attributed to that adjacent clusters are connected solely by weak *π*···*π* interactions. During the transition, the inter‐cluster *π*···*π* interactions are disrupted, leading to cluster reorientation and significant lattice contraction (Figure S5, Supporting Information). The nearest two neighboring clusters change from edge‐to‐edge to corner‐to‐corner which is the packing of the metal‐hydroxide clusters in **3**, and an extremely high lattice volume contraction of 31% is observed (Figure S8a–c, Supporting Information). As a result for the transition, the central cavities formed by four metal‐hydroxide clusters are significantly obstructed by L ligands (Figure S1, Supporting Information). As estimated by Zeo++ software,^[^ ^31^ ^]^ the accessible pore volume decreases from 51.3% in **1** to 30.7% in activated **1**. Activated **1** exhibits permanent porosity with a BET surface area of 406 m^2^·g^‒1^ and a corresponding pore volume of 0.17 cm^3^·g^‒1^, which are lower than those of activated **3** (696 m^2^·g^‒1^ and 0.27 cm^3^·g^‒1^, respectively) (Figure S9, Supporting Information) probably due to the central channels obstructed in activated **1**.^[^ ^25^ ^]^ Similarly, activated **1** shows lower D_2_/H_2_ uptakes (87 and 82 cm^3^·g^‒1^ for D_2_ and H_2_, respectively, at 77 K, 100 kPa) compared to those of activated **3** (133 and 123 cm^3^/g, respectively) under the same conditions (**Figure** **2**a).^[^ ^25^ ^]^ These results can be attributed to the denser cluster packing in activated **1**. Despite the lower porosity, the pore size of activated **1** (≈10 Å) is indeed notably larger than that of activated **3** owing to the extended length of ligand L and subtle differences in inter‐cluster orientation (Figure S9, Supporting Information).^[^ ^25^ ^]^ The zero‐coverage adsorption enthalpies (*Q_st_*) are calculated to be 5.63 and 5.24 kJ·mol^‒1^ for D_2_ and H_2_, respectively (Figure 2b), and the ideal adsorption solution theory (IAST) selectivity for D_2_/H_2_ (50/50, v/v) at 77 K and 100 kPa is predicted to be 1.33 for D_2_ over H_2_ (Figure 2c).

In order to exploit high‐performance D_2_/H_2_ uptake and separation in SAFs, we then used Cu(ClO_4_)_2_·6H_2_O to replace Co(NO_3_)_2_·6H_2_O aiming to introduce both metal and nonmetal adsorption sites into porous SAFs and obtained green block‐shaped crystals of [Cu_20_L_8_(µ_3_‐OH)_24_(H_2_O)_8_]·2(C_2_H_6_NH_2_)·5(ClO_4_)·2Cl·3(CHO_2_) (**2**) (Tables S1, S7, and S8, Figure S3b, Supporting Information). The +2 oxidation state of copper is confirmed by XPS spectrum (Figure S10, Supporting Information). SCXRD reveals a Cu^2+^‐hydroxide cluster with a cuban‐shaped core crystallizing in the tetragonal space group *I*4/*mmm* (Figure 1a; Table S1, Supporting Information). The cluster adopts a base‐to‐base bifunnel structure supported by an equatorial {Cu_4_} pillar (Figure 1e). Twenty Cu^2+^ ions exhibit three distinct coordination modes: i) eight edge sites (Cu1) display a distorted square pyramidal N_1_O_4_ coordination (Figure S11a, Supporting Information), ii) eight apical positions (Cu2) adopt a distorted pentagonal N_1_O_4_ geometry (containing one aqua ligand, Figure S11b, Supporting Information), and iii) four equatorial centers (Cu3) show square‐planar coordination with four O atoms as active metal sites (Figures 1f; S11c, Supporting Information). The ligand L adopts a bi‐dentate coordination mode, with the indole N atoms binding to one apical Cu^2+^ (Cu2) via a strong Cu─N (2.063 Å) bond and one of the imine N atoms binding to one edge Cu^2+^ (Cu1) via a weak Cu─N bond (2.367 Å),^[^ ^32^ ^]^ leaving a free = NH group (Figure 1e,g; Table S7, Supporting Information). The ligands from the same funnel are oriented either left or right to bind the edge Cu^2+^ ions, leading to the disorder of L (Figure 1e,g). The presence of formate ions, resulting from DMF hydrolysis, was confirmed by Infrared (IR) spectrum (Figure S12, Supporting Information).^[^ ^25^ ^]^ During the reaction, some ClO_4_^‒^ anions were reduced to Cl^‒^ (Figure S13, Supporting Information).^[^ ^33^ ^]^ The Cu^2+^‐hydroxide clusters serve as 8‐connected nodes in a BCC lattice to associate with its eight neighbors via *π···π* interactions (centroid‒centroid distance: 3.38 Å) and N/C─H···O hydrogen bonds (2.53–2.71 Å) (Figures S14 and S15, Supporting Information), forming a 3D framework with elliptical channels along the *a*/*b*‐axes (Figures S1d and S16, Supporting Information). The accessible pore volume estimated by Zeo++ for activated **2** is ≈45.4%.^[^ ^31^ ^]^

**Figure 3:**
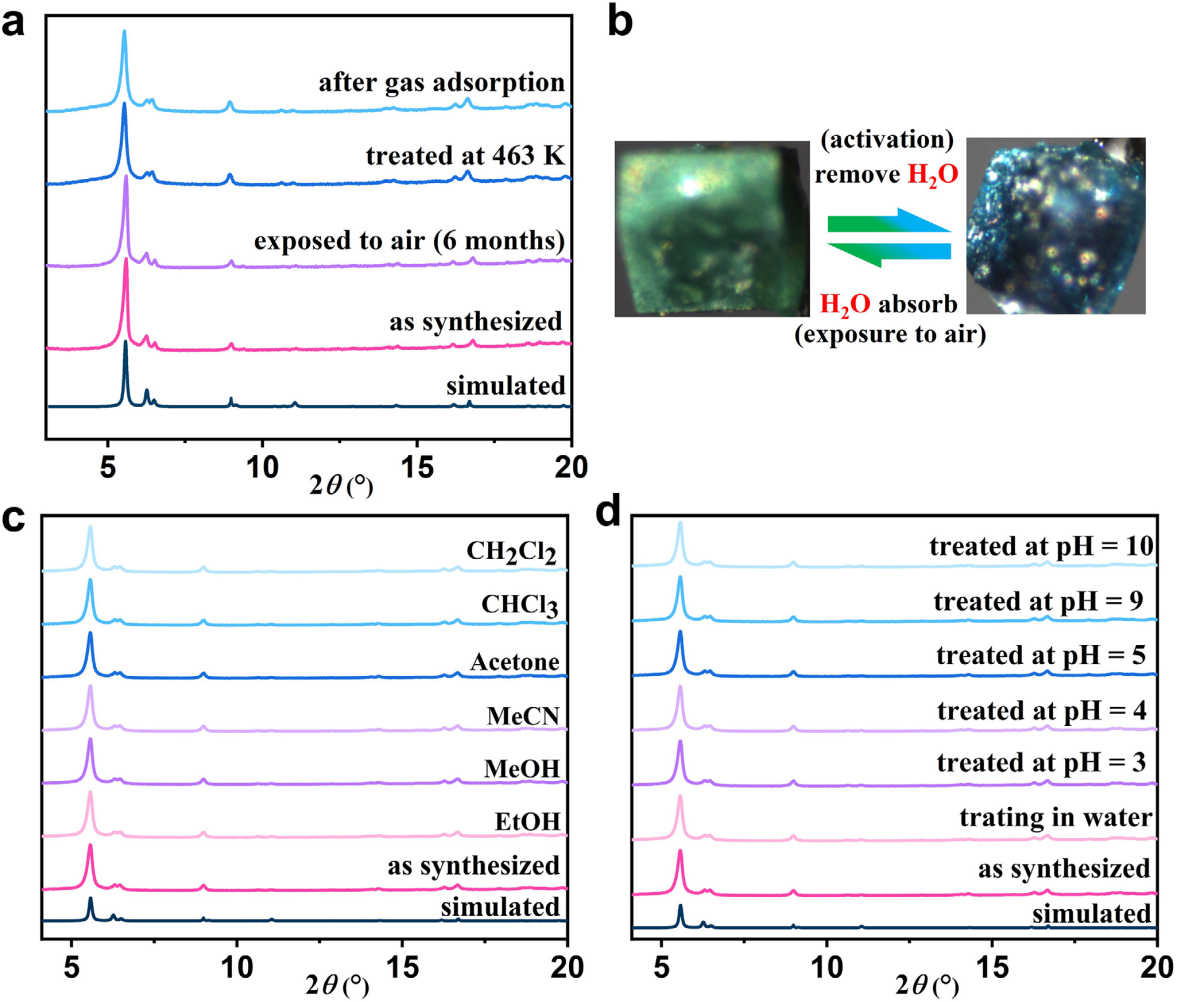
a) PXRD patterns of 2 after treatment under various conditions. b) Reversible crystal color change of 2 upon activation and exposure to ambient conditions. c) and d) PXRD patterns of 2 after treatment with common organic solvents and aqueous solution with various pH for 12 h, respectively.

TG analysis reveals that compound **2** remains stable up to ≈190 °C, and the ≈5.2% weight loss before 190 °C is attributed to the removal of one DMF molecule and eight water molecules, confirming structural integrity after desolvation (**Figures** **3**a; S17, Supporting Information). Upon activation, **2** undergoes a reversible color change from pale green to dark blue (Figure 3b), analogous to that for FJI‐Y11 and HKUST‐1,^[^ ^20^, ^34^ ^]^ but no structural transition was observed for **2** upon desolvation (Figure 3a). This phenomenon indicates the removal of the coordinated H_2_O molecules upon desolvation. The activated dark‐blue sample reverts to its original pale green color within one week under ambient conditions (Figure 3b). Compound **2** also exhibits excellent stability in various common organic solvents, water, and acidic/alkaline solutions, as evidenced by PXRD (Figure 3c,d).

Activated **2** displays a Type I N_2_ adsorption isotherm with a capacity of 467 cm^3^·g^‒1^ at 77 K and a BET surface area of 1670 m^2^·g^‒1^, corresponding to a pore volume of 0.72 cm^3^·g^‒1^. Pore size distribution features a dominant peak at ≈11 Å (Figure S18, Supporting Information). Remarkably, the BET surface area for the sample of **2** preserved under the ambient condition for 6 months is almost the same as that of the freshly synthesized sample (Figure S19, Supporting Information). Because of the outstanding stability and permanent porosity of **2**, we next studied its D_2_/H_2_ adsorption properties. Activated **2** shows extremely high D_2_/H_2_ uptakes (301 and 278 cm^3^·g^‒1^ for D_2_ and H_2_, respectively, at 77 K and 100 kPa), more than three times those of activated **1** (Figure 2a). At 87 K and 100 kPa, the D_2_/H_2_ uptakes for activated **2** remain as high as 219 and 199 cm^3^·g^‒1^, respectively (Figure S20, Supporting Information). As far as we know, the measured D_2_ uptake of activated **2** under the same conditions is surpassed only by ZJNU‐119 (358 cm^3^·g^‒1^)^[^ ^35^ ^]^ and exceeds those of prominent porous materials, including MOF‐74‐Mg (297 cm^3^·g^‒1^),^[^ ^36^ ^]^ HKUST‐1 (295 cm^3^·g^‒1^),^[^ ^20^, ^37^ ^]^ Cu‐BTT (288 cm^3^·g^‒1^),^[^ ^38^ ^]^ MOF‐74‐Co (274 cm^3^·g^‒1^),^[^ ^37^ ^]^ etc (Figures 2d; S21, and Table S2, Supporting Information). Across the pressure of 0–100 kPa, D_2_ uptake consistently exceeds that of H_2_ at both 77 K and 87 K, suggesting the presence of significant quantum sieving effects (Figure 2a). The zero‐coverage *Q_st_* for activated **2** (7.45 and 6.85 kJ·mol^‒1^ for D_2_ and H_2_, respectively) exceeds that of activated **1**, indicating stronger D_2_/H_2_ adsorption sites in activated **2**. Moreover, the *Q_st_* values decrease rapidly with increasing gas loading, consistent with the presence of high‐energy binding sites that are occupied at low pressures (Figure 2b).^[^ ^39^ ^]^ In contrast, activated **1** shows an initial rise in *Q_st_* followed by a decline, implying a lack of defined strong adsorption sites (Figure 2b). The initial increase may be attributed to pore contraction during gas adsorption, enhancing framework–gas interactions. The IAST selectivity for D_2_ over H_2_ (50/50 mixture) reaches 1.53 at 77 K and 100 kPa for activated **2**, significantly higher than the value of 1.33 for activated **1** under the same conditions (Figure 2c). These differences can be attributed to the OMSs and free = NH groups in activated **2**, which show higher affinities for D_2_.

**Figure 4:**
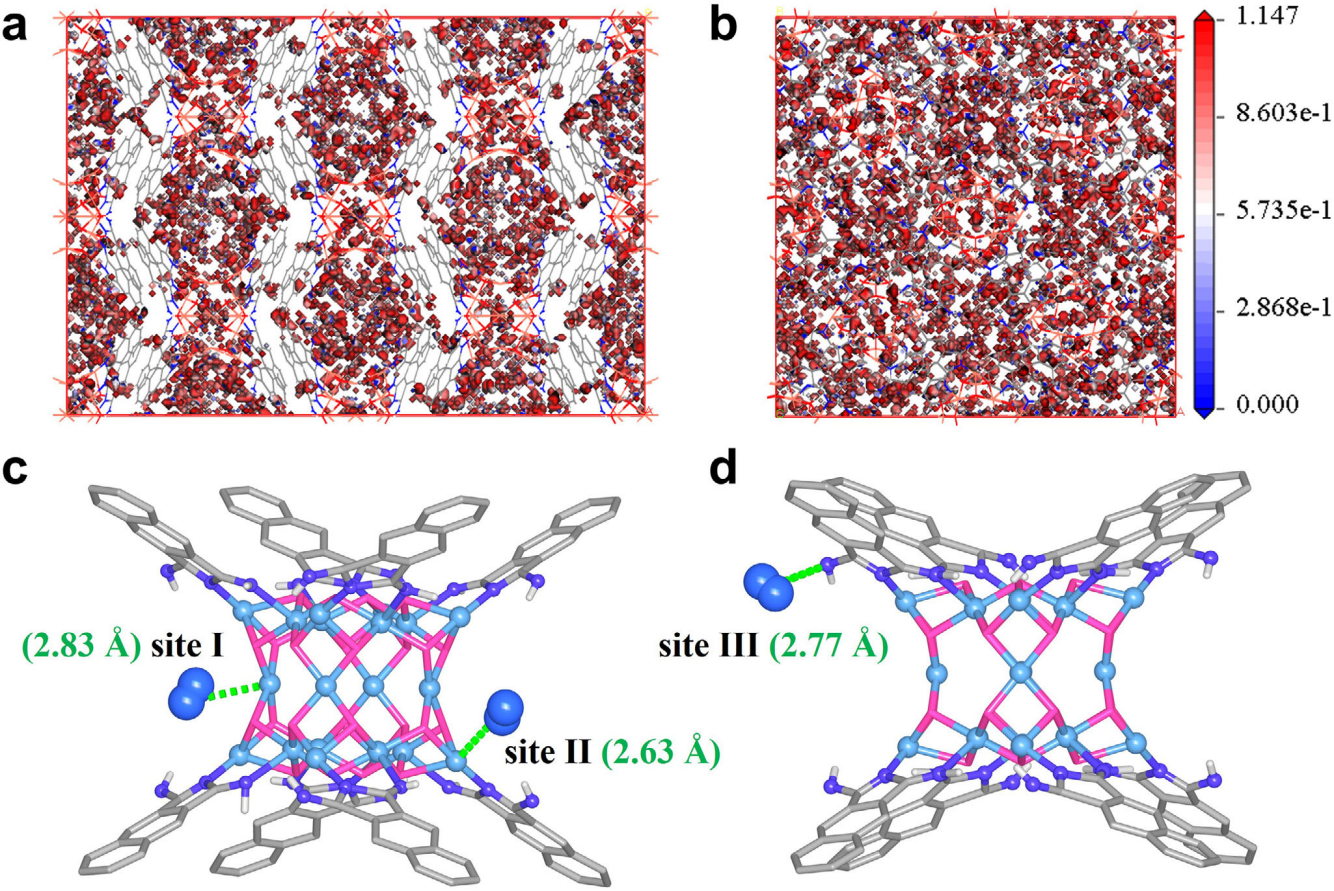
Simulated adsorption sites in activated 2 obtained from the GCMC simulation. For clarity, some atoms are omitted, and D_2_ molecules are shown in a space‐filling model.

To elucidate the specific binding sites for D_2_ and H_2_ in activated **2**, we performed Grand Canonical Monte Carlo (GCMC) simulations using Materials Studio software.^[^ ^40^ ^]^ The simulated adsorption density profiles revealed three distinct preferential adsorption sites (**Figures** **4**; S22, Supporting Information). These three sites are the equatorial Cu^2+^ (site I), the apical Cu^2+^ (site II), and the free = NH group (site III), which potentially bind D_2_ with Cu─D, Cu─D, and N─D distances of 2.83, 2.63, and 2.77 Å, respectively (Figure 4c,d). The average adsorption energies for D_2_ and H_2_ are calculated to be 1.586 and 1.580 kcal·mol^‒1^, respectively, corresponding to uptakes of 666 D_2_ molecules and 656 H_2_ molecules per unit cell. These results clearly demonstrate that the OMSs and free = NH groups in **2** are critical to the observed extremely high D_2_/H_2_ uptake.

### 2.3 Dynamic Column Breakthrough Experiments

**Figure 5:**
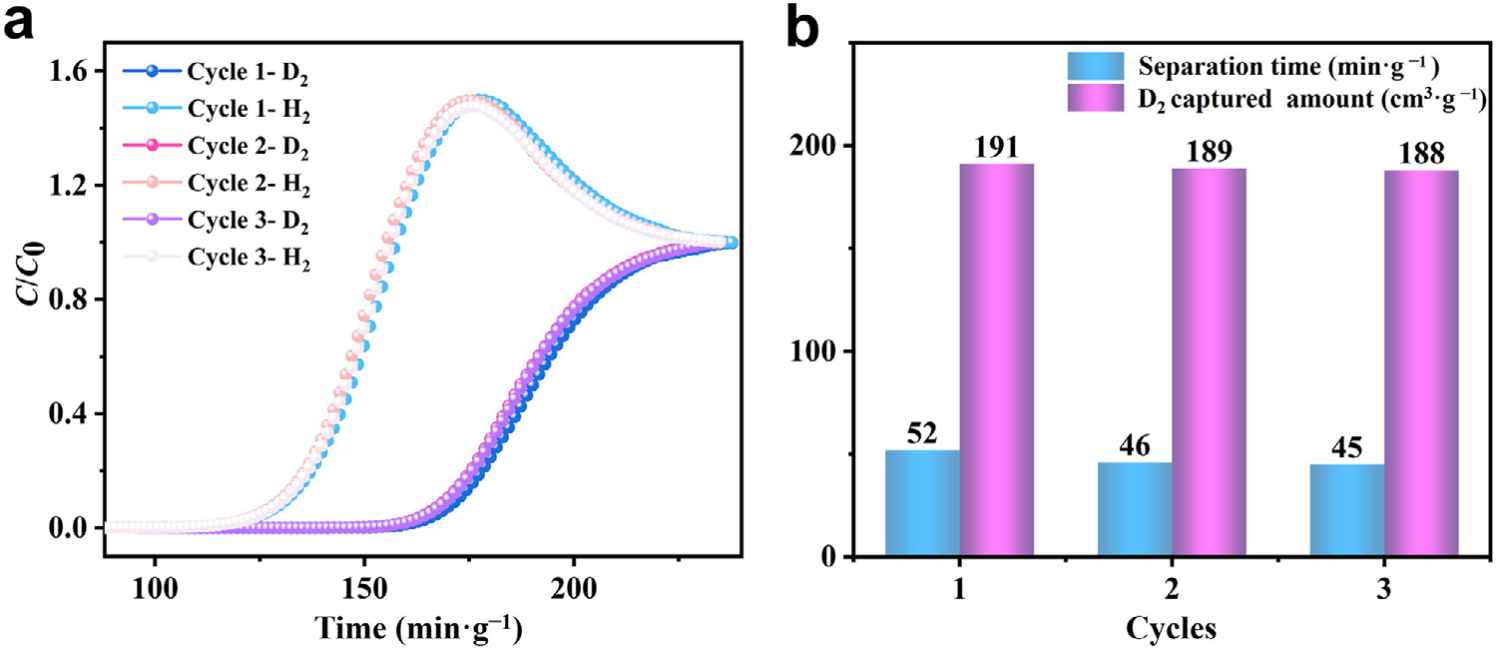
a) Dynamic breakthrough curves for D_2_/H_2_/Ne (2.5/2.5/95 vol%) on activated **2** at 77 K (total flow: 8 mL·min^‒1^). b) Retention lags and corresponding amounts of captured D_2_ for three consecutive cycles.

Motivated by the exceptional D_2_/H_2_ adsorption capacity of activated **2**, breakthrough experiments were performed at 77 K and 100 kPa to systematically assess its hydrogen isotope separation performance. A ternary gas mixture D_2_/H_2_/Ne (2.5/2.5/95, vol.%) was introduced into an activated **2** packed adsorption column at a total flow rate of 8 mL·min^‒1^. The resulting breakthrough curves indicate that H_2_ breaks through the adsorption bed first, followed by a time lag before D_2_ slowly elutes (**Figure** **5**a). The retention lag reached 52 min·g^‒1^, significantly exceeding that reported for Cu‐BTT (separation time: 16 min·g^‒1^ under the same conditions).^[^ ^38^ ^]^ The dynamic capture amounts of D_2_ and H_2_ are calculated to be ≈191 and ≈127 cm^3^·g^‒1^, respectively (Figure 5b), yielding a separation factor of 1.52 at 100 kPa, consistent with the value of 1.53 predicted by IAST. Notably, the D_2_/H_2_ adsorption capacities and retention lag remained virtually unchanged over three consecutive adsorption–desorption cycles, demonstrating excellent recyclability and potential for practical industrial D_2_/H_2_ separation (Figure 5). Furthermore, **2** retains structural integrity after the breakthrough experiment as indicated by the PXRD measurements (Figure 3a).

## 3 Conclusion

In summary, two new SAFs based on metal‐hydroxide clusters were synthesized via ligand coordination mediated by different metal centers (Co^2+^ and Cu^2+^). **2** shows exceptional thermal/chemical stability as well as high porosity. In particular, the open Cu^2+^ sites and active = NH groups impart activated **2** for the second‐highest D_2_ uptake and high performance of D_2_/H_2_ separation. These results demonstrate that integrating multiple active adsorption sites into a porous structure is a promising strategy for exploiting new high‐performance D_2_/H_2_ separation materials.

## Conflict of Interest

The authors declare no competing financial interest.

## Supporting information



Supporting Information

Supporting cif files

## Data Availability

The data that support the findings of this study are available in the supplementary material of this article.
