# Supplementary material for: Engineering Active Metal and Nonmetal Sites in Porous Structures of Metal‐Hydroxide Clusters for Enhanced D2/H2 Uptake and Separation
Source: Adv Sci (Weinh). 2025 Dec 14;13(13):e19498. doi: 10.1002/advs.202519498 (PMC12955987; doi:10.1002/advs.202519498)
Supplement: Supplementary file 1 — Supporting Information [file ADVS-13-e19498-s002.docx]

Supporting Information

**Engineering Active Metal and Nonmetal Sites in Porous Structures of Metal-Hydroxide Clusters for Enhanced D_2_/H_2_ Uptake and Separation**

*Zhuozhou Xie, Zhu Zhuo,* Zi-Ang Nan, Qing Li, Wenlin Wu, Yunzhe Zhou, Zi-Xiu Lu, Jin Liu, Luyao Liu, Wenjing Wang,* Daqiang Yuan, You-Gui Huang**

**Contents**

1. **Materials and Methods**
2. **Supporting Tables**
3. **Supporting Figures**
4. **Supporting References**
5. **Materials and Methods.**
   1. **Materials.**

1H-Benzo[f]isoindole‒1,3-diimine (HL), Co(NO_3_)_2_·6H_2_O, Cu(ClO_4_)_2_·6H_2_O, dimethylamine (DMA), ethanol, N,N-dimethylformamide (DMF), and all other chemicals are analytical-reagent grade and used as purchased without further purification.

- 1. **Synthesis of compounds 1 and 2.**

Synthesis of [Co^II^_12_Co^III^_8_L_12_(*μ*_3_-OH)_24_]·12(NO_3_) (**1**):

Co(NO_3_)_2_·6H_2_O (0.18 g, 0.62 mmol) and HL (0.069 g, 0.087 mmol) were added into a mixture of DMF/EtOH (3 mL) (1:1, v/v) with 0.01 mL DMA. After being stirred for 1 day, a cloudy blue solution was obtained. The resulting solution was transferred into a 50 mL Teflon-lined stainless steel vessel. The vessel was heated at 140 °C for 5 days. After cooling to room temperature at 5 °C/h, the resulting solution was kept undisturbed at the ambient condition for two months. Red crystal of compound **1** were obtained and cleaned by CH_2_Cl_2_. Yield: 66 % (based on HL).

Synthesis of [Cu_20_L_8_(*μ*_3_-OH)_24_(H_2_O)_8_]·2(C_2_H_6_NH_2_)·5(ClO_4_)·2Cl·3(CHO_2_) (**2**):

The synthesis procedure for compound **2** is similar to that of compound **1**, except replacing Co(NO_3_)_2_·6H_2_O with Cu(ClO_4_)_2_·6H_2_O. Green block-shaped crystals of **2** were harvested and cleaned by EtOH. Yield: 36 % (based on HL).

- 1. **Physical measurements.**

Powder X-ray diffraction (PXRD) patterns were collected on a Rigaku Miniflex 600 diffractometer with Cu-*K*α radiation using flat plate geometry. Thermogravimetric analysis (TGA) was performed under argon on a Mettler-Toledo TGA/DSC 1 system with a heating rate of 10 °C min^‒1^. X-ray photoelectron spectroscopies (XPS) studies were performed on a ThermoFisher ESCALAB 250 Xi system and the C1s line at 284.8 eV was used as the binding energy reference.

- 1. **Crystallography.**

Single-crystal XRD data were collected at 293 K (**1**), 300 K (activated **1**), and 100 K (**2**). Structures were solved using a direct method and refined by the full-matrix least-squares technique on *F*^2^ with SHELXTL 2014 program. All non-hydrogen atoms were refined anisotropically, and hydrogen atoms were located at their calculated positions. The X-ray crystallographic coordinates for structures reported in this article have been deposited at the Cambridge Crystallographic Data Centre (CCDC), under deposition numbers **CCDC (2477514-2477516)**. These data can be obtained free of charge from The Cambridge Crystallographic Data Centre via www.ccdc.cam.ac.uk/data_request/cif. Detail crystallographic data are listed in Supporting Information Table S1.

- 1. **Isosteric Enthalpy of Adsorption.**

The isosteric enthalpies of adsorption for H_2_ and D_2_ were calculated using the isotherms at 77 K and 87 K, following the Viral equation:

$$\ln P=\ln N+\frac{1}{T}\sum_{i=0}^{m} a_{i}N^{i}+\sum_{i=0}^{n} b_{i}N^{i}$$

where *P* is the pressure (mmHg), *N* is the capacity adsorbed (mg·g^‒1^), *T* is the temperature (K), *a*_i_ and *b*_j_ represents Viral coefficients, and *m* and *n* are the number of coefficients used to describe the isotherms. The values of the Virial coefficients *a_0_* to *a_m_* were then used to calculate the Isosteric enthalpy of adsorption according to the following equation:

$$Q_{st}=-R\sum_{i=0}^{m} a_{i}N^{i}$$

*Q_st_* is the isosteric enthalpy of adsorption in kJ·mol^‒1^ and *R* is universal gas constant with the value of 8.314 J·mol^‒1^·K^‒1^.

- 1. **Calculation of the selectivity by IAST.**

Single-component gas equilibrium adsorption isotherms were fitted with the Langmuir–Freundlich model, given by the following equation:

$$N=A_{1}\frac{b_{1}p^{c_{1}}}{1+b_{1}p^{c_{1}}}+A_{2}\frac{b_{2}p^{c_{2}}}{1+b_{2}p^{c_{2}}}$$

where *N* is the amount of adsorbed gas (mmol·g^–1^), *p* is the bulk gas phase pressure (atm), *A_1_* and *A*_2_ are the adsorption saturation capacities for sites 1 and 2 (mmol·g^–1^), *b_1_* and *b*_2_ are the affinity coefficients of sites 1 and 2 (1/kPa), *c_1_* and *c*_2_ are the Freundlich indices.

The parameters of *A_1_*, *A_2_*, *b_1_*, *b_2_*, *c_1_,* and *c_2_* were used to predict the adsorption selectivities based on IAST, which is finally defined as:

$$S_{\frac{1}{2}}=(\frac{x_{i}}{x_{2}})(\frac{y_{2}}{y_{i}})$$

where *S* is the ideal selectivity of component 1 over component 2, *x_i_* and *y_i_* are the mole fractions of component *i* (*i* = 1, 2) in the adsorbed and bulk phases, respectively.

- 1. **Breakthrough measurements.**

On the basis of mass balance, the gas adsorption amounts based on breakthrough experiments can be defined as the following:

$$q_{i}=\frac{C_{i}V}{22.4\times m}\times\int_{0}^{t} \left( 1-\frac{F}{F_{0}} \right)dt$$

Among them, *q*_i_ refers to the equilibrium adsorption capacity of gas component i (mmol g^‒1^), *C*_i_ represents gas concentration, *V* represents gas flow rate (cm^3^ min^‒1^), *t* represents adsorption time (min), *F*_0_ and *F* represent gas molar flow rates at the inlet and outlet, respectively, and *m* represents mass (g). Separation factor (*α*) for a penetration experiment can be calculated based the following equation:

$$\alpha=\frac{q_{A}y_{B}}{q_{B}y_{A}}$$

*y_i_* represents the molar component *i* of gas *i* in the mixed gas (*i* = A, B).

- 1. **Single-component equilibrium adsorption isotherms.**

Single-component gas equilibrium adsorption isotherms were measured at pressures from 0 to 105 kPa using automatic volumetric adsorption equipment (Micromeritics ASAP 2020+), which uses a volumetric method to determine gas adsorption capacity at equilibrium pressure. Before gas sorption measurements, as-synthesized samples after solvent exchange with dichloromethane for six times over three days were degassed under dynamic vacuum (≤ 10 μm Hg) at a heating rate of 4 °C/min to 100 °C for 5 h. For all gas equilibrium adsorption isotherms, warm and cool free-space were measured utilizing ultra-high purity He (> 99.9999%). The specific surface area of activated **1** and **2** were determined by the Brunauer-Emmett-Teller (BET) model from the N_2_ adsorption isotherm data between the relative pressure of 0.05 and 0.3 at 77 K. Pure component adsorption isotherms of H_2_ and D_2_ at 77 K were collected using liquid nitrogen to keep the sample temperature. After each isotherm measurement, the sample was reactivated at a heating rate of 4 °C/min until 160 °C under dynamic vacuum for 30 min.

- 1. **Dynamic column breakthrough separation experiments.**

The breakthrough curves were measured on a real-time low temperature apparatus coupled with a mass spectrometer (PFEIFFER VACUUM) using a mixture gas of D_2_/H_2_/Ne (2.5/2.5/95, vol.%) at 77 K and 100 kPa. In a typical breakthrough experiment, a dry sample of compound **2** (0.8 g) was packed into a stainless-steel column (diameter = 2 mm, length = 110 mm) with silica wool filling the void space. The column filled with the sample was degassed under dynamic vacuum at 373 K for 5 h, and then a constant Ne flow (10 mL·min^‒1^) was used to purge the column for 2 h at 373 K. To ensure the accuracy of the breakthrough experiment, the column and pre-cooling line was immersed into a liquid nitrogen tank for 20 min before measurements. The gas mixtures of D_2_/H_2_/Ne (2.5/2.5/95, vol.%) were passed through the packed column at a flow rate 8 mL·min^‒1^. The gas flow was controlled by a mass flow meter at the inlet and the composition of the effluent gas from the column was continuously monitored by mass spectrometry. After each measurement, the sample was regenerated with a Neon flow (10 mL·min^‒1^) at 373 K for 30 min.

- 1. **Evaluation of the binding sites of H_2_ and D_2_ in activated 2.**

We used the grand canonical Monte Carlo (GCMC) module with the Universal force field in the Materials Studio software to simulate the distribution of the D_2_ and H_2_ gases in activated **2** under the conditions of 77 K and 100 kPa. The crystal structure of activated **2** was chosen for related simulations without further geometry optimization. The cutoff radius of 12.5 Å was used to handle the nonbonding interactions, and the Ewald & Group summation method was applied to calculate the long-range electrostatic interactions. Each state point of GCMC simulations contained 1 × 10^4^ steps to guarantee equilibration followed by 1 × 10^5^ steps to sample the required thermodynamics properties.

1. **Supporting Tables.**

**Table S1** Crystallographic data of the synthetic compounds.

|  | **1** | activated **1** | **2** |
| --- | --- | --- | --- |
| formula | C_144_H_120_Co_20_O_60_N_48_ | C_144_H_120_Co_20_O_60_N_48_ | C_103_H_123_Cu_20_O_58_N_26_Cl_7_ |
| *F*_w_ | 4661.47 | 4661.47 | 4172.42 |
| crystal system | Monoclinic | Cubic | Tetragonal |
| space group | *P*2_1_/*n* | *P*$\bar{\text{4}}$3*m* | *I*4/*mmm* |
| *a* (Å) | 24.5688(4) | 20.7655(10) | 19.5796(2) |
| *b* (Å) | 20.5400(3) | 20.7655(10) | 19.5796(2) |
| *c* (Å) | 25.7456(5) | 20.7655(10) | 28.3746(5) |
| *α* (deg) | 90 | 90 | 90 |
| *β* (deg) | 92.609 | 90 | 90 |
| *γ* (deg) | 90 | 90 | 90 |
| *V* (Å^3^) | 12978.9(4) | 8954.2(13) | 10877.7(3) |
| *Z* | 2 | 2 | 2 |
| *D_c_* / g cm^−3^ | 1.193 | 1.729 | 1.631 |
| *T* (K) | 293.00 | 300.00 | 100.00 |
| *F* (000) | 4680 | 4680 | 5452 |
| collected/unique | 88093/26141 | 8565/2914 | 17203/3415 |
| *R*_int_ | 0.0555 | 0.0686 | 0.0672 |
| GOF on F^2^ | 1.041 | 1.054 | 1.089 |
| *R*_1_ (*I* > 2*σ*) | 0.0900 | 0.1459 | 0.1192 |
| *wR*_2_ (*all*) | 0.2909 | 0.5178 | 0.3225 |
| CCDC# | 2477514 | 2477515 | 2477516 |

^a^ *R* **=** ∑**||***F*_0_**|**−**|***F*_c_**||)/∑|***F*_0_**|**

^b^ *wR* **= |∑***w***(***F*_0_−*F*_c_**)**^2^**/∑***w(F*_0_^2^**)**^2^**|**^1/2^

**Table S2** Comparison of activated **2** with various other adsorbents on D_2_ and H_2_ adsorption capacity and IAST selectivity at 77 K and 100 kPa.

| Adsorbent | D_2_ amount  (cm^3^/g) | H_2_ amount  (cm^3^/g) | IAST  Selectivity | Ref |
| --- | --- | --- | --- | --- |
| ZJNU-119 | 358 | 325 | 1.55 | ^[1]^ |
| Activated **2** | 301 | 278 | 1.53 | ^this work^ |
| Mg-MOF-74 | 297 | 269 | 2.1 | ^[2]^ |
| HKUST-1 | 295 | 277 | 1.42 | ^[3]^ |
| Cu-BTT | 288 | 266 | 1.58 | ^[4]^ |
| Co-MOF-74 | 274 | 258 | 2.2 | ^[2]^ |
| Ni_2_(dobdc) | 260 | 251 | 2.3 | ^[5]^ |
| Ni_2_(dobpdc) | 253 | 231 | 2.25 | ^[5]^ |
| FJI-Y3-ht | 250 | 233 | 1.4 | ^[6]^ |
| Ni_2_(dotpdc) | 243 | 220 | 2 | ^[5]^ |
| Ni-MOF-74 | 241 | 235 | 2 | ^[7]^ |
| Ni_2_(olz) | 237 | 215 | 2.5 | ^[5]^ |
| UiO-66-H | 233 | 211 | 1.31 | ^[8]^ |
| FJI-Y9-ht | 222 | 202 | 1.3 | ^[9]^ |
| UiO-66-NH_2_ | 219 | 201 | 1.38 | ^[8]^ |
| UiO-66-CH_3_ | 216 | 201 | 1.32 | ^[8]^ |
| FJI-Y11 | 205 | 183 | 1.76 | ^[3]^ |
| Zn-MOF-74 | 202 | 190 | 1.6 | ^[2]^ |
| UiO-66-NO_2_ | 186 | 171 | 1.35 | ^[8]^ |
| UiO-66-Ph | 165 | 153 | 1.34 | ^[8]^ |
| FIR-29 | 150 | 137 | 1.2 | ^[9]^ |
| M’MOF-1 | 116 | 106 | 1.38 | ^[10]^ |
| Cu-BDC-NH_2_ | 76 | 61 | 1.6 | ^[11]^ |
| CuBOTf | 98 | 83 | 1.2 | ^[12]^ |
| Activated **1** | 87 | 82 | 1.33 | ^this work^ |
| USTC-700 | 39 | 38 | not reported | ^[13]^ |

**Table S3** Selected bond lengths for compound **1**.

| Atom | Atom | Length (Å) | Atom | Atom | Length (Å) |
| --- | --- | --- | --- | --- | --- |
| Co1 | O33^#1^ | 2.053(5) | Co6 | O32 | 2.058(4) |
| Co1 | O32^#1^ | 2.042(4) | Co6 | O35 | 2.060(4) |
| Co1 | O42 | 2.053(5) | Co6 | N13 | 2.093(5) |
| Co1 | O37 | 2.046(4) | Co7 | O34 | 1.996(4) |
| Co1 | N19 | 2.070(5) | Co7 | O33 | 2.002(4) |
| Co2 | O42 | 1.963(5) | Co7 | O32 | 1.985(5) |
| Co2 | O38 | 1.959(4) | Co7 | N12 | 1.992(6) |
| Co2 | O37 | 1.976(5) | Co7 | N20^#1^ | 1.964(5) |
| Co2 | N17 | 1.946(5) | Co7 | N28 | 2.071(3) |
| Co2 | N21 | 1.942(6) | Co7 | N27 | 1.940(15) |
| Co2 | N18 | 1.945(5) | Co8 | O34 | 2.062(4) |
| Co3 | O39 | 2.058(4) | Co8 | O33 | 2.067(4) |
| Co3 | O42 | 2.053(4) | Co8 | O39 | 2.043(4) |
| Co3 | O38 | 2.068(4) | Co8 | O40 | 2.068(4) |
| Co3 | O41 | 2.073(4) | Co8 | N30 | 2.101(2) |
| Co3 | N22 | 2.069(5) | Co8 | N29 | 2.083(11) |
| Co4 | O36 | 2.072(4) | Co9 | O39 | 1.969(4) |
| Co4 | O38 | 2.074(4) | Co9 | O40 | 1.970(4) |
| Co4 | O37 | 2.039(4) | Co9 | O41 | 1.965(5) |
| Co4 | O35 | 2.042(4) | Co9 | N24 | 1.942(5) |
| Co4 | N16 | 2.073(5) | Co9 | N23 | 1.961(5) |
| Co5 | O31 | 1.983(4) | Co9 | N31 | 1.731(3) |
| Co5 | O36 | 1.961(5) | Co9 | N32 | 2.056(14) |
| Co5 | O35 | 1.974(5) | Co10 | O311 | 2.050(4) |
| Co5 | N26^1^ | 1.969(5) | Co10 | O40 | 2.044(4) |
| Co5 | N15 | 1.966(5) | Co10 | O36^#1^ | 2.056(4) |
| Co5 | N14 | 1.962(5) | Co10 | O41 | 2.059(4) |
| Co6 | O34 | 2.046(4) | Co10 | N25 | 2.081(5) |
| Co6 | O31 | 2.034(5) |  |  |  |

Symmetry code: #1: 1‒X, 1‒Y, 1‒Z.

**Table S4** Selected bond angles for compound **1**.

| Atom | Atom | Atom | Angle (˚) | Atom | Atom | Atom | Angle (˚) |
| --- | --- | --- | --- | --- | --- | --- | --- |
| O36 | Co4 | O38 | 177.0(18) | O39 | Co9 | O40 | 82.3(17) |
| O36 | Co4 | N16 | 91.1(18) | O39 | Co9 | N32 | 93.6(5) |
| O37 | Co4 | O36 | 103.2(18) | O40 | Co9 | N32 | 94.2(6) |
| O37 | Co4 | O38 | 77.9(17) | O41 | Co9 | O39 | 82.4(18) |
| O37 | Co4 | O35 | 176.2(18) | O41 | Co9 | O40 | 82.4(18) |
| O37 | Co4 | N16 | 92.0(18) | O41 | Co9 | N32 | 175.1(5) |
| O35 | Co4 | O36 | 77.4(17) | N24 | Co9 | O39 | 174.8(2) |
| O35 | Co4 | O38 | 101.2(16) | N24 | Co9 | O40 | 94.0(2) |
| O35 | Co4 | N16 | 91.6(17) | N24 | Co9 | O41 | 93.4(2) |
| N16 | Co4 | O38 | 91.5(18) | N24 | Co9 | N23 | 87.9(2) |
| O34 | Co8 | O33 | 78.5(16) | N24 | Co9 | N32 | 90.4(5) |
| O34 | Co8 | O40 | 177.1(18) | N23 | Co9 | O39 | 95.5(2) |
| O34 | Co8 | N30 | 94.7(7) | N23 | Co9 | O40 | 175.2(2) |
| O34 | Co8 | N29 | 91.1(3) | N23 | Co9 | O41 | 93.1(2) |
| O33 | Co8 | O40 | 100.9(17) | N23 | Co9 | N32 | 90.2(6) |
| O33 | Co8 | N30 | 95.5(7) | N31 | Co9 | O39 | 92.3(12) |
| O33 | Co8 | N29 | 90.1(4) | N31 | Co9 | O40 | 93.3(12) |
| O39 | Co8 | O34 | 102.1(15) | N31 | Co9 | O41 | 174.3(12) |
| O39 | Co8 | O33 | 177.4(18) | N31 | Co9 | N24 | 91.5(12) |
| O39 | Co8 | O40 | 78.2(16) | N31 | Co9 | N23 | 90.4(12) |
| O39 | Co8 | N30 | 86.9(7) | O33 | Co1 | N19 | 91.5(18) |
| O39 | Co8 | N29 | 92.1(4) | O32^#1^ | Co1 | O33^#1^ | 79.4(17) |
| O40 | Co8 | N30 | 88.2(7) | O32^#1^ | Co1 | O42 | 101.7(18) |
| O40 | Co8 | N29 | 91.1(3) | O32^#1^ | Co1 | O37 | 178.2(17) |

Symmetry code: #1: 1‒X, 1‒Y, 1‒Z.

**Table S5** Selected bond lengths for activated **1**.

| Atom | Atom | Length (Å) | Atom | Atom | Length (Å) |
| --- | --- | --- | --- | --- | --- |
| Co1 | O8 | 2.110(13) | Co4 | O10 | 2.081(4) |
| Co2 | O7 | 2.090(17) | Co5 | O9 | 2.027(16) |
| Co2 | O8 | 2.127(11) | Co5 | N7 | 2.063(3) |
| Co3 | O7 | 2.052(3) | Co5 | O10 | 1.919(14) |
| Co3 | N3 | 1.932(4) | Co6 | O9 | 1.801(3) |
| Co4 | N6 | 1.793(4) | Co6 | N8 | 1.951(4) |

**Table S6** Selected bond angles for activated **1**.

| Atom | Atom | Atom | Angle (˚) | Atom | Atom | Atom | Angle (˚) |
| --- | --- | --- | --- | --- | --- | --- | --- |
| O9^#1^ | Co5 | O9 | 70.1(15) | O8 | Co1 | O8^#6^ | 86.6(11) |
| O9^#1^ | Co5 | N7 | 92.6(15) | O10^#3^ | Co4 | O10 | 68.3(17) |
| O9 | Co5 | N7 | 95.2(16) | O9 | Co6 | O9^#1^ | 80.6(14) |
| O10 | Co5 | O9 | 105.2(11) | O9 | Co6 | N8 | 91.0(2) |
| O10 | Co5 | O9^#1^ | 164.1(12) | O9^#1^ | Co6 | N8 | 90.0(2) |
| O10 | Co5 | N7 | 103.0(17) | O9^#4^ | Co6 | N8 | 168.0(2) |
| O10^#2^ | Co5 | N7 | 100.2(19) | O7 | Co2 | O7^#1^ | 83.8(17) |
| O10 | Co5 | O10^#2^ | 75.2(2) | O7^#1^ | Co2 | O8 | 95.2(9) |
| N6 | Co4 | O10^#2^ | 99.0(18) | O7 | Co2 | O8^#5^ | 95.2(9) |
| N6 | Co4 | O10 | 107.5(19) | O7 | Co2 | O8 | 177.4(9) |
| N6 | Co4 | O10^#3^ | 167.3(19) | O8^#5^ | Co2 | O8 | 85.7(10) |
| N3 | Co3 | O7^#4^ | 92.0(2) | O7^1^ | Co3 | O7 | 85.9(14) |
| N3 | Co3 | O7^#1^ | 95.0(3) |  |  |  |  |

Symmetry codes: #1: +Y, +Z, +X; #2: +Y, 1‒Z, 1‒X; #3: 1‒Z, +X, 1‒Y; #4: +Z, +X, +Y; #5: +Z, ‒X, ‒Y; #6: ‒Y, ‒Z, +X.

**Table S7** Selected bond lengths for compound **2**.

| Atom | Atom | Length (Å) | Atom | Atom | Length (Å) |
| --- | --- | --- | --- | --- | --- |
| Cu1 | O3 | 1.985(6) | Cu2 | O1^#2^ | 2.312(8) |
| Cu1 | O1 | 1.950(4) | Cu3 | O3 | 1.943(6) |
| Cu1 | N3 | 2.403(10) | Cu3 | O7 | 2.350(10) |
| Cu2 | O3 | 2.002(6) |  |  |  |

Symmetry code: #2: +Y, 1‒X, +Z.

**Table S8** Selected Bond Angles for compound **2**.

| Atom | Atom | Atom | Angle (˚) | Atom | Atom | Atom | Angle (˚) |
| --- | --- | --- | --- | --- | --- | --- | --- |
| O3 | Cu1 | O3^#1^ | 97.1(4) | O3 | Cu2 | O12 | 74.5(2) |
| O3^#1^ | Cu1 | N3 | 82.9(6) | O3 | Cu3 | O3^#5^ | 168.4(4) |
| O3 | Cu1 | N3 | 83.9(6) | O3^#5^ | Cu3 | O3^#4^ | 80.4(4) |
| O1 | Cu1 | O3 | 178.5(3) | O3^#5^ | Cu3 | O3^#3^ | 98.5(4) |
| O1^#2^ | Cu1 | O3 | 83.6(3) | O3 | Cu3 | O3^#4^ | 98.4(4) |
| O1^#2^ | Cu1 | O3^#1^ | 178.5(3) | O3^#5^ | Cu3 | O7 | 113.0(2) |
| O1 | Cu1 | O3^#1^ | 83.6(3) | O3^#4^ | Cu3 | O7 | 83.2(17) |
| O1^#2^ | Cu1 | O1 | 95.7(4) | O3^#3^ | Cu3 | O7 | 107.6(17) |
| O1 | Cu1 | N3 | 97.5(6) | O3 | Cu3 | O7 | 78.0(2) |
| O1^#2^ | Cu1 | N3 | 98.5(6) | O7 | Cu3 | Cu2 | 107.0(2) |
| O3^#3^ | Cu2 | O3 | 77.5(4) | O3 | Cu3 | O3^#4^ | 98.4(4) |

Symmetry codes: #1: +Y, +X, +Z; #2: +Y, 1‒X,+Z; #3: +X, 1‒Y, +Z; #4: +X, +Y, 1‒Z; #5: +X, 1‒Y, 1‒Z.

1. **Supporting Figures.**


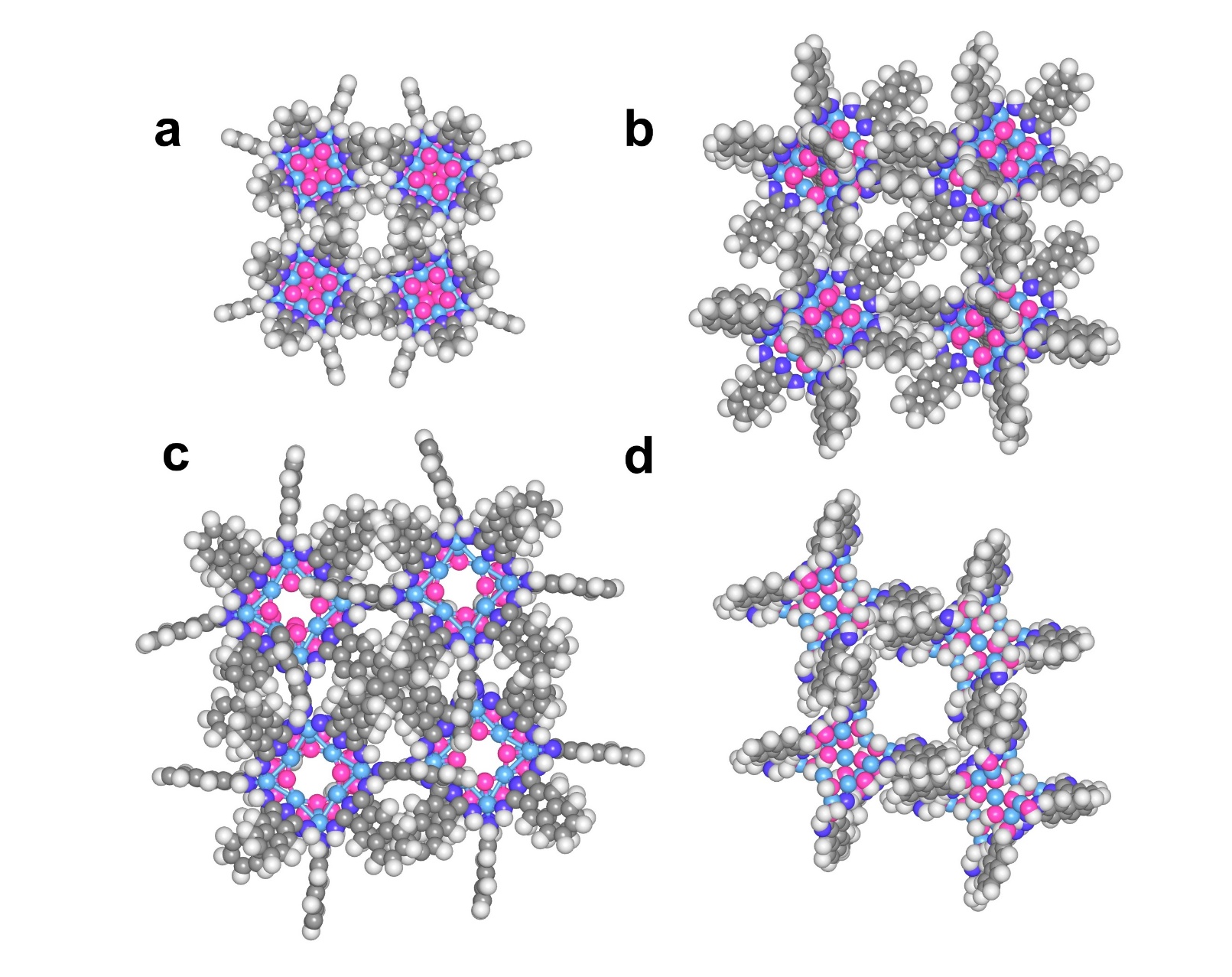


**Figure S1.** Pore structures of **3** (a), **1** (b), activated **1** (c), and **2** (d). All atoms are represented in the space-filling model.

**
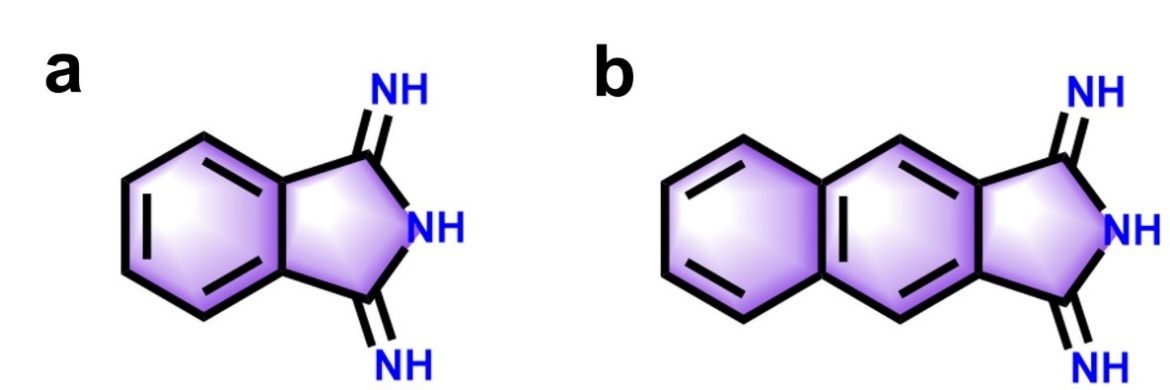
**

**Figure S2.** The structures of ligand HL' (a) and HL (b).

**
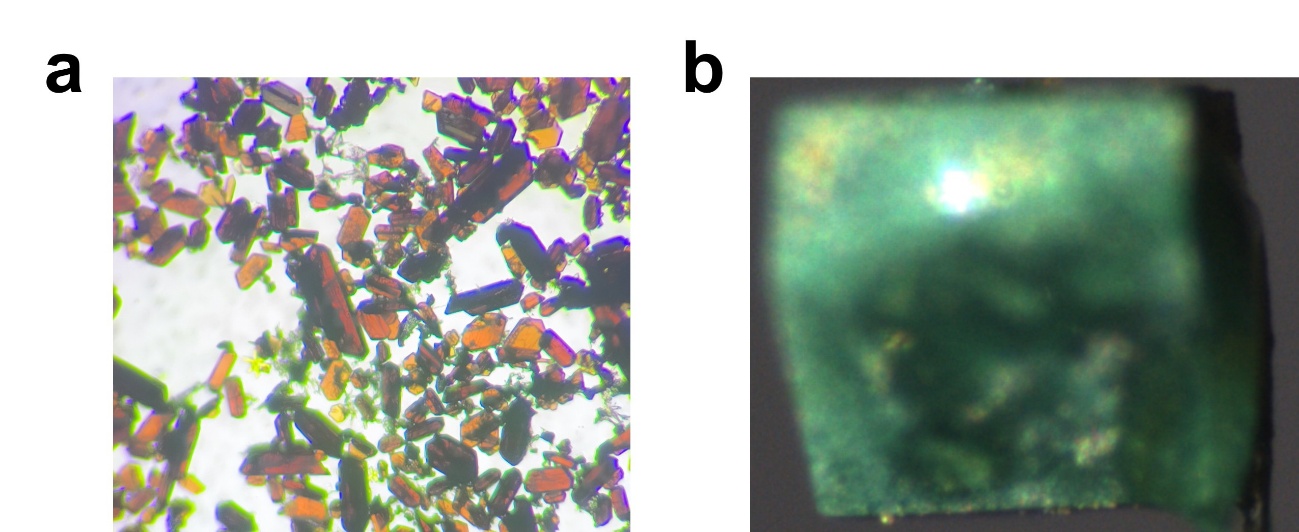
**

**Figure S3.** Optical photographs of crystals of **1** (a) and **2** (b).


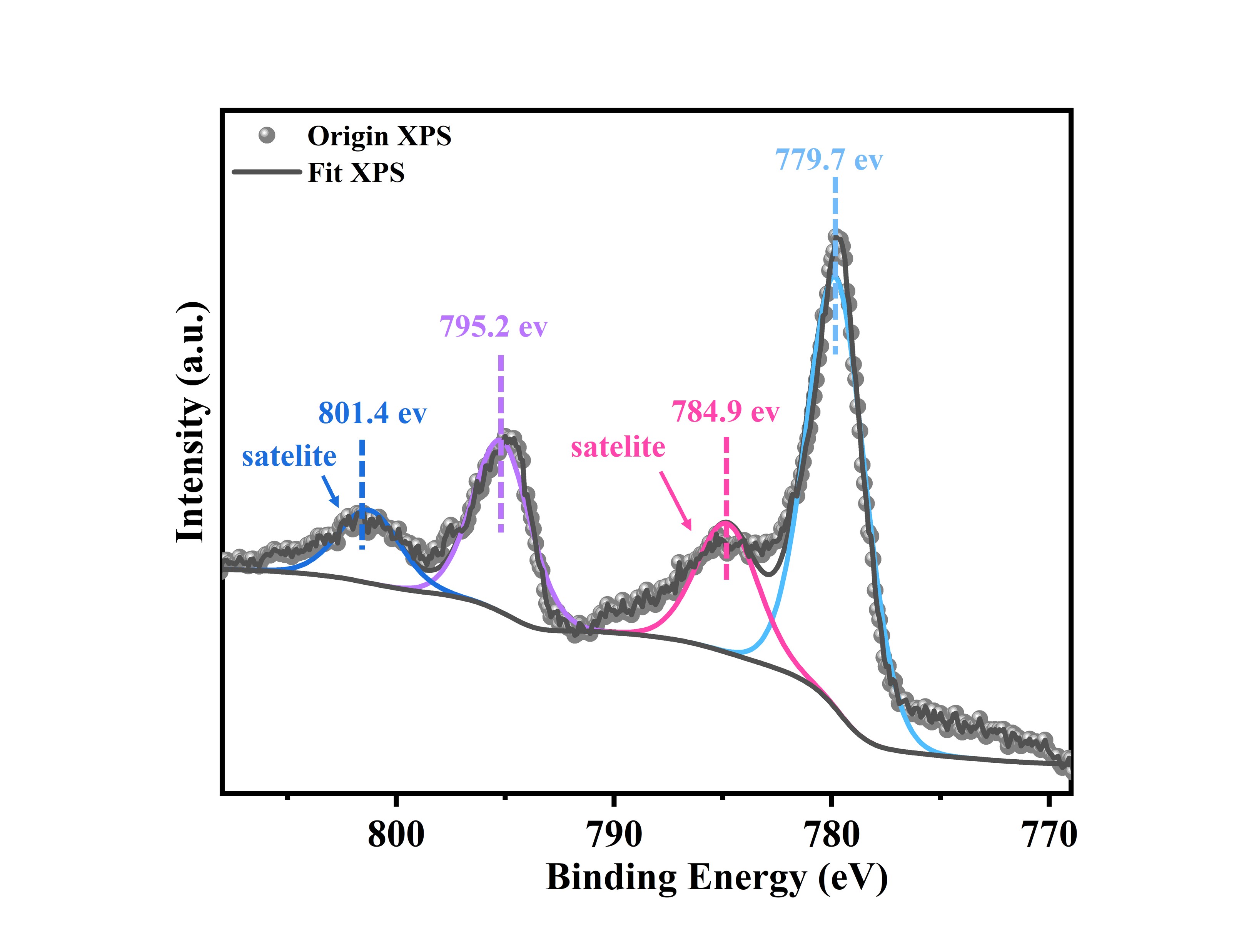


**Figure S4.** XPS spectrum of compound **1**. The Co2*p* spectrum consists of peaks at 779.7 and 795.2 eV corresponding to the Co^2+^ and Co^3+^. The satellite peaks at 784.9 eV and 801.4 eV indicates that Co^2+^ and Co^3+^ coexist in compound **1**.^[14]^


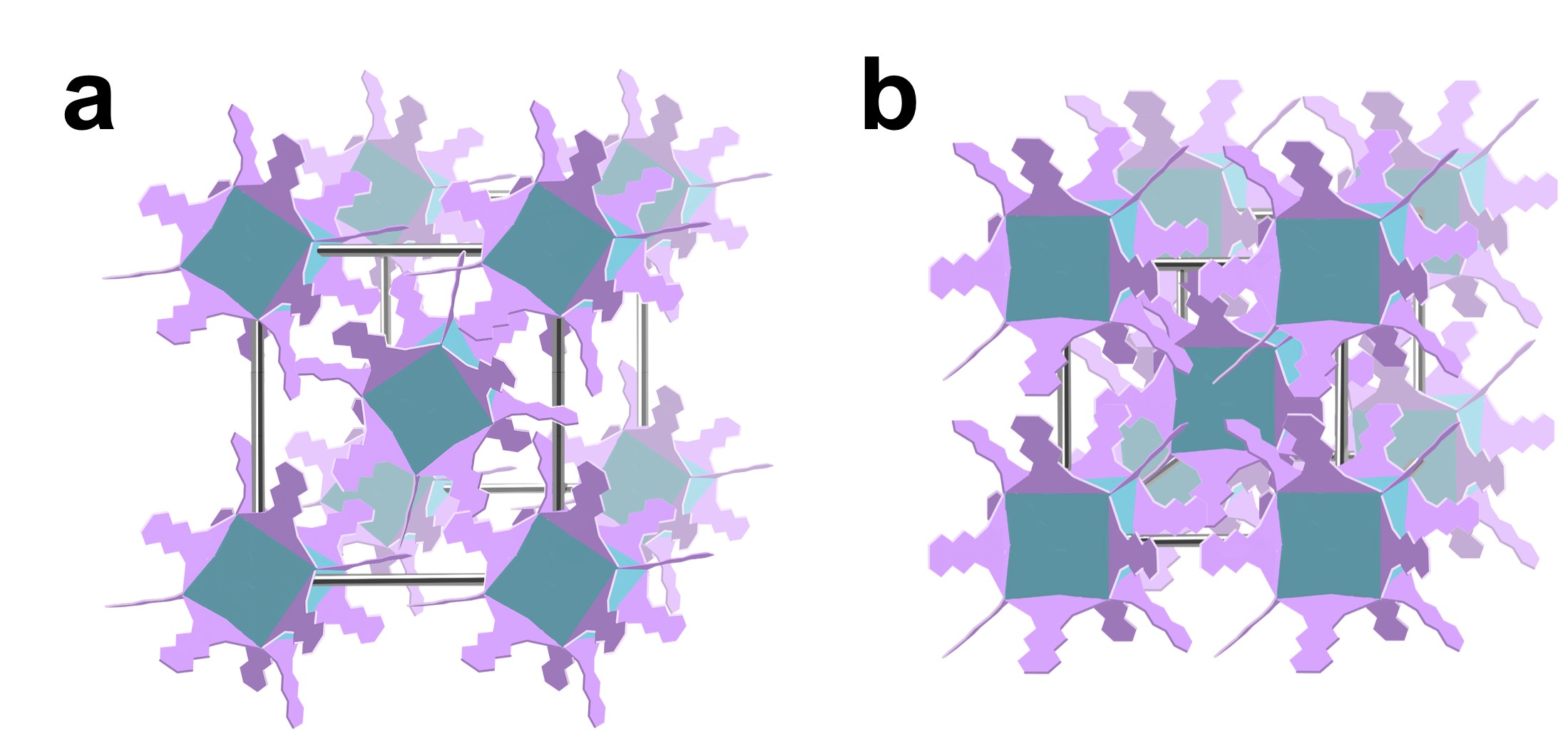


**Figure S5.** Packings of the building units in the lattices of **1** (a) and activated **1** (b).

**
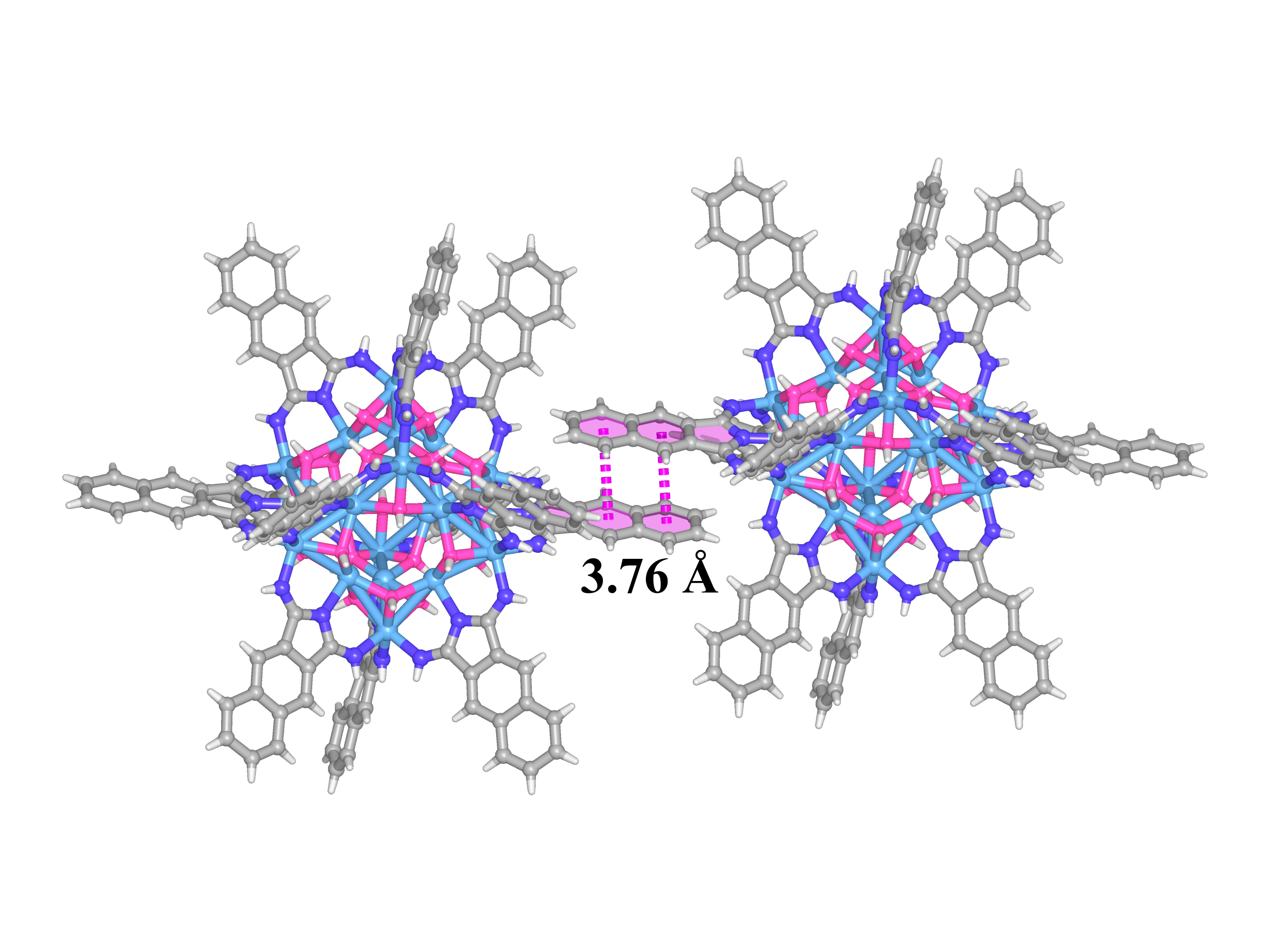
**

**Figure S6.** Noncovalent intermolecular interaction for compound **1**. (The π···π interaction is indicated by red dotted lines). Atom color: blue, Co; purple, N; pink, O; grey, C; white, H.


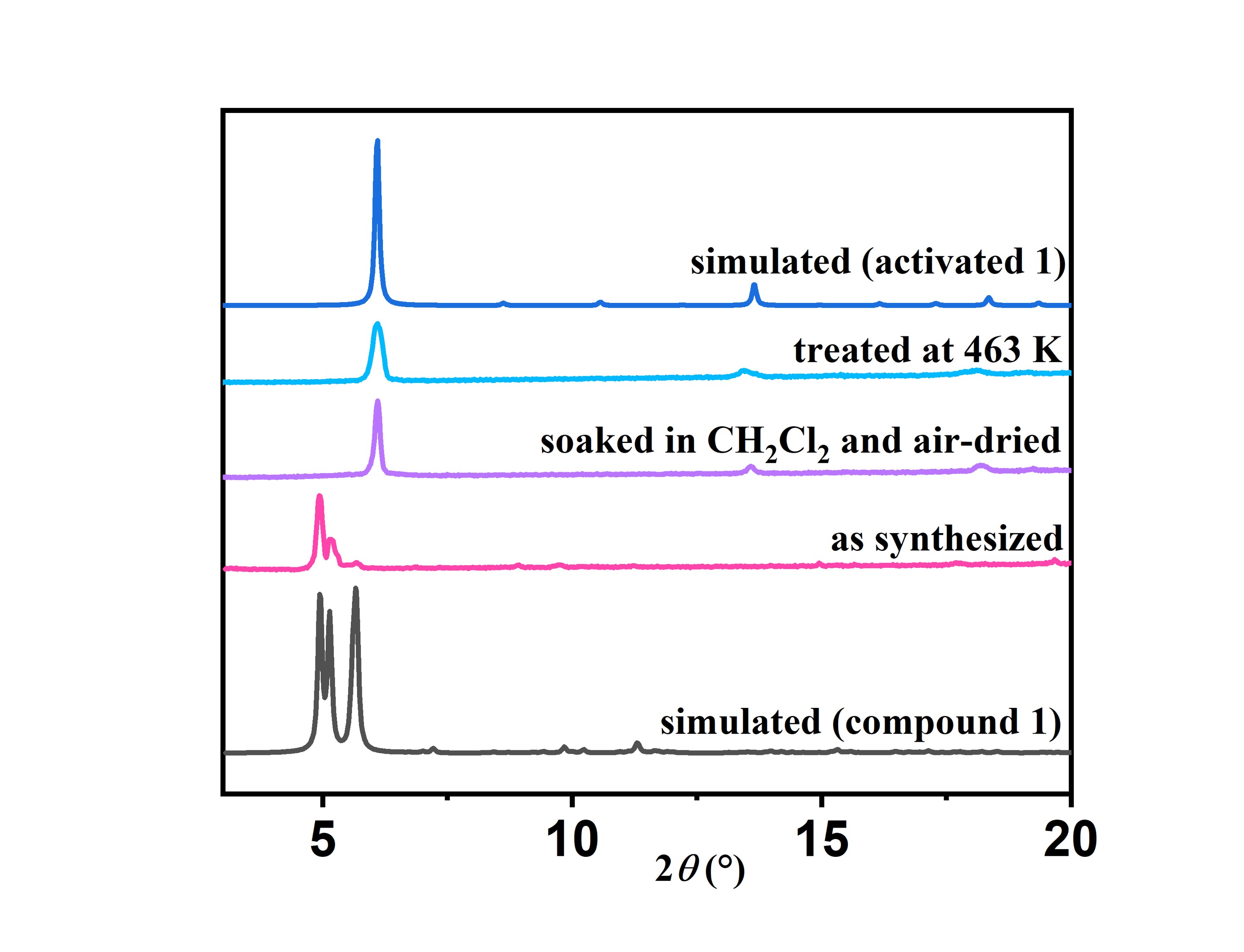


**Figure S7.** PXRD patterns showing the transformation from compound **1** to activated **1** upon desolvation.

**
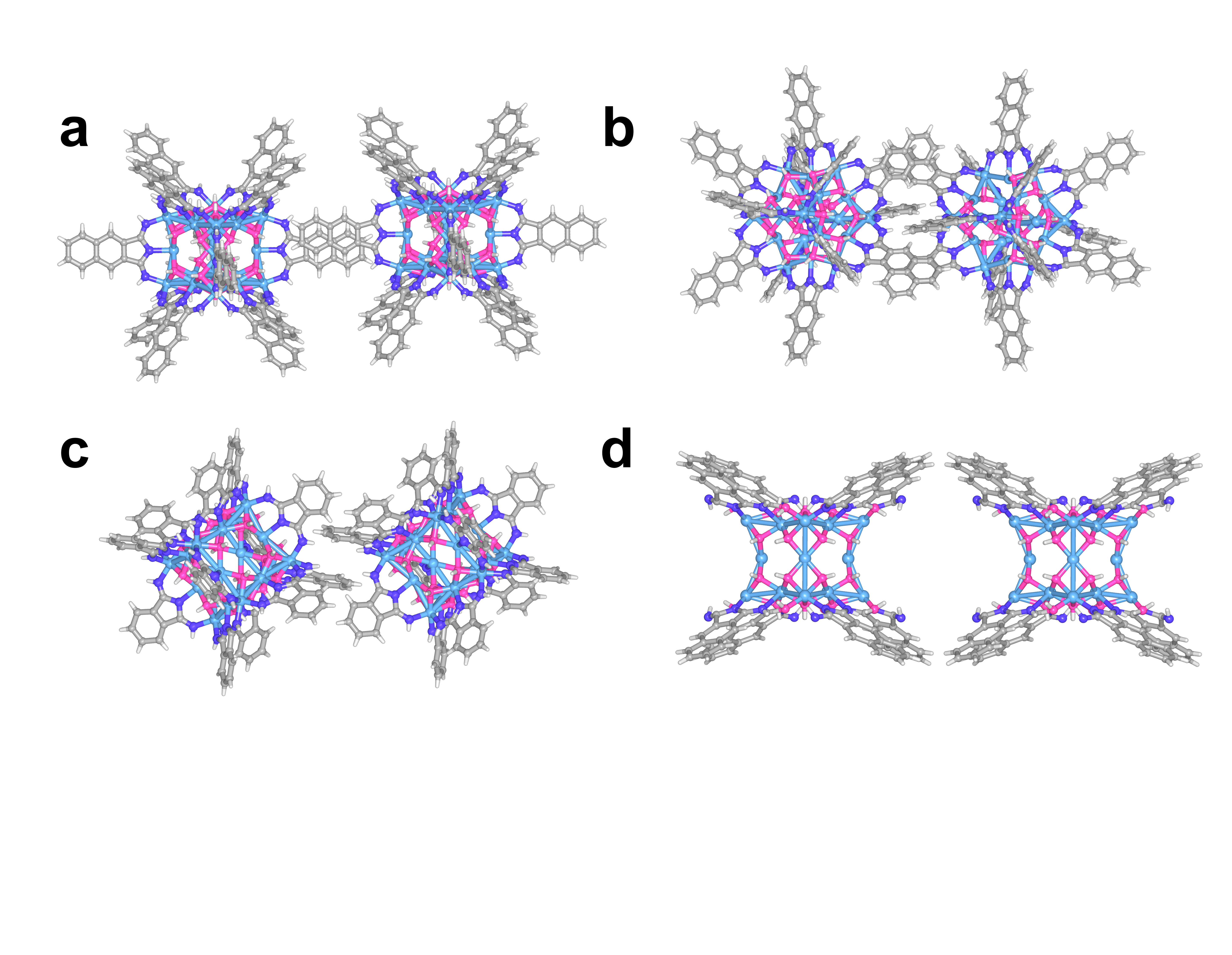
**

**Figure S8.** (a)‒(d) Nearest intercluster packings of **1**, activated **1, 3**, and **2**, respectively.


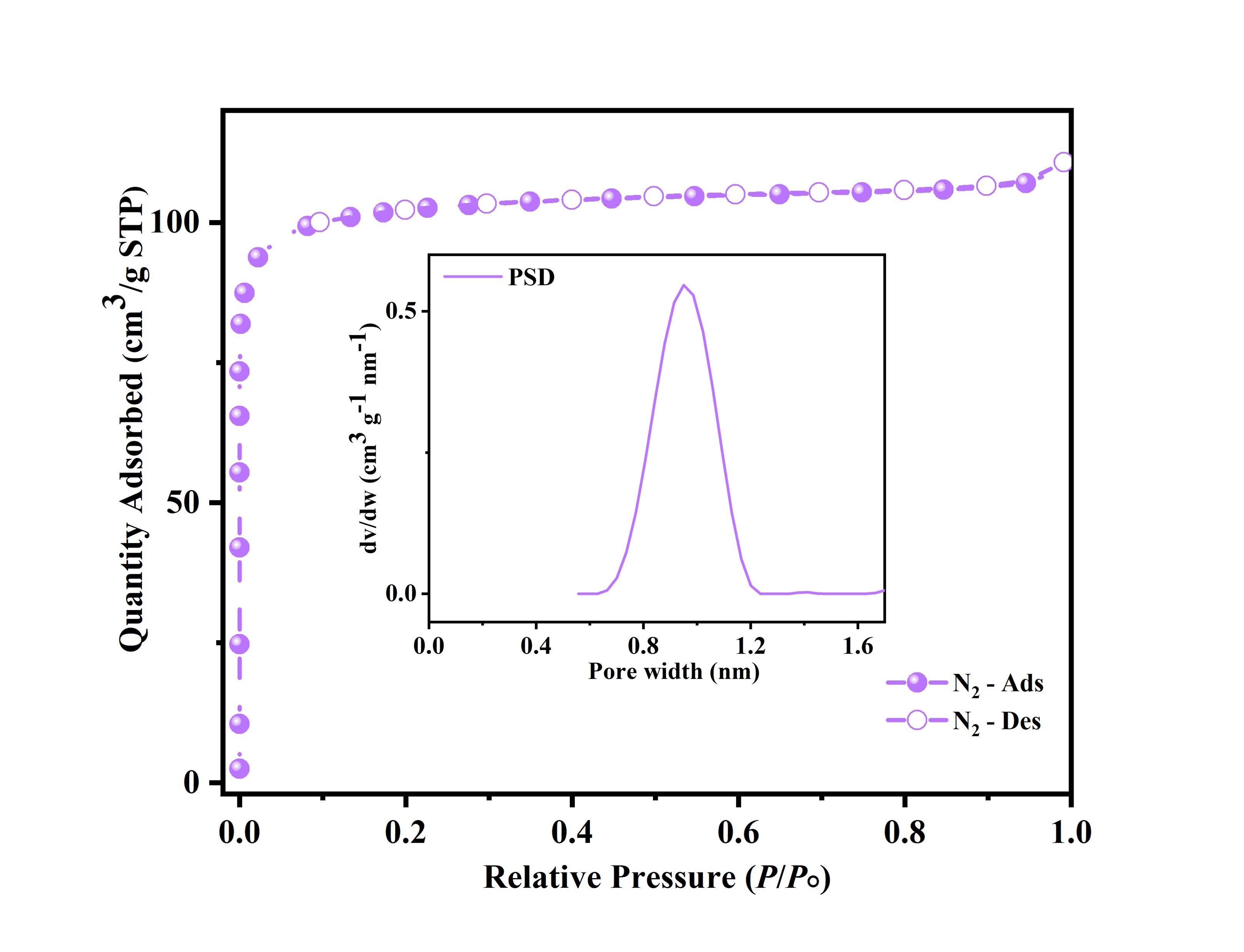
**Figure S9.** Nitrogen sorption isotherm of activated **1** at 77 K (filled symbols: adsorption; open symbols: desorption). The inset graph shows the pore size distribution (PSD) analyzed by NLDFT method.


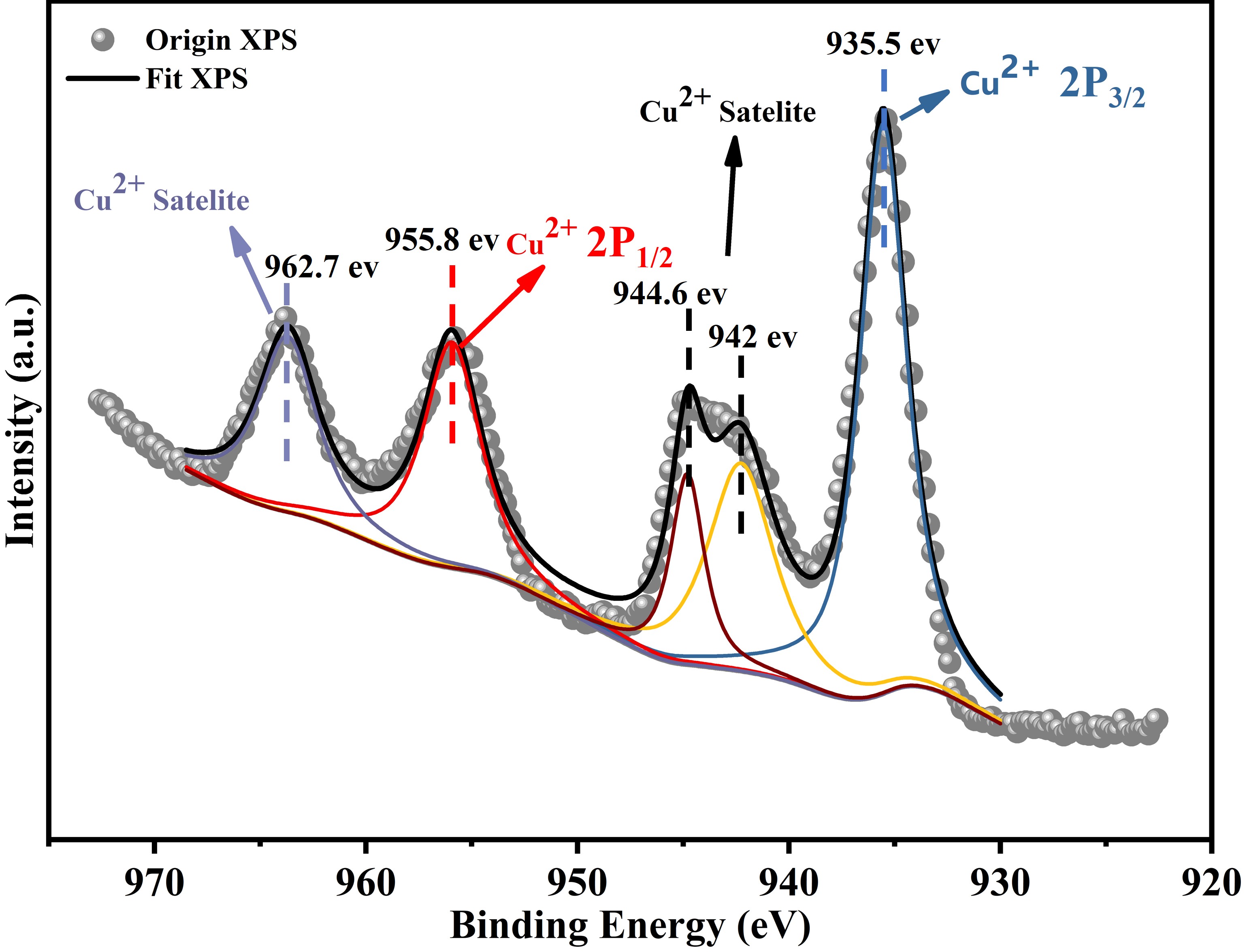


**Figure S10.** XPS spectrum of compound **2**. XPS in the Cu 2*P*_3/2_ and Cu 2*P*_1/2_ region recorded two main peaks at ~935.5 eV and ~955.8 eV, respectively, as well as three satellite peaks at 942 eV, 944.6 eV and 962.7 eV. All features are characteristic of the Cu (II) oxidation state.^[15,16]^


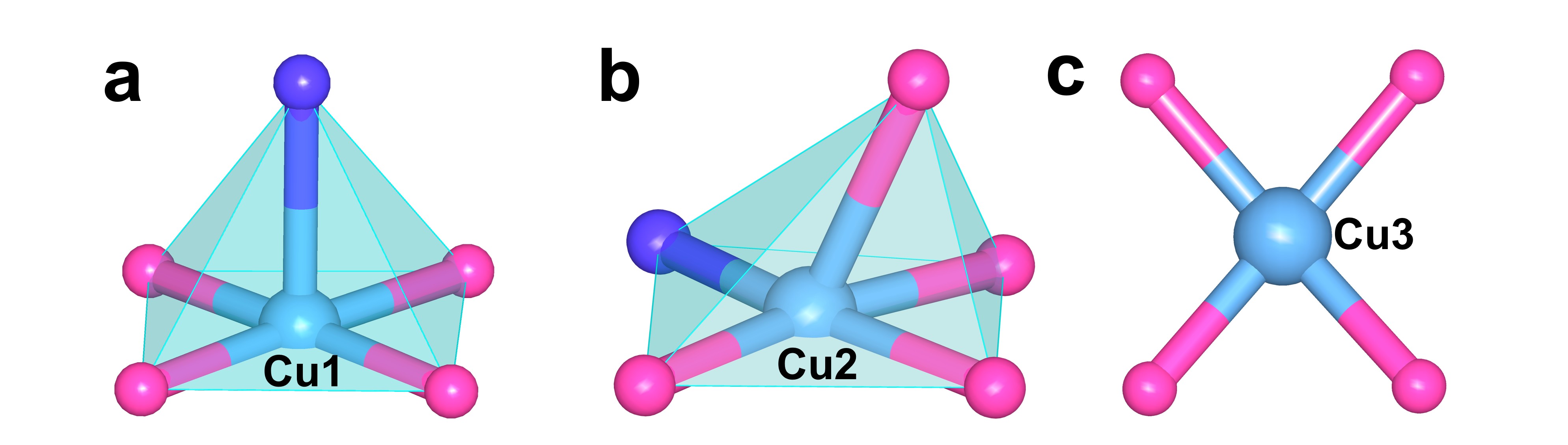


**Figure** **S11.** Coordination geometries of the three crystallographically independent Cu^2+^ in compound **2**. Atom colours: blue, Cu; purple, N; pink, O.


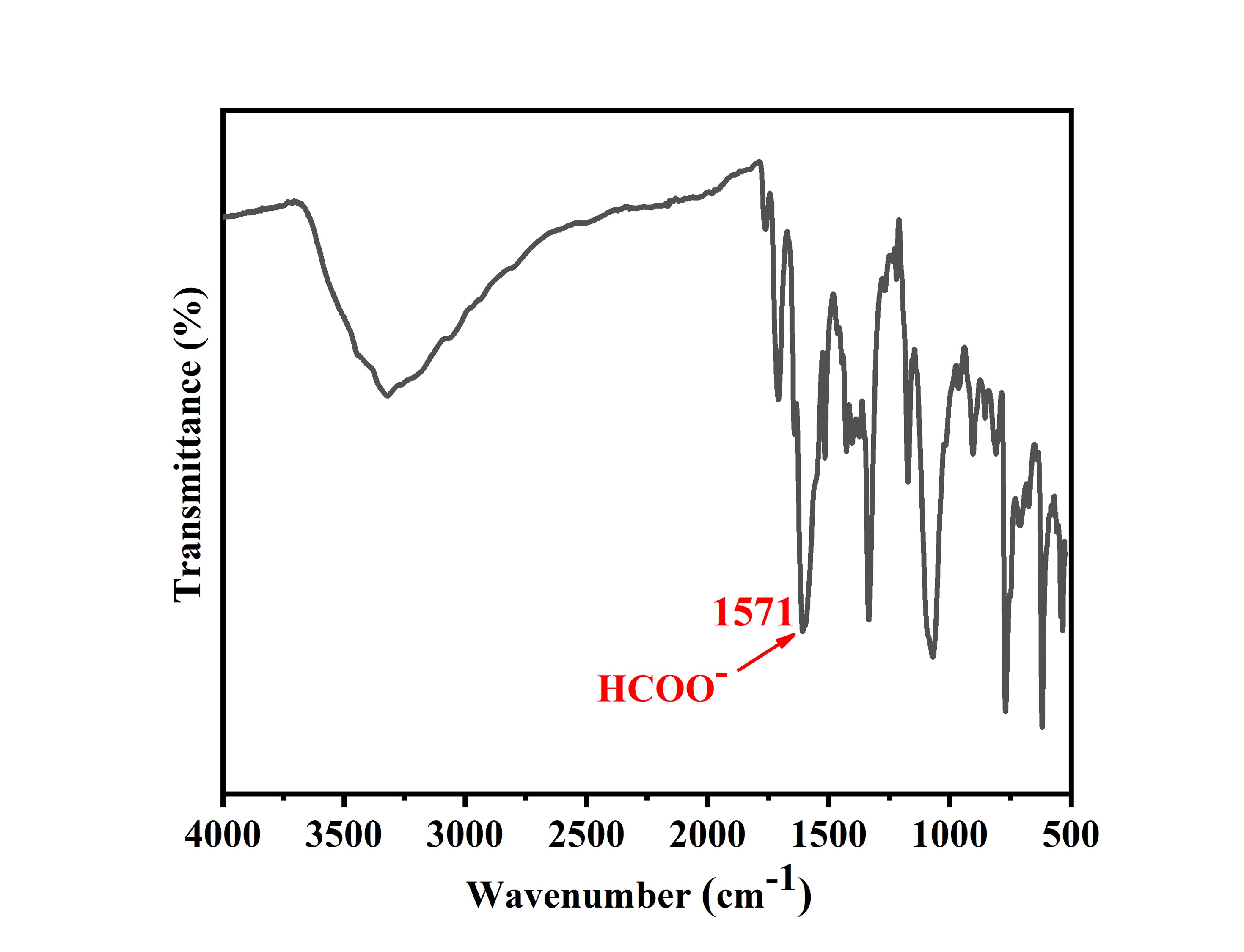


**Figure S12.** IR spectrum of compound **2**. The absorption at 1571 cm^–1^ can be assigned to the *ʋ*_C=O_ from HCOO^–^, which is the product from DMF hydrolysis.


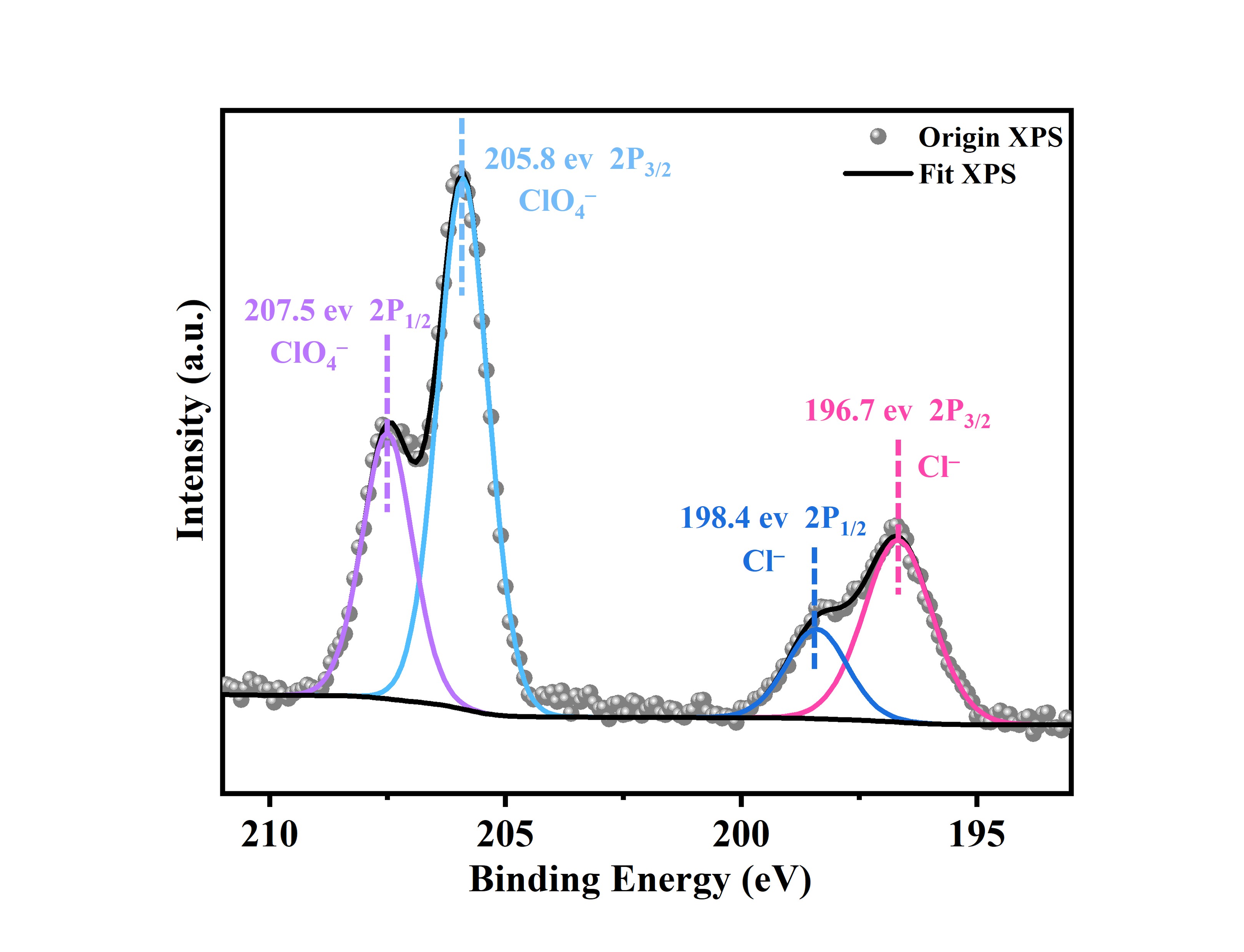


**Figure S13.** XPS spectrum of compound **2**. The Cl2*p* XPS spectrum displays two distinct doublets: a lower-energy pair at 196.7 eV (2*P*_3/2_) and 198.4 eV (2*P*_1/2_) corresponding to Cl^‒^, and a higher-energy pair at 205.8 eV (2*P*_3/2_) and 207.5 eV (2*P*_1/2_) characteristic of ClO_4_^‒^.^[17]^


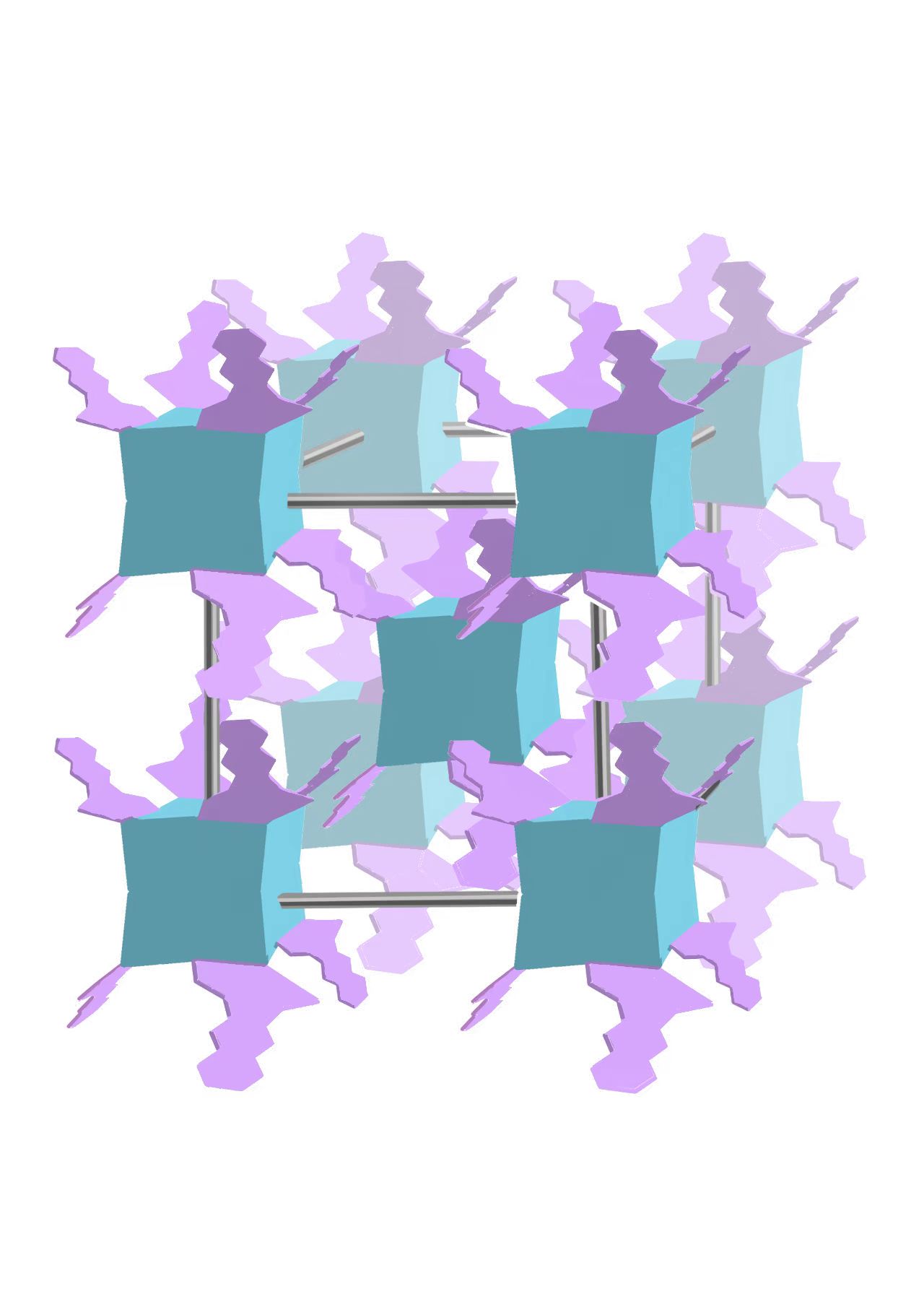


**Figure S14.** Packing of the building units in the lattice of compound **2**.


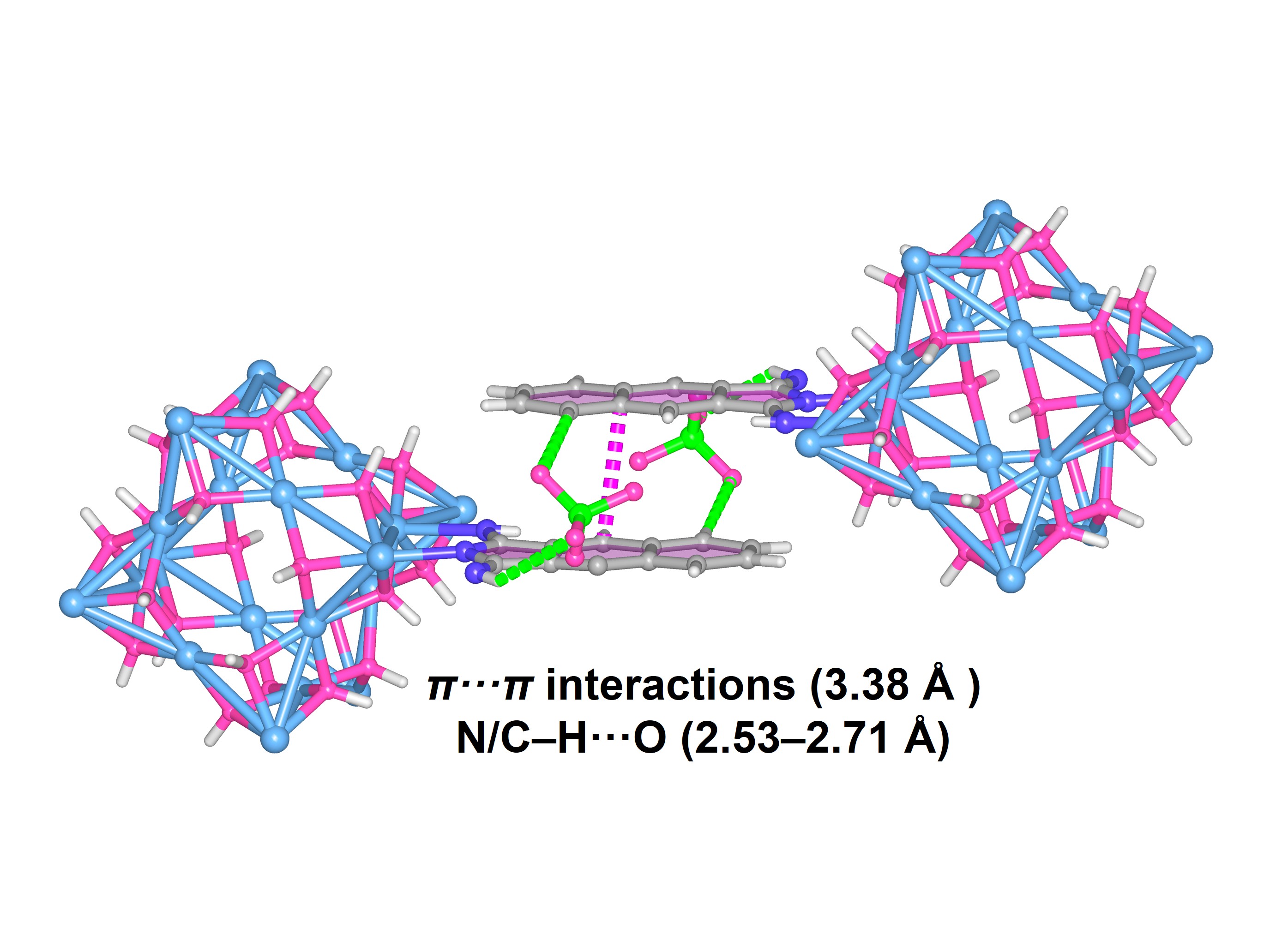


**Figure S15.** Noncovalent intermolecular interactions for compound **2**. (The *π···π* interactions and hydrogen bonds are indicated by red dotted lines and green dotted lines, respectively). Atom color: blue, Cu; purple, N; pink, O; grey, C; white, H. Some ligands have been omitted for clarity.


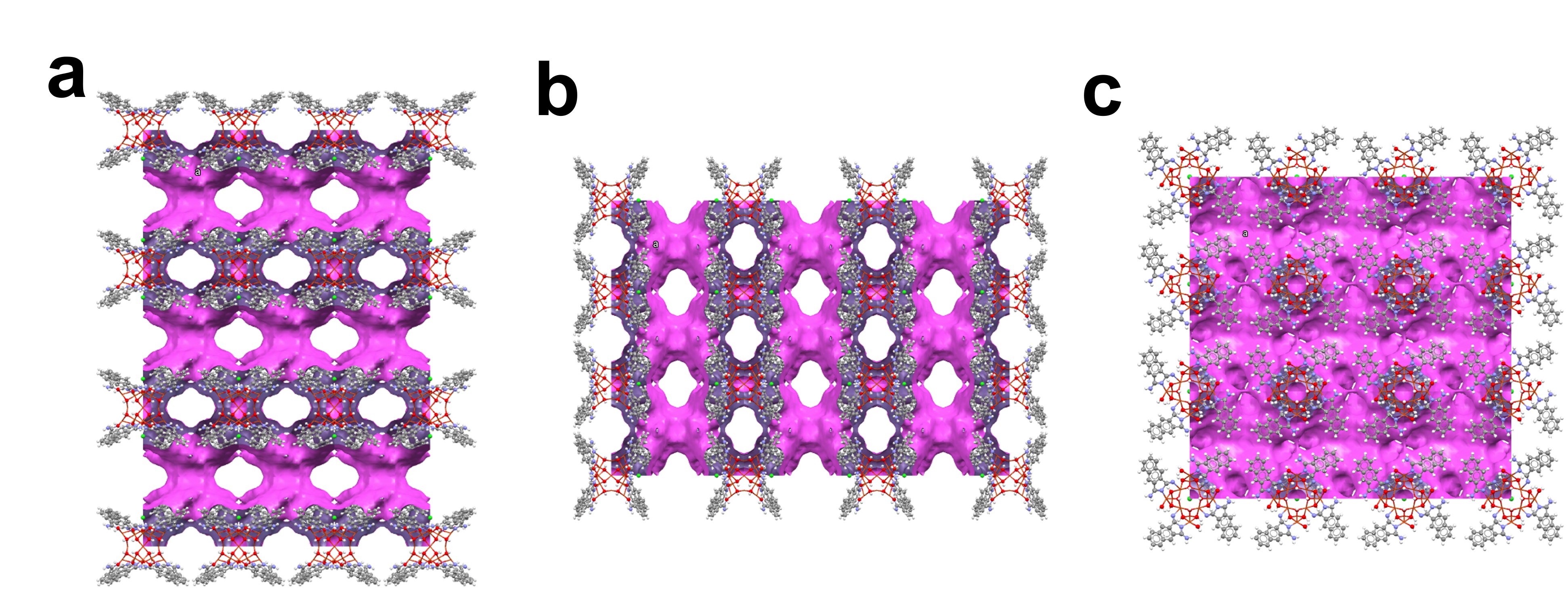


**Figure S16.** Supramolecular porous framework structure for compound **2**. (a)‒(c) Schematic diagrams of the pore channels of compound **2** along *a*, *b*, and *c* axis, respectively. Pore surface is indicated as purple curved surface.


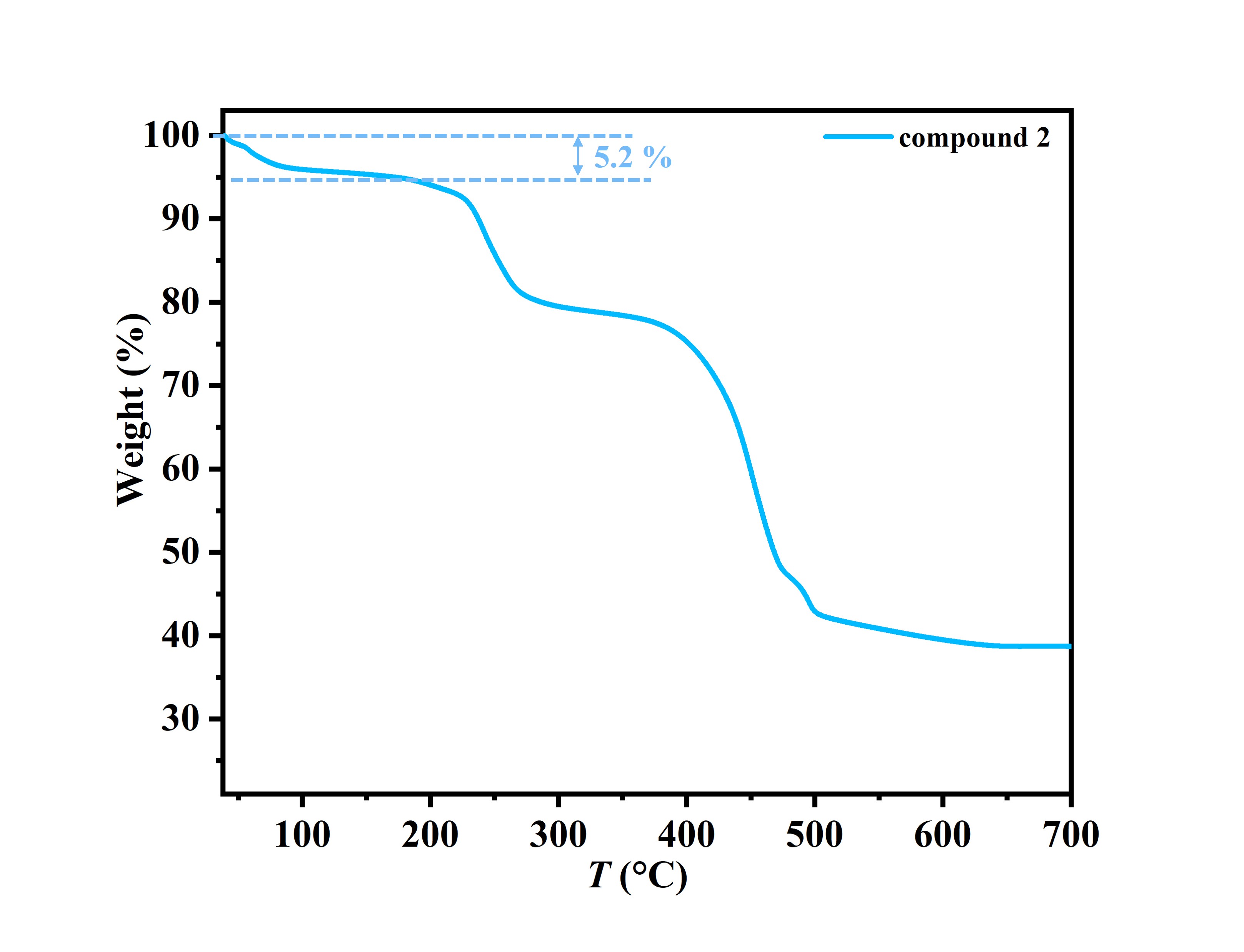


**Figure S17.** TGA plot for compound **2**.


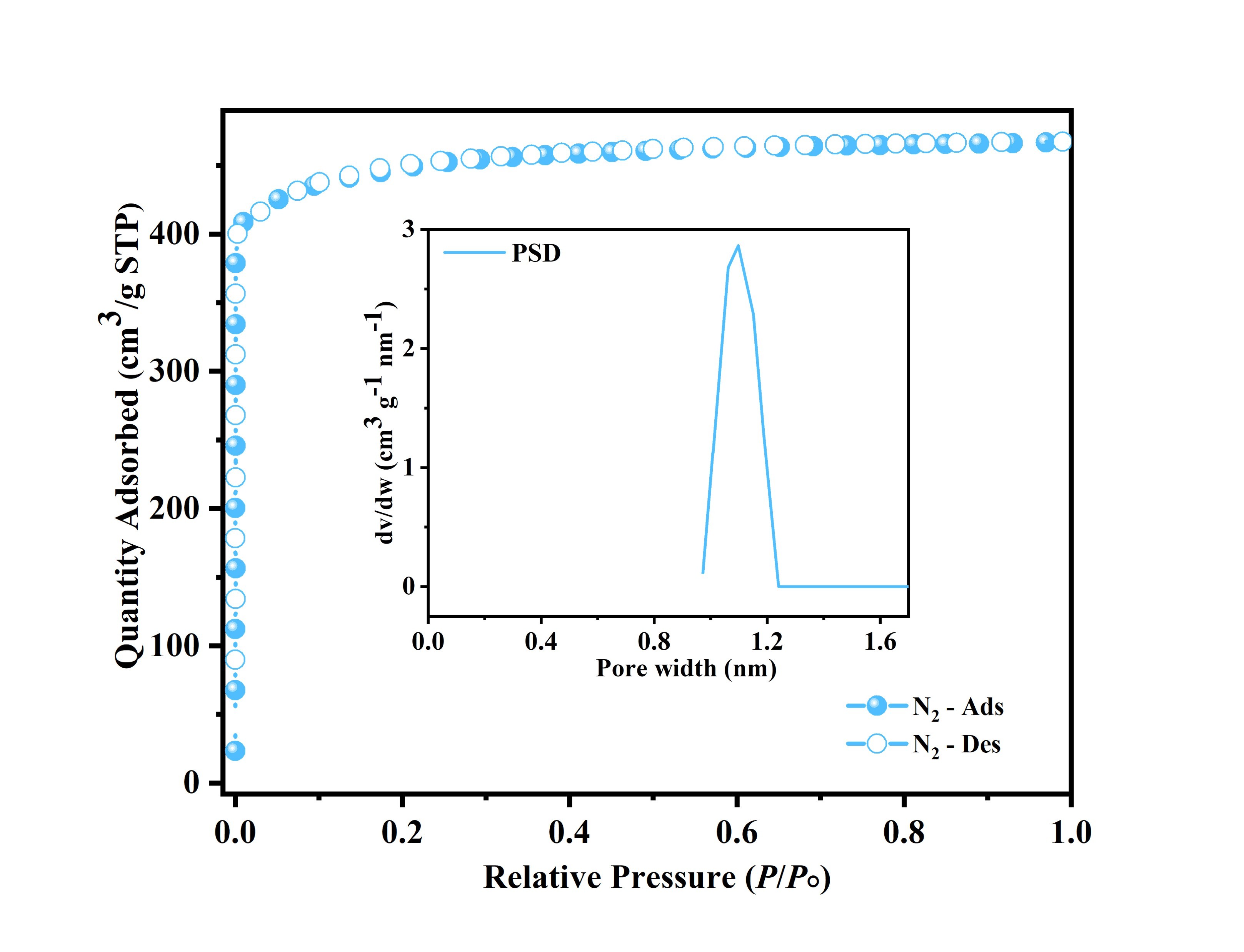


**Figure S18.** Nitrogen sorption isotherm of compound **2** at 77 K (filled symbols: adsorption; open symbols: desorption). The inset graph shows the pore size distribution (PSD) analyzed by NLDFT method.

**
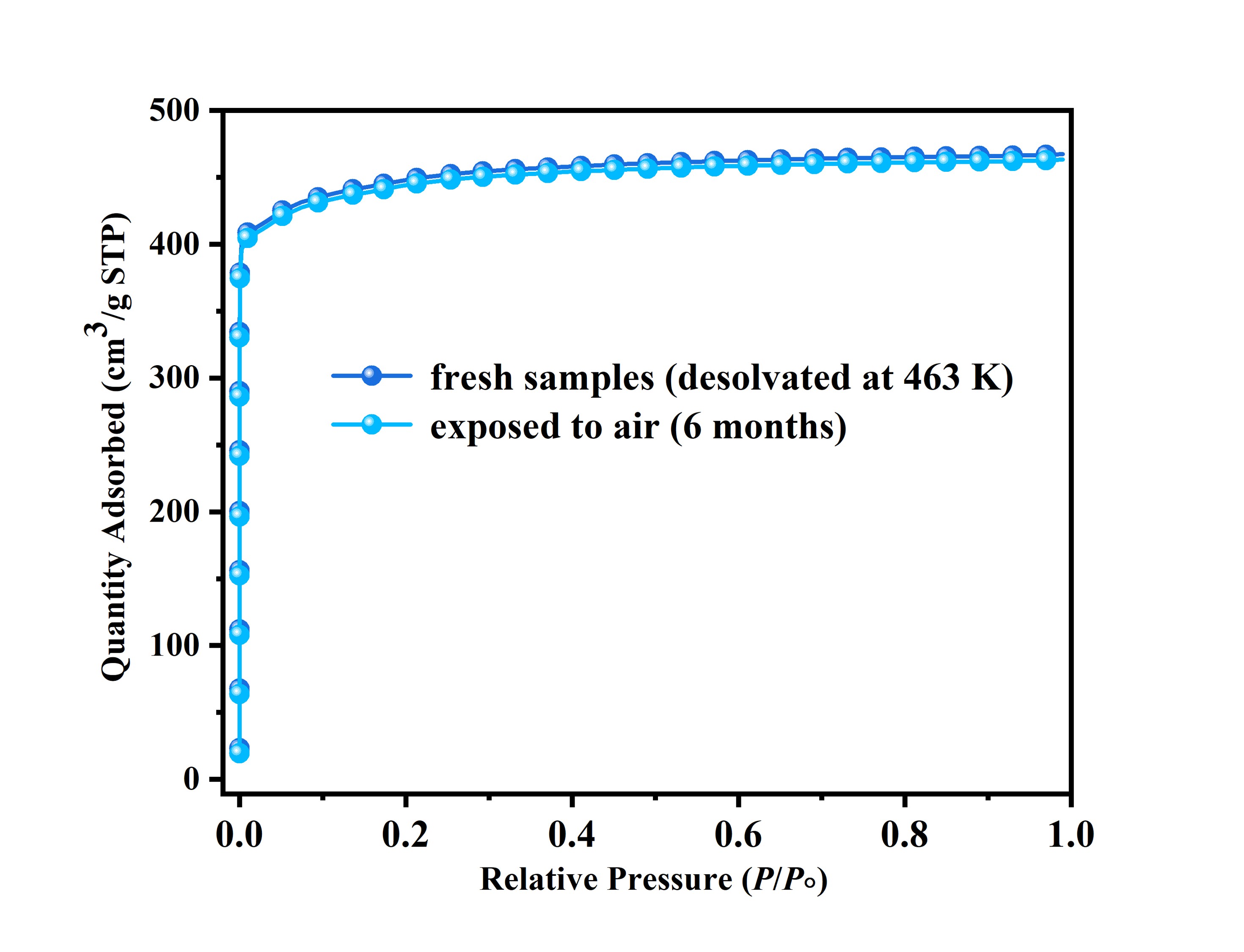
**

**Figure S19.** Nitrogen sorption isotherms at 77 K for fresh compound **2** and compound **2** after being stored at ambient condition for six months.


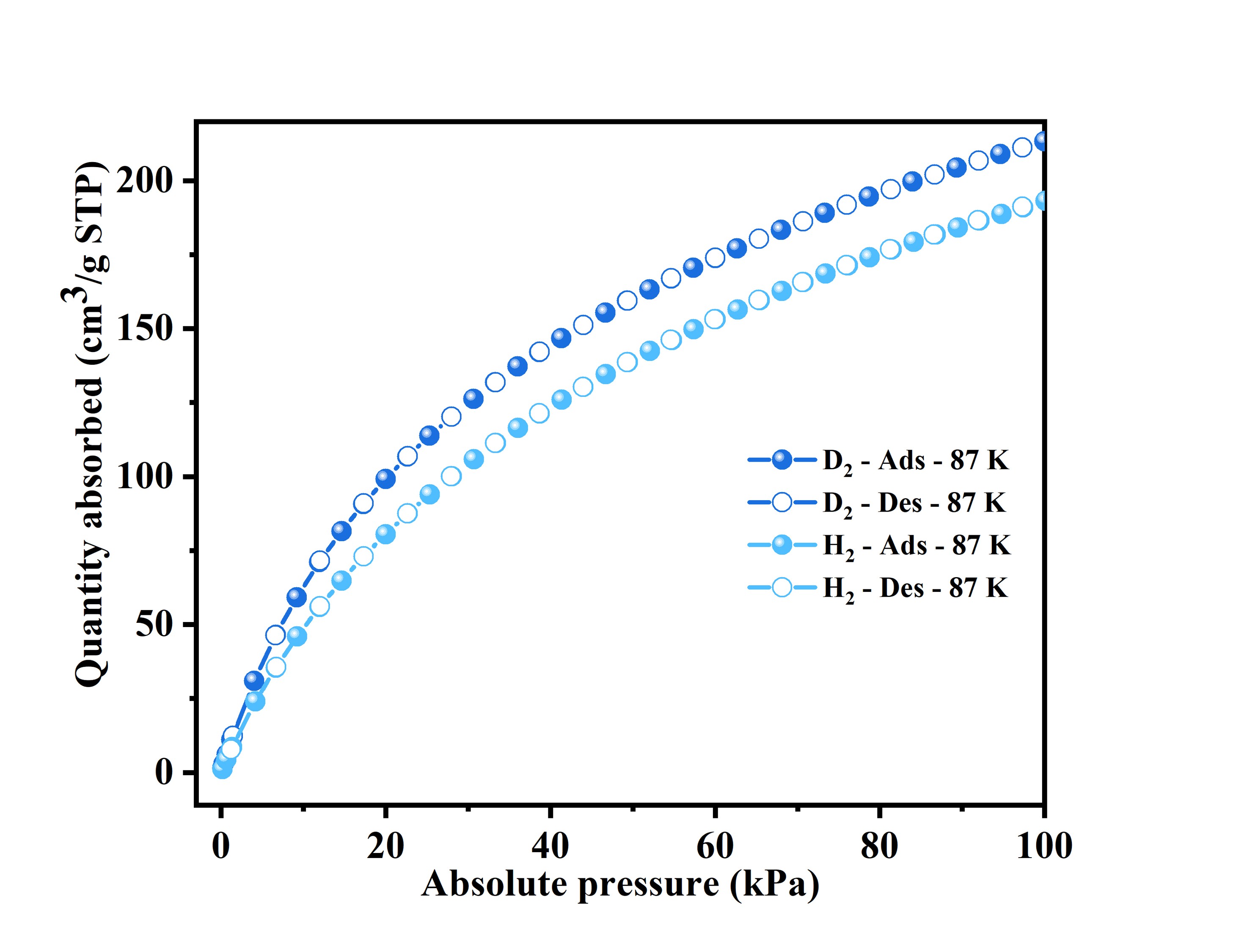


**Figure S20.** D_2_ and H_2_ sorption isotherms of activated **2** at 87 K and 100 kPa.


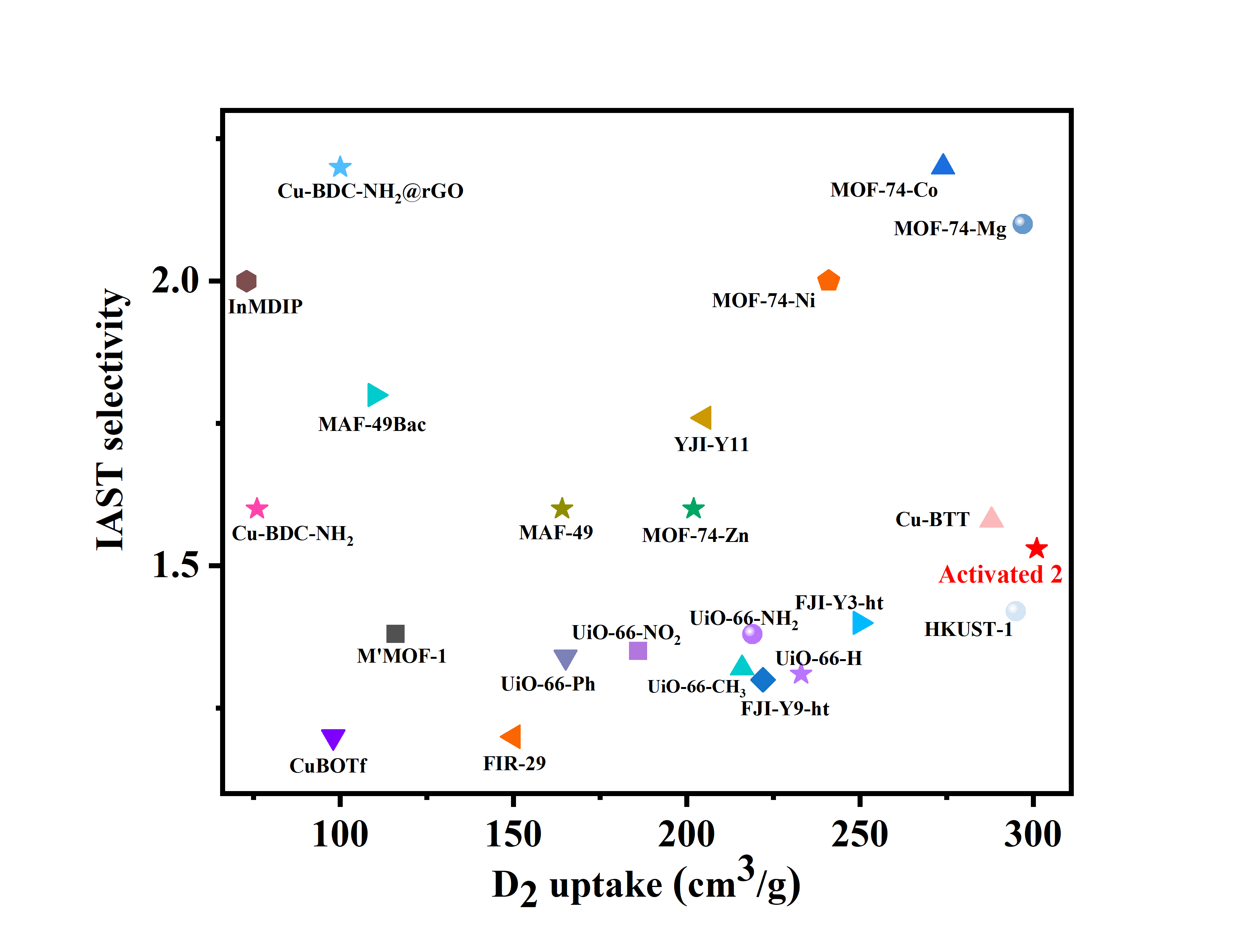


**Figure S21.** Comparison of activated **2** with various other adsorbents on the hydrogen isotope separation at 77 K and 100 kPa.


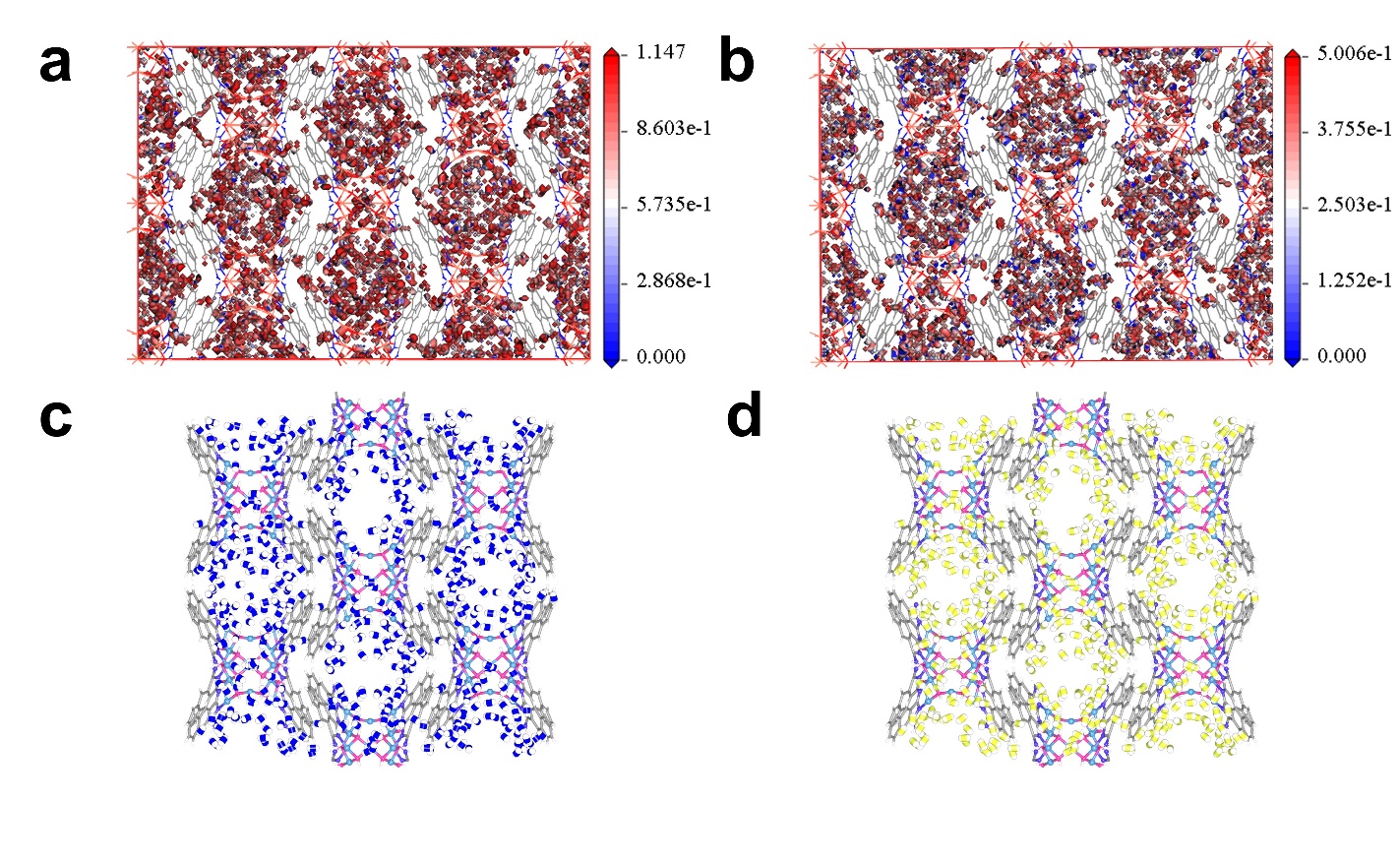


**Figure S22.** Adsorption density view of adsorbed D_2_ (a) and H_2_ (b) in the pores of activated **2** at 77 K. The simulated D_2_ (c) and H_2_ (d) adsorption sites in the pores of activated **2**.

1. **Supporting References.**
2. Y. -J. Guo, K. -Z. Su, W. -J. Wang, D. -Q. Yuan, *Inorg. Chem*. **2025**, *64*, 18599.
3. Y. -N. Si, W. -J. Wang, E. M. El-Sayed, D. -Q. Yuan, *Sci. China Chem*. **2020**, *63*, 881.
4. Y. -N. Si, X. He, J. Jiang, Z. -M. Duan, W. -J. Wang, D. -Q. Yuan, *Nano Res*. **2021**, *14*, 518.
5. Y.-P Huang, Z.-F. Ju, K.-Z. Su, W.-J. Wang, D.-Q Yuan, *Inorg. Chem*. **2025**, *64*, 1203.
6. L. -Q. Li, C. -Q. Ji, W. -J. Wang, F. Wu, Y. -X. Tan, D. -Q. Yuan, *Inorg. Chem. Front.* **2022**, *9*, 1674.
7. Y. -X. Tan, Y. -N. Si, W. -J. Wang, D. -Q. Yuan, *J. Mater. Chem. A*. **2017**, *5*, 23276.
8. S. A. FitzGerald, C. J. Pierce, J. L. C. Rowsell, E. D. Bloch, J. A. Mason, *J. Am. Chem. Soc*. **2013**, *135*, 9458.
9. X.-F. Li, Y.-X. Tan, Z.-F. Ju, W.-J. Wang, D.-Q. Yuan, *Inorg. Chem. Front*. **2025**, *12*, 701.
10. F. -J. Zhao, Y. -X. Tan, W. -J. Wang, Z. -F. Ju, D. -Q. Yuan, *Inorg. Chem*. **2018**, *57*, 13312.
11. B. -L. Chen, X. -B. Zhao, A. Putkham, K. -L. Hong, E. B. Lobkovsky, E. J. Hurtado, A. J. Fletcher, K. M. Thomas, *J. Am. Chem. Soc*. **2008**, *130*, 6411.
12. A. Dastbaz, J. Karimi-Sabet, Y. Amini, M. A. Moosavian, *Int. J. Hydrogen Energy*. **2024**, *61*, 893.
13. D. Noguchi, H. Tanaka, A. Kondo, H. Kajiro, H. Noguchi, T. Ohba, H. Kanoh, K. Kaneko, *J. Am. Chem. Soc.* **2008**, *130*, 6367.
14. Q. -Q. Yan, J. Wang, L. -D. Zhang, J. -Q. Liu, M. Wahiduzzaman, N. -A. Yan, L. Yu, R. Dupuis, H. Wang, G. Maurin, M. Hirscher, P. Guo, S. -J. Wang, J. -F. Du, *Nat. Commun*. **2023**, *14*, 4189.
15. J. Iqbal, A. Numan, M. O. Ansari, R. Jafer, P. R. Jagadish, S. Bashir, P. M. Z. Hasan, A. L. Bilgrami, S. Mohamad, K. Ramesh, S. Ramesh, *Polymers* **2020**, *12*, 2816.
16. Z. -W. Wu, X. Zhang, E. D. Goodman, W. -X. Huang, A. R. Riscoe, S. Yacob, M. Cargnello, *ACS Catal*. **2020**, *10*, 9356.
17. F. C. De Godoi, E. Rodriguez-Castellon, E. Guibal, M. M. Beppu, *Chem. Eng. J*. **2013**, *234*, 423.
18. Y. -S. Li, L. -S. Geng, B. -M. Zhang, Z. -H. Wei, H. Fan, J. -H. Li, W. -C. Feng, L. Zhou, *Rare Met*. **2025**, *44*, 950.
