# Supplementary material for: Engineering Active Metal and Nonmetal Sites in Porous Structures of Metal‐Hydroxide Clusters for Enhanced D2/H2 Uptake and Separation
Source: Adv Sci (Weinh). 2025 Dec 14;13(13):e19498. doi: 10.1002/advs.202519498 (PMC12955987; doi:10.1002/advs.202519498)

checkCIF/PLATON report

Structure factors have been supplied for datablock(s) wwlnjcubic_auto

THIS REPORT IS FOR GUIDANCE ONLY. IF USED AS PART OF A REVIEW PROCEDURE FOR PUBLICATION, IT SHOULD NOT REPLACE THE EXPERTISE OF AN EXPERIENCED CRYSTALLOGRAPHIC REFEREE.

No syntax errors found.   CIF dictionary   Interpreting this report

Datablock: wwlnjcubic_auto

Bond precision: Co- O = 0.0229 A   Wavelength=0.71073

Cell:	a=20.7655(10)	b=20.7655(10)	c=20.7655(10)
	alpha=90	beta=90	gamma=90
Temperature:	300 K		

	Calculated	Reported
Volume	8954.2(13)	8954.2(13)
Space group	P -4 3 m	P -4 3 m
Hall group	P -4 2 3	P -4 2 3
Moiety formula	3(C48 H40 Co6.67 N12.33 O9), C144 H120 Co20 N37 O27 [+ solvent]	C144 H120 Co20 N37 O27, 5[NO3], 6[NO3]
Sum formula	C288 H240 Co40 N74 O54 [+ solvent]	C144 H120 Co20 N48 O60
Mr	7958.84	4661.47
Dx,g cm-3	1.476	1.729
Z	1	2
Mu (mm-1)	1.866	1.895
F000	3998.0	4680.0
F000'	4013.08	
h,k,lmax	27,27,27	21,27,26
Nref	4091[2206]	2914
Tmin,Tmax	0.545,0.871	0.072,1.000
Tmin'	0.464	

Correction method= # Reported T Limits: Tmin=0.072 Tmax=1.000
AbsCorr = MULTI-SCAN

Data completeness= 1.32/0.71   Theta(max)= 28.110

```
R(reflections)= 0.1459( 1056)                          wR2(reflections)=
                                                       0.5178( 2914)
S = 1.054                      Npar= 352
```

The following ALERTS were generated. Each ALERT has the format
test-name_ALERT_alert-type_alert-level.
Click on the hyperlinks for more details of the test.

Alert level A

```
PLAT084_ALERT_3_A High wR2 Value (i.e. > 0.25) ..................       0.52 Report
```

Author Response: There are serious disordered structures in the crystals at high temperature.

Alert level B

```
PLAT026_ALERT_3_B Ratio Observed / Unique Reflections (too) Low ..        36% Check
PLAT090_ALERT_3_B Poor Data / Parameter Ratio (Zmax > 18) ........      5.38 Note
PLAT220_ALERT_2_B NonSolvent   Resd 1  N   Ueq(max)/Ueq(min) Range      10.0 Ratio
PLAT220_ALERT_2_B NonSolvent   Resd 1  O   Ueq(max)/Ueq(min) Range      10.0 Ratio
PLAT220_ALERT_2_B NonSolvent   Resd 2  N   Ueq(max)/Ueq(min) Range      10.0 Ratio
PLAT220_ALERT_2_B NonSolvent   Resd 2  O   Ueq(max)/Ueq(min) Range      10.0 Ratio
PLAT420_ALERT_2_B D-H Bond Without Acceptor  O2       --H2A      .    Please Check
PLAT420_ALERT_2_B D-H Bond Without Acceptor  O5       --H5       .    Please Check
PLAT420_ALERT_2_B D-H Bond Without Acceptor  O27A     --H27A     .    Please Check
```

Alert level C

```
PLAT029_ALERT_3_C _diffrn_measured_fraction_theta_full value Low .      0.976 Why?
PLAT082_ALERT_2_C High R1 Value ..................................       0.15 Report
PLAT242_ALERT_2_C Low    'MainMol' Ueq as Compared to Neighbors of        O5 Check
PLAT242_ALERT_2_C Low    'MainMol' Ueq as Compared to Neighbors of       O14 Check
PLAT250_ALERT_2_C Large U3/U1 Ratio for <U(i,j)> Tensor(Resd    1)       2.4 Note
PLAT250_ALERT_2_C Large U3/U1 Ratio for <U(i,j)> Tensor(Resd    2)       2.9 Note
PLAT260_ALERT_2_C Large Average Ueq of Residue Including       Co2      0.162 Check
PLAT260_ALERT_2_C Large Average Ueq of Residue Including       Co1      0.145 Check
PLAT420_ALERT_2_C D-H Bond Without Acceptor  N2       --H2       .    Please Check
PLAT420_ALERT_2_C D-H Bond Without Acceptor  N5       --H5A      .    Please Check
PLAT420_ALERT_2_C D-H Bond Without Acceptor  N10      --H10A     .    Please Check
PLAT905_ALERT_3_C Negative K value in the Analysis of Variance ...     -6.222 Report
PLAT905_ALERT_3_C Negative K value in the Analysis of Variance ...     -1.528 Report
PLAT911_ALERT_3_C Missing FCF Refl Between Thmin & STh/L=    0.600         37 Report
                3  3  3,    3  3  4,    4  4  4,    4  4  5,    5  5  5,    4  5  6,
                0  0  7,    7  7  7,    4  7  8,    0  0  9,    1  9  9,    9  9  9,
                0  7 10,    3 10 10,    7 10 10,    0  0 11,    4  4 11,    7  7 11,
                3 11 11,    7 11 11,    0  3 12,    0  5 12,    4  5 12,    7  8 12,
                0  0 13,    0  4 13,    4  4 13,    0 10 13,   11 11 13,   12 12 13,
                   (    7 More Missing: see the .ckf listing file)
PLAT976_ALERT_2_C Check Calcd Resid. Dens.  0.89Ang From O14     .      -0.45 eA-3
PLAT977_ALERT_2_C Check Negative Difference Density on H27A      .      -0.31 eA-3
```

Alert level G

```
FORMU01_ALERT_1_G  There is a discrepancy between the atom counts in the
            _chemical_formula_sum and _chemical_formula_moiety. This is
            usually due to the moiety formula being in the wrong format.
            Atom count from _chemical_formula_sum:   C144 H120 Co20 N48 O60
            Atom count from _chemical_formula_moiety:C144 H120 Co20 N42 O42
FORMU01_ALERT_2_G  There is a discrepancy between the atom counts in the
            _chemical_formula_sum and the formula from the _atom_site* data.
            Atom count from _chemical_formula_sum:C144 H120 Co20 N48 O60
            Atom count from the _atom_site data:  C144 H120 Co20 N37. O27.00239
CELLZ01_ALERT_1_G Difference between formula and atom_site contents detected.
CELLZ01_ALERT_1_G ALERT: Large difference may be due to a
            symmetry error - see SYMMG tests
           From the CIF: _cell_formula_units_Z    2
           From the CIF: _chemical_formula_sum  C144 H120 Co20 N48 O60
           TEST: Compare cell contents of formula and atom_site data

           atom    Z*formula  cif sites diff
           C        288.00    288.00    0.00
           H        240.00    240.00    0.00
           Co        40.00     40.00    0.00
           N         96.00     74.00   22.00
           O        120.00     54.00   66.00
PLAT002_ALERT_2_G Number of Distance or Angle Restraints on AtSite         42 Note
PLAT003_ALERT_2_G Number of Uiso or U(i,j)  Restrained non-H-Atoms         41 Report
PLAT004_ALERT_5_G Polymeric Structure Found with Maximum Dimension          3 Info
PLAT007_ALERT_5_G Number of Unrefined Donor-H Atoms ..............          8 Report
              H2A   H5    H5A   H10A  H2    H3    H14   H27A
PLAT032_ALERT_4_G Std. Uncertainty on Flack Parameter Value High .      0.300 Report
PLAT041_ALERT_1_G Calc. and Reported SumFormula    Strings  Differ     Please Check
              Calc: C288 H240 Co40 N74 O54
              Rep.: C144 H120 Co20 N48 O60
PLAT042_ALERT_1_G Calc. and Reported MoietyFormula Strings  Differ     Please Check
              Calc: 3(C48 H40 Co6.67 N12.33 O9), C144 H120 Co20 N3
                    7 O27
              Rep.: C144 H120 Co20 N37 O27, 5[NO3], 6[NO3]
PLAT045_ALERT_1_G Calculated and Reported Z Differ by a Factor ...      0.500 Check
PLAT072_ALERT_2_G SHELXL First  Parameter in WGHT  Unusually Large       0.18 Report
PLAT083_ALERT_2_G SHELXL Second Parameter in WGHT  Unusually Large     108.20 Why ?
PLAT172_ALERT_4_G The CIF-Embedded .res File Contains DFIX Records         43 Report
PLAT174_ALERT_4_G The CIF-Embedded .res File Contains FLAT Records          4 Report
PLAT178_ALERT_4_G The CIF-Embedded .res File Contains SIMU Records          4 Report
PLAT186_ALERT_4_G The CIF-Embedded .res File Contains ISOR Records          3 Report
PLAT188_ALERT_3_G A Non-default SIMU Restraint Value has been used     0.0010 Report
PLAT188_ALERT_3_G A Non-default SIMU Restraint Value has been used     0.0010 Report
PLAT188_ALERT_3_G A Non-default SIMU Restraint Value has been used     0.0100 Report
PLAT188_ALERT_3_G A Non-default SIMU Restraint Value has been used     0.0100 Report
PLAT299_ALERT_4_G Atom Site Occupancy Constrained at ............         0.5 Check
              N4      N5      N10     C2      C3      C6      C7      C8
              C9      C11     C12     C13     C14     C15     C16     H5A
              H7      H9      H10A    H13     H14A    H15     H16     N1
              N2      N3      C1      C1A     C1B     C1C     C1D     C1E
              C4      C5      C10     C17     C18     C19     H1B     H1C
              H1D     H1E     H2      H3      H10     H18
PLAT300_ALERT_4_G Atom Site Occupancy of O9         Constrained at     0.0417 Check
PLAT300_ALERT_4_G Atom Site Occupancy of O10        Constrained at     0.0417 Check
PLAT300_ALERT_4_G Atom Site Occupancy of O11        Constrained at     0.0417 Check
```

```
PLAT300_ALERT_4_G Atom Site Occupancy of N8         Constrained at     0.0417 Check
PLAT300_ALERT_4_G Atom Site Occupancy of O12        Constrained at     0.0417 Check
PLAT300_ALERT_4_G Atom Site Occupancy of O13        Constrained at     0.0417 Check
PLAT300_ALERT_4_G Atom Site Occupancy of O15        Constrained at     0.0417 Check
PLAT300_ALERT_4_G Atom Site Occupancy of N9         Constrained at     0.0417 Check
PLAT301_ALERT_3_G Main Residue  Disorder .............(Resd    1)        73% Note
PLAT301_ALERT_3_G Main Residue  Disorder .............(Resd    2)        81% Note
PLAT303_ALERT_2_G Full Occupancy Atom H14       with # Connections      2.00 Check
PLAT605_ALERT_4_G Largest Solvent Accessible VOID in the Structure        77 A**3
PLAT721_ALERT_1_G Bond    Calc     0.87000, Rep     0.85970 Dev...      0.01 Ang.
              N5       -H5A             1_555   1_555 ........     #   35 Check
PLAT721_ALERT_1_G Bond    Calc     0.85000, Rep     0.86010 Dev...      0.01 Ang.
              N2       -H2              1_555   1_555 ........     #   60 Check
PLAT722_ALERT_1_G Angle   Calc      116.00, Rep      117.10 Dev...      1.10 Degree
              C8      -N10     -H10A       1_555  1_555  1_555     #  113 Check
PLAT722_ALERT_1_G Angle   Calc      121.00, Rep      119.80 Dev...      1.20 Degree
              C12     -C13     -H13        1_555  1_555  1_555     #  120 Check
PLAT722_ALERT_1_G Angle   Calc      105.00, Rep      103.80 Dev...      1.20 Degree
              C1      -N2      -H2         1_555  1_555  1_555     #  143 Check
PLAT780_ALERT_1_G Coordinates do not Form a Properly Connected Set     Please Do !
PLAT789_ALERT_4_G Atoms with Negative _atom_site_disorder_group  #        54 Check
PLAT811_ALERT_5_G No ADDSYM Analysis: Too Many Excluded Atoms ....         ! Info
PLAT822_ALERT_4_G CIF-embedded .res Contains Negative PART Numbers         2 Check
PLAT860_ALERT_3_G Number of Least-Squares Restraints ............        341 Note
PLAT868_ALERT_4_G ALERTS Due to the Use of _smtbx_masks Suppressed         ! Info
PLAT910_ALERT_3_G Missing FCF Reflection(s) Below Theta(Min)[Deg]=      1.70 Note
                0  0  1,   0  1  1,
PLAT912_ALERT_4_G Missing # of FCF Reflections Above STh/L=  0.600       271 Note
PLAT913_ALERT_3_G Missing # of Very Strong Reflections in FCF ....         1 Note
                0  1  1,
PLAT915_ALERT_3_G No Flack x Check Done: Low Friedel Pair Coverage        54 %
PLAT916_ALERT_2_G Hooft y and Flack x Parameter Values Differ by .      0.16 Check
PLAT941_ALERT_3_G Average HKL Measurement Multiplicity ..........        4.5 Low
PLAT950_ALERT_5_G Calculated (ThMax) and CIF-Reported Hmax Differ          6 Units
PLAT955_ALERT_1_G Reported (CIF) and Actual (FCF) Lmax Differ by .         1 Units
PLAT969_ALERT_5_G The 'Henn et al.' R-Factor-gap value ..........      9.074 Note
              Predicted wR2: Based on SigI**2  5.71 or SHELX Weight 49.14
```

---

1 **ALERT level A** = Most likely a serious problem - resolve or explain
9 **ALERT level B** = A potentially serious problem, consider carefully
16 **ALERT level C** = Check. Ensure it is not caused by an omission or oversight
55 **ALERT level G** = General information/check it is not something unexpected

13 ALERT type 1 CIF construction/syntax error, inconsistent or missing data
26 ALERT type 2 Indicator that the structure model may be wrong or deficient
18 ALERT type 3 Indicator that the structure quality may be low
19 ALERT type 4 Improvement, methodology, query or suggestion
5 ALERT type 5 Informative message, check

It is advisable to attempt to resolve as many as possible of the alerts in all categories. Often the minor alerts point to easily fixed oversights, errors and omissions in your CIF or refinement strategy, so attention to these fine details can be worthwhile. In order to resolve some of the more serious problems it may be necessary to carry out additional measurements or structure refinements. However, the purpose of your study may justify the reported deviations and the more serious of these should normally be commented upon in the discussion or experimental section of a paper or in the "special_details" fields of the CIF. checkCIF was carefully designed to identify outliers and unusual parameters, but every test has its limitations and alerts that are not important in a particular case may appear. Conversely, the absence of alerts does not guarantee there are no aspects of the results needing attention. It is up to the individual to critically assess their own results and, if necessary, seek expert advice.

Publication of your CIF in IUCr journals

A basic structural check has been run on your CIF. These basic checks will be run on all CIFs submitted for publication in IUCr journals (*Acta Crystallographica*, *Journal of Applied Crystallography*, *Journal of Synchrotron Radiation*); however, if you intend to submit to *Acta Crystallographica Section C* or *E* or *IUCrData*, you should make sure that full publication checks are run on the final version of your CIF prior to submission.

Publication of your CIF in other journals

Please refer to the *Notes for Authors* of the relevant journal for any special instructions relating to CIF submission.

PLATON version of 04/06/2025; check.def file version of 30/05/2025

Datablock wwlnjcubic_auto - ellipsoid plot

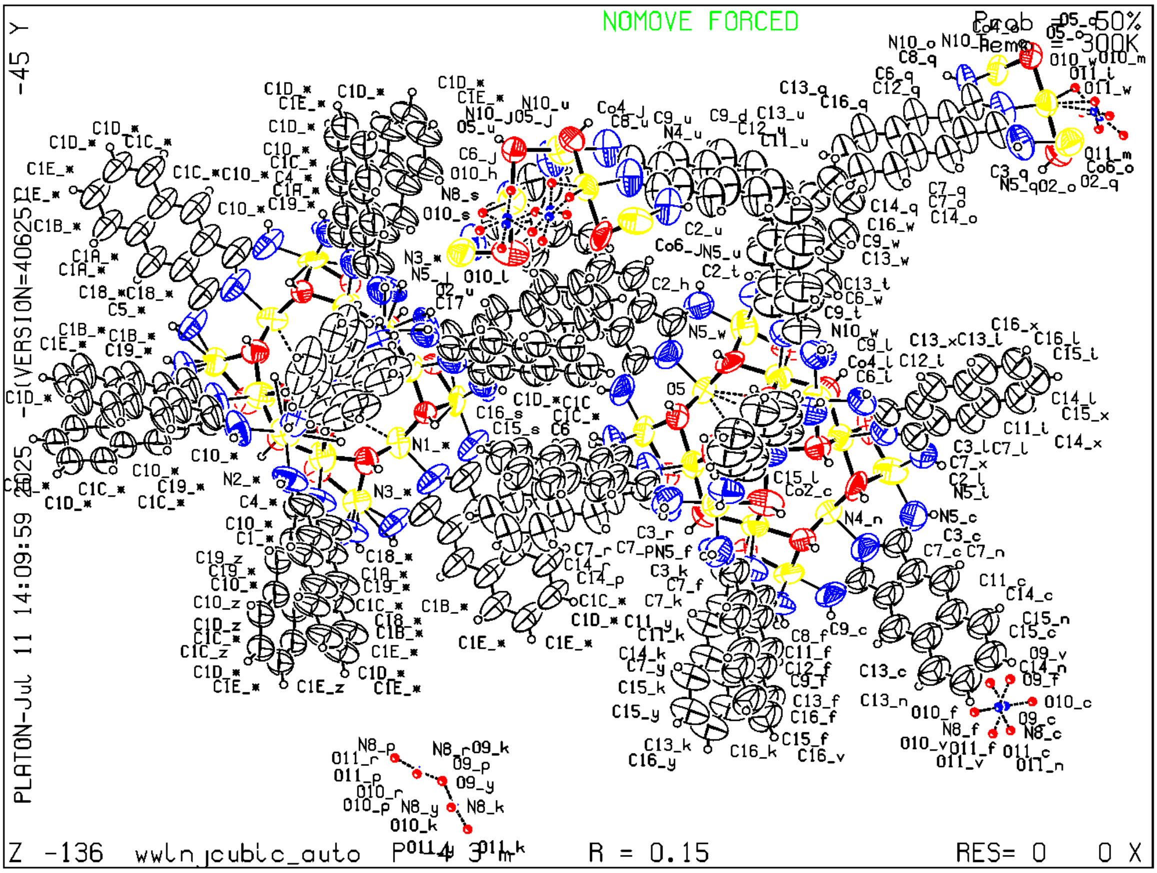

Supplement: Supplementary file 2 — Supporting cif files [file ADVS-13-e19498-s001.zip › Checkcif (activated 1).pdf]
