# Supplementary material for: Engineering Active Metal and Nonmetal Sites in Porous Structures of Metal‐Hydroxide Clusters for Enhanced D2/H2 Uptake and Separation
Source: Adv Sci (Weinh). 2025 Dec 14;13(13):e19498. doi: 10.1002/advs.202519498 (PMC12955987; doi:10.1002/advs.202519498)

checkCIF/PLATON report

Structure factors have been supplied for datablock(s) aaa

THIS REPORT IS FOR GUIDANCE ONLY. IF USED AS PART OF A REVIEW PROCEDURE FOR PUBLICATION, IT SHOULD NOT REPLACE THE EXPERTISE OF AN EXPERIENCED CRYSTALLOGRAPHIC REFEREE.

No syntax errors found. CIF dictionary Interpreting this report

Datablock: aaa

Bond precision:	C-C = 0.0119 A		Wavelength=1.54178
Cell:	a=24.5688(4)	b=20.5400(3)	c=25.7456(5)
	alpha=90	beta=92.609(2)	gamma=90
Temperature:	293 K		

	Calculated	Reported
Volume	12978.9(4)	12978.9(4)
Space group	P 21/n	P 1 21/n 1
Hall group	-P 2yn	-P 2yn
Moiety formula	C144 H120 Co20 N36 O24, 12(N O3) [+ solvent]	C144 H120 Co20 N37 O27, 11(N O3)
Sum formula	C144 H120 Co20 N48 O60 [+ solvent]	C144 H120 Co20 N48 O60
Mr	4661.48	4661.47
Dx,g cm-3	1.193	1.193
Z	2	2
Mu (mm-1)	10.287	10.287
F000	4680.0	4680.0
F000'	4600.74	
h,k,lmax	30,25,32	30,24,32
Nref	27298	26141
Tmin,Tmax	0.324,0.329	
Tmin'	0.245	

Correction method= Not given

Data completeness= 0.958 Theta(max)= 76.583

R(reflections)= 0.0900(16705) wR2(reflections)= 0.2909(26141)

S = 1.041 Npar= 1325

The following ALERTS were generated. Each ALERT has the format
test-name_ALERT_alert-type_alert-level.
Click on the hyperlinks for more details of the test.

Alert level B

```
PLAT430_ALERT_2_B Short Inter D...A Contact  O1K      ..O29      .       2.65 Ang.
                                     -1/2+x,3/2-y,-1/2+z  =      4_575 Check
```

Alert level C

```
PLAT084_ALERT_3_C High wR2 Value (i.e. > 0.25) ..................       0.29 Report
PLAT202_ALERT_3_C Isotropic non-H Atoms in Anion/Solvent .........         8 Check
              O1L     O1M     O1N     N1H     O2      O13     O16     N18
PLAT220_ALERT_2_C NonSolvent   Resd 1  C   Ueq(max)/Ueq(min) Range        3.4 Ratio
PLAT234_ALERT_4_C Large Hirshfeld Difference C3       --C58      .       0.20 Ang.
PLAT234_ALERT_4_C Large Hirshfeld Difference C7       --C64      .       0.17 Ang.
PLAT234_ALERT_4_C Large Hirshfeld Difference C13      --C23      .       0.22 Ang.
PLAT241_ALERT_2_C High   'MainMol' Ueq as Compared to Neighbors of        C24 Check
PLAT241_ALERT_2_C High   'MainMol' Ueq as Compared to Neighbors of        C52 Check
PLAT242_ALERT_2_C Low    'MainMol' Ueq as Compared to Neighbors of         C4 Check
PLAT243_ALERT_4_C High   'Solvent' Ueq as Compared to Neighbors of        N18 Check
PLAT244_ALERT_4_C Low    'Solvent' Ueq as Compared to Neighbors of        N1H Check
PLAT244_ALERT_4_C Low    'Solvent' Ueq as Compared to Neighbors of         N7 Check
PLAT341_ALERT_3_C Low Bond Precision on  C-C Bonds ...............    0.01189 Ang.
PLAT369_ALERT_2_C Long   C(sp2)-C(sp2) Bond  C15      - C24      .       1.53 Ang.
PLAT905_ALERT_3_C Negative K value in the Analysis of Variance ...     -0.635 Report
PLAT911_ALERT_3_C Missing FCF Refl Between Thmin & STh/L=    0.600         41 Report
                0 24  0,   1 24  1,   4  2  1,  15 21  1, -13 21  2, -12 21  2,
                0  0  2,   0  1  2,   1  1  2,   3  9  2,  -1  0  3,   1  0  3,
                0  4  4,   2  3  4,  -7  5  5,  -6 15  5,  -2  2  5,  -1  3  5,
                2  3  5,  -7 16  6,   3  4  6,  -5  5  7,  -1  0  7,  -7 22 12,
               -6 22 12,  -5 22 12,  -4 22 12,   9 21 12,  -5 22 13,  -4 22 13,
                    (   11 More Missing: see the .ckf listing file)
PLAT913_ALERT_3_C Missing # of Very Strong Reflections in FCF ....          4 Note
                1  1  0,  -1  0  1,   0  1  1,   1  0  1,
PLAT918_ALERT_3_C Reflection(s) with I(obs) much Smaller I(calc) .         12 Check
PLAT971_ALERT_2_C Check Calcd Resid. Dens.  0.21Ang From O1P            1.98 eA-3
PLAT971_ALERT_2_C Check Calcd Resid. Dens.  0.39Ang From O1R            1.97 eA-3
PLAT971_ALERT_2_C Check Calcd Resid. Dens.  0.19Ang From O1C            1.70 eA-3
PLAT971_ALERT_2_C Check Calcd Resid. Dens.  0.40Ang From O1T            1.70 eA-3
PLAT971_ALERT_2_C Check Calcd Resid. Dens.  0.18Ang From O1G            1.66 eA-3
PLAT971_ALERT_2_C Check Calcd Resid. Dens.  0.52Ang From N1G            1.64 eA-3
PLAT975_ALERT_2_C Check Calcd Resid. Dens.  1.10Ang From O16     .       0.84 eA-3
PLAT975_ALERT_2_C Check Calcd Resid. Dens.  0.87Ang From O2      .       0.63 eA-3
```

Alert level G

```
PLAT002_ALERT_2_G Number of Distance or Angle Restraints on AtSite         67 Note
PLAT003_ALERT_2_G Number of Uiso or U(i,j)  Restrained non-H-Atoms         68 Report
PLAT007_ALERT_5_G Number of Unrefined Donor-H Atoms ..............         26 Report
              H1    H1CA  H1A   H1DA  H3    H4    H4A   H5    H5A   H6    H7
              H8    H8A   H10   H10A  H11   H12   H14   H15   H15A  H16   H17
PLAT042_ALERT_1_G Calc. and Reported MoietyFormula Strings  Differ     Please Check
              Calc: C144 H120 Co20 N36 O24, 12(N O3)
```

```
               Rep.: C144 H120 Co20 N37 O27, 11(N O3)
PLAT072_ALERT_2_G SHELXL First  Parameter in WGHT  Unusually Large       0.20 Report
PLAT172_ALERT_4_G The CIF-Embedded .res File Contains DFIX Records         65 Report
PLAT174_ALERT_4_G The CIF-Embedded .res File Contains FLAT Records         11 Report
PLAT176_ALERT_4_G The CIF-Embedded .res File Contains SADI Records          8 Report
PLAT177_ALERT_4_G The CIF-Embedded .res File Contains DELU Records          1 Report
PLAT178_ALERT_4_G The CIF-Embedded .res File Contains SIMU Records         11 Report
PLAT187_ALERT_4_G The CIF-Embedded .res File Contains RIGU Records          1 Report
PLAT188_ALERT_3_G A Non-default SIMU Restraint Value has been used     0.0010 Report
PLAT188_ALERT_3_G A Non-default SIMU Restraint Value has been used     0.0010 Report
PLAT188_ALERT_3_G A Non-default SIMU Restraint Value has been used     0.0010 Report
PLAT188_ALERT_3_G A Non-default SIMU Restraint Value has been used     0.0010 Report
PLAT188_ALERT_3_G A Non-default SIMU Restraint Value has been used     0.0100 Report
PLAT188_ALERT_3_G A Non-default SIMU Restraint Value has been used     0.0100 Report
PLAT188_ALERT_3_G A Non-default SIMU Restraint Value has been used     0.0100 Report
PLAT188_ALERT_3_G A Non-default SIMU Restraint Value has been used     0.0100 Report
PLAT188_ALERT_3_G A Non-default SIMU Restraint Value has been used     0.0010 Report
PLAT188_ALERT_3_G A Non-default SIMU Restraint Value has been used     0.0010 Report
PLAT191_ALERT_3_G A Non-default SADI Restraint Value has been used     0.0400 Report
PLAT191_ALERT_3_G A Non-default SADI Restraint Value has been used     0.0400 Report
PLAT191_ALERT_3_G A Non-default SADI Restraint Value has been used     0.0400 Report
PLAT191_ALERT_3_G A Non-default SADI Restraint Value has been used     0.0400 Report
PLAT191_ALERT_3_G A Non-default SADI Restraint Value has been used     0.0400 Report
PLAT191_ALERT_3_G A Non-default SADI Restraint Value has been used     0.0400 Report
PLAT192_ALERT_3_G A Non-default DELU Restraint Value for SecondPar     0.0200 Report
PLAT199_ALERT_1_G Reported _cell_measurement_temperature ..... (K)        293 Check
PLAT200_ALERT_1_G Reported   _diffrn_ambient_temperature ..... (K)        293 Check
PLAT232_ALERT_2_G Hirshfeld Test Diff (M-X)  Co5      --O17      .        7.0 s.u.
PLAT232_ALERT_2_G Hirshfeld Test Diff (M-X)  Co5      --N12      .        5.3 s.u.
PLAT232_ALERT_2_G Hirshfeld Test Diff (M-X)  Co7      --O10      .        7.5 s.u.
PLAT232_ALERT_2_G Hirshfeld Test Diff (M-X)  Co10     --O1       .        7.0 s.u.
PLAT232_ALERT_2_G Hirshfeld Test Diff (M-X)  Co10     --O4       .        7.0 s.u.
PLAT232_ALERT_2_G Hirshfeld Test Diff (M-X)  Co10     --O7       .        6.5 s.u.
PLAT299_ALERT_4_G Atom Site Occupancy Constrained at ............         0.5 Check
               O1A     O1B     O19     N1A     O1I     O1K     O1R     N1J
               O1J     O20     O29     N1G     O1O     O1P     O1Q     N1I
PLAT300_ALERT_4_G Atom Site Occupancy of O1C        Constrained at     0.3333 Check
PLAT300_ALERT_4_G Atom Site Occupancy of O1D        Constrained at     0.3333 Check
PLAT300_ALERT_4_G Atom Site Occupancy of O1E        Constrained at     0.3333 Check
PLAT300_ALERT_4_G Atom Site Occupancy of N1E        Constrained at     0.3333 Check
PLAT300_ALERT_4_G Atom Site Occupancy of O1F        Constrained at     0.3333 Check
PLAT300_ALERT_4_G Atom Site Occupancy of O1G        Constrained at     0.3333 Check
PLAT300_ALERT_4_G Atom Site Occupancy of O1H        Constrained at     0.3333 Check
PLAT300_ALERT_4_G Atom Site Occupancy of N1F        Constrained at     0.3333 Check
PLAT300_ALERT_4_G Atom Site Occupancy of O1S        Constrained at     0.3333 Check
PLAT300_ALERT_4_G Atom Site Occupancy of O1T        Constrained at     0.3333 Check
PLAT300_ALERT_4_G Atom Site Occupancy of O1U        Constrained at     0.3333 Check
PLAT300_ALERT_4_G Atom Site Occupancy of N1K        Constrained at     0.3333 Check
PLAT301_ALERT_3_G Main Residue  Disorder .............(Resd    1)        13% Note
PLAT302_ALERT_4_G Anion/Solvent/Minor-Residue Disorder (Resd    2)       100% Note
PLAT302_ALERT_4_G Anion/Solvent/Minor-Residue Disorder (Resd    3)       100% Note
PLAT302_ALERT_4_G Anion/Solvent/Minor-Residue Disorder (Resd    4)       100% Note
PLAT302_ALERT_4_G Anion/Solvent/Minor-Residue Disorder (Resd    6)       100% Note
PLAT302_ALERT_4_G Anion/Solvent/Minor-Residue Disorder (Resd    9)       100% Note
PLAT302_ALERT_4_G Anion/Solvent/Minor-Residue Disorder (Resd   10)       100% Note
PLAT302_ALERT_4_G Anion/Solvent/Minor-Residue Disorder (Resd   11)       100% Note
PLAT304_ALERT_4_G Non-Integer Number of Atoms in ..... (Resd    9)       1.33 Check
```

```
PLAT304_ALERT_4_G Non-Integer Number of Atoms in ..... (Resd   10)       1.33 Check
PLAT304_ALERT_4_G Non-Integer Number of Atoms in ..... (Resd   11)       1.33 Check
PLAT432_ALERT_2_G Short Inter X...Y Contact  O1E      ..C28      .       2.96 Ang.
                                               1-x,1-y,1-z  =     3_666 Check
PLAT432_ALERT_2_G Short Inter X...Y Contact  O1E      ..C42      .       2.98 Ang.
                                               1-x,1-y,1-z  =     3_666 Check
PLAT432_ALERT_2_G Short Inter X...Y Contact  O1E      ..C6       .       3.00 Ang.
                                               1-x,1-y,1-z  =     3_666 Check
PLAT606_ALERT_4_G Solvent Accessible VOID(S) in  Crystal Structure          ! Info
PLAT720_ALERT_4_G Number of Unusual/Non-Standard Labels .........          2 Note
              H1CA    H1DA
PLAT789_ALERT_4_G Atoms with Negative _atom_site_disorder_group  #          4 Check
PLAT794_ALERT_5_G Tentative Bond Valency for Co7       (III)     .       2.89 Info
PLAT794_ALERT_5_G Tentative Bond Valency for Co10      (III)     .       3.01 Info
PLAT822_ALERT_4_G CIF-embedded .res Contains Negative PART Numbers          1 Check
PLAT860_ALERT_3_G Number of Least-Squares Restraints ............         410 Note
PLAT868_ALERT_4_G ALERTS Due to the Use of _smtbx_masks Suppressed          ! Info
PLAT910_ALERT_3_G Missing FCF Reflection(s) Below Theta(Min)[Deg]=       3.25 Note
                1  1  0,  -1  0  1,   0  1  1,   1  0  1,
PLAT912_ALERT_4_G Missing # of FCF Reflections Above STh/L=  0.600       1000 Note
PLAT933_ALERT_2_G Number of HKL-OMIT Records in Embedded .res File         12 Note
               -7  5  5,  -5  5  7,  -2  2  5,  -1  3  5,   0  1  2,   0  4  4,
                1  0  3,   2  3  4,   2  3  5,   3  4  6,   3  9  2,   4  2  1,
PLAT941_ALERT_3_G Average HKL Measurement Multiplicity ..........         3.4 Low
PLAT969_ALERT_5_G The 'Henn et al.' R-Factor-gap value ..........       5.360 Note
              Predicted wR2: Based on SigI**2  5.43 or SHELX Weight 27.95
PLAT978_ALERT_2_G Number C-C Bonds with Positive Residual Density.          0 Info
PLAT992_ALERT_5_G Repd & Actual _reflns_number_gt Values Differ by          2 Check
```

---

0 **ALERT level A** = Most likely a serious problem - resolve or explain
1 **ALERT level B** = A potentially serious problem, consider carefully
26 **ALERT level C** = Check. Ensure it is not caused by an omission or oversight
78 **ALERT level G** = General information/check it is not something unexpected

3 ALERT type 1 CIF construction/syntax error, inconsistent or missing data
28 ALERT type 2 Indicator that the structure model may be wrong or deficient
28 ALERT type 3 Indicator that the structure quality may be low
41 ALERT type 4 Improvement, methodology, query or suggestion
5 ALERT type 5 Informative message, check

It is advisable to attempt to resolve as many as possible of the alerts in all categories. Often the minor alerts point to easily fixed oversights, errors and omissions in your CIF or refinement strategy, so attention to these fine details can be worthwhile. In order to resolve some of the more serious problems it may be necessary to carry out additional measurements or structure refinements. However, the purpose of your study may justify the reported deviations and the more serious of these should normally be commented upon in the discussion or experimental section of a paper or in the "special_details" fields of the CIF. checkCIF was carefully designed to identify outliers and unusual parameters, but every test has its limitations and alerts that are not important in a particular case may appear. Conversely, the absence of alerts does not guarantee there are no aspects of the results needing attention. It is up to the individual to critically assess their own results and, if necessary, seek expert advice.

Publication of your CIF in IUCr journals

A basic structural check has been run on your CIF. These basic checks will be run on all CIFs submitted for publication in IUCr journals (*Acta Crystallographica*, *Journal of Applied Crystallography*, *Journal of Synchrotron Radiation*); however, if you intend to submit to *Acta Crystallographica Section C* or *E* or *IUCrData*, you should make sure that full publication checks are run on the final version of your CIF prior to submission.

Publication of your CIF in other journals

Please refer to the *Notes for Authors* of the relevant journal for any special instructions relating to CIF submission.

PLATON version of 04/06/2025; check.def file version of 30/05/2025

Datablock aaa - ellipsoid plot

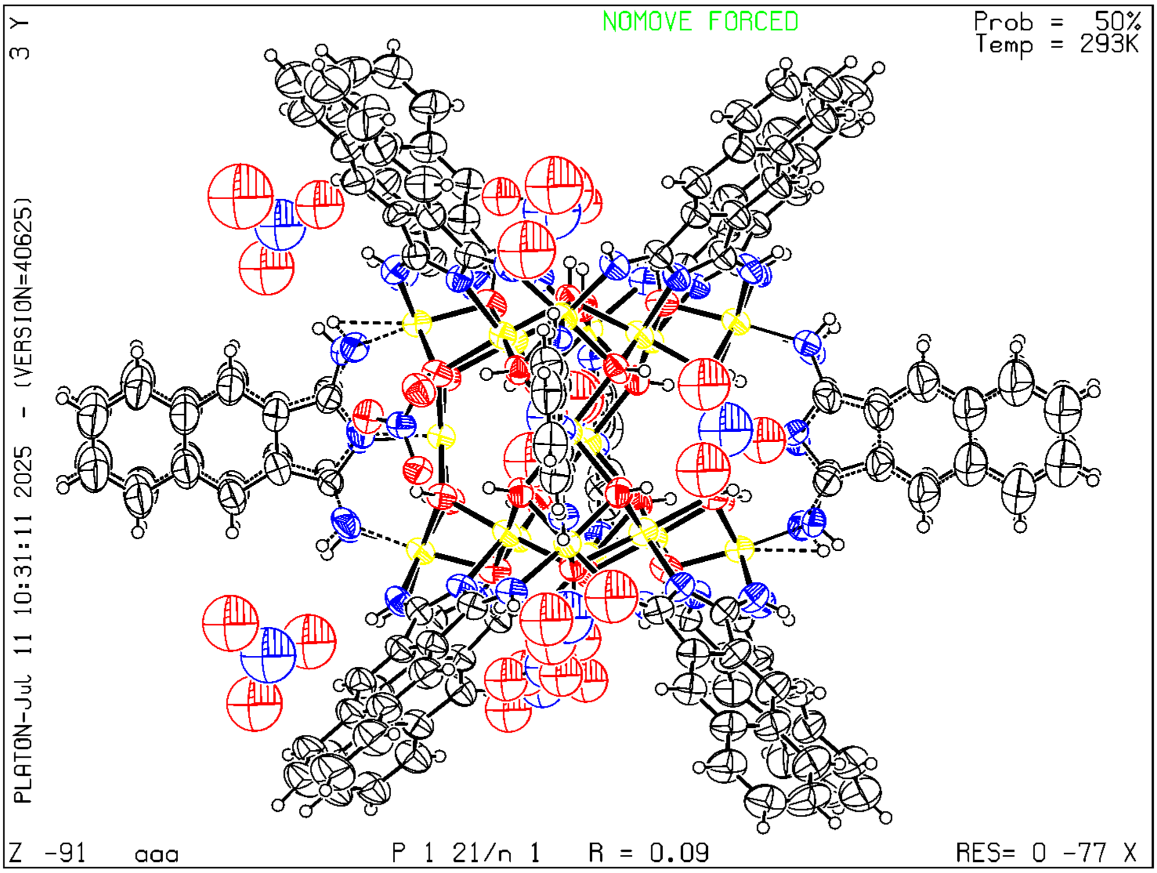

Supplement: Supplementary file 2 — Supporting cif files [file ADVS-13-e19498-s001.zip › Checkcif (compound 1).pdf]
