# Supplementary material for: Engineering Active Metal and Nonmetal Sites in Porous Structures of Metal‐Hydroxide Clusters for Enhanced D2/H2 Uptake and Separation
Source: Adv Sci (Weinh). 2025 Dec 14;13(13):e19498. doi: 10.1002/advs.202519498 (PMC12955987; doi:10.1002/advs.202519498)

# checkCIF/PLATON report

Structure factors have been supplied for datablock(s) xzz-240831-191928tetra_auto

THIS REPORT IS FOR GUIDANCE ONLY. IF USED AS PART OF A REVIEW PROCEDURE FOR PUBLICATION, IT SHOULD NOT REPLACE THE EXPERTISE OF AN EXPERIENCED CRYSTALLOGRAPHIC REFEREE.

No syntax errors found. CIF dictionary Interpreting this report

## Datablock: xzz-240831-191928tetra_auto

---

```
Bond precision:   Cu- O = 0.0058 A         Wavelength=1.34050

Cell:              a=19.5796(2)      b=19.5796(2)      c=28.3746(5)
                   alpha=90          beta=90           gamma=90
Temperature:       100 K
```

| | Calculated | Reported |
|---|---|---|
| Volume | 10877.7(3) | 10877.7(3) |
| Space group | I 4/m m m | I 4/m m m |
| Hall group | -I 4 2 | -I 4 2 |
| Moiety formula | C96 H104 Cl Cu20 N24 O36, 3(C H O2), 2(C2 H8 N), 4(Cl O4), 2(Cl | C96 H104 Cl Cu20 N24 O36, 2(Cl), 4(Cl O4), 4(C H4 N0.5), 8(C0.3 |
| Sum formula | C103 H123 Cl7 Cu20 N26 O58 [+ solvent] | C151 H235 Cl7 Cu20 N42 O74 |
| Mr | 4172.42 | 5341.75 |
| Dx,g cm-3 | 1.274 | 1.631 |
| Z | 2 | 2 |
| Mu (mm-1) | 11.222 | 11.363 |
| F000 | 4172.0 | 5452.0 |
| F000’ | 4075.34 | |
| h,k,lmax | 25,25,36 | 25,22,36 |
| Nref | 3453 | 3415 |
| Tmin,Tmax | 0.302,0.303 | 0.357,1.000 |
| Tmin’ | 0.193 | |

```
Correction method= # Reported T Limits: Tmin=0.357 Tmax=1.000
AbsCorr = MULTI-SCAN

Data completeness= 0.989            Theta(max)= 59.998
```

```
R(reflections)= 0.1192( 3020)                    wR2(reflections)=
                                                 0.3225( 3415)
S = 1.089                   Npar= 261
```

---

The following ALERTS were generated. Each ALERT has the format
      **test-name_ALERT_alert-type_alert-level.**
Click on the hyperlinks for more details of the test.

---

### Alert level B

```
PLAT420_ALERT_2_B D-H Bond Without Acceptor  O6       --H6B      .     Please Check
```

---

### Alert level C

```
DIFMN02_ALERT_2_C  The minimum difference density is < -0.1*ZMAX*0.75
            _refine_diff_density_min given =     -2.460
            Test value =     -2.175
DIFMN03_ALERT_1_C  The minimum difference density is < -0.1*ZMAX*0.75
            The relevant atom site should be identified.
RADNW01_ALERT_1_C  The radiation wavelength lies outside the expected range
            for the supplied radiation type. Expected range 1.34130-1.34150
            Wavelength given =    1.34050
PLAT082_ALERT_2_C High R1 Value ..................................       0.12 Report
PLAT084_ALERT_3_C High wR2 Value (i.e. > 0.25) ...................       0.32 Report
PLAT098_ALERT_2_C Large Reported Min.  (Negative) Residual Density      -2.46 eA-3
PLAT250_ALERT_2_C Large U3/U1 Ratio for <U(i,j)> Tensor(Resd    1)        2.4 Note
PLAT906_ALERT_3_C Large K Value in the Analysis of Variance ......      2.927 Check
PLAT911_ALERT_3_C Missing FCF Refl Between Thmin & STh/L=    0.600          3 Report
                0  8  0,   1  1  2,   0  0 12,
PLAT918_ALERT_3_C Reflection(s) with I(obs) much Smaller I(calc) .          2 Check
PLAT971_ALERT_2_C Check Calcd Resid. Dens.  0.40Ang From N9              2.32 eA-3
PLAT971_ALERT_2_C Check Calcd Resid. Dens.  0.51Ang From Cl3             1.91 eA-3
PLAT972_ALERT_2_C Check Calcd Resid. Dens.  0.44Ang From Cl3            -2.33 eA-3
PLAT972_ALERT_2_C Check Calcd Resid. Dens.  0.18Ang From Cl3            -2.32 eA-3
PLAT975_ALERT_2_C Check Calcd Resid. Dens.  1.09Ang From O12      .      0.80 eA-3
PLAT976_ALERT_2_C Check Calcd Resid. Dens.  0.94Ang From N2       .     -0.77 eA-3
PLAT977_ALERT_2_C Check Negative Difference Density on H2         .     -0.52 eA-3
PLAT977_ALERT_2_C Check Negative Difference Density on H5         .     -0.40 eA-3
PLAT977_ALERT_2_C Check Negative Difference Density on H14        .     -0.31 eA-3
```

---

### Alert level G

```
FORMU01_ALERT_1_G  There is a discrepancy between the atom counts in the
            _chemical_formula_sum and _chemical_formula_moiety. This is
            usually due to the moiety formula being in the wrong format.
            Atom count from _chemical_formula_sum:   C151 H235 Cl7 Cu20 N42 O74
            Atom count from _chemical_formula_moiety:C103 H123 Cl7 Cu20 N26 O52
FORMU01_ALERT_2_G  There is a discrepancy between the atom counts in the
            _chemical_formula_sum and the formula from the _atom_site* data.
            Atom count from _chemical_formula_sum:C151 H235 Cl7 Cu20 N42 O74
            Atom count from the _atom_site data:  C103 H123 Cl7 Cu20 N26 O58
ABSMU01_ALERT_1_G  Calculation of _exptl_absorpt_correction_mu
                not performed for this radiation type.
CELLZ01_ALERT_1_G Difference between formula and atom_site contents detected.
CELLZ01_ALERT_1_G ALERT: Large difference may be due to a
```

```
           symmetry error - see SYMMG tests
          From the CIF: _cell_formula_units_Z    2
          From the CIF: _chemical_formula_sum  C151 H235 Cl7 Cu20 N42 O74
          TEST: Compare cell contents of formula and atom_site data

          atom    Z*formula  cif sites diff
          C        302.00    206.00   96.00
          H        470.00    246.00  224.00
          Cl        14.00     14.00    0.00
          Cu        40.00     40.00    0.00
          N         84.00     52.00   32.00
          O        148.00    116.00   32.00
PLAT002_ALERT_2_G Number of Distance or Angle Restraints on AtSite         29 Note
PLAT003_ALERT_2_G Number of Uiso or U(i,j)  Restrained non-H-Atoms         25 Report
PLAT007_ALERT_5_G Number of Unrefined Donor-H Atoms .............           7 Report
             H1    H3    H4    H6A   H6B   H9A   H9B
PLAT041_ALERT_1_G Calc. and Reported SumFormula    Strings  Differ     Please Check
             Calc: C103 H123 Cl7 Cu20 N26 O58
             Rep.: C151 H235 Cl7 Cu20 N42 O74
PLAT042_ALERT_1_G Calc. and Reported MoietyFormula Strings  Differ     Please Check
             Calc: C96 H104 Cl Cu20 N24 O36, 3(C H O2), 2(C2 H8 N
                   ), 4(Cl O4), 2(Cl)
             Rep.: C96 H104 Cl Cu20 N24 O36, 2(Cl), 4(Cl O4), 4(C
                    H4 N0.5), 8(C0.375 H0.375 O0.75)
PLAT051_ALERT_1_G Mu(calc) and Mu(cif) Ratio Differs from 1.0 by .       1.24 %
PLAT072_ALERT_2_G SHELXL First  Parameter in WGHT  Unusually Large       0.15 Report
PLAT083_ALERT_2_G SHELXL Second Parameter in WGHT  Unusually Large     377.37 Why ?
PLAT171_ALERT_4_G The CIF-Embedded .res File Contains EADP Records          2 Report
PLAT172_ALERT_4_G The CIF-Embedded .res File Contains DFIX Records         26 Report
PLAT174_ALERT_4_G The CIF-Embedded .res File Contains FLAT Records          1 Report
PLAT176_ALERT_4_G The CIF-Embedded .res File Contains SADI Records          3 Report
PLAT178_ALERT_4_G The CIF-Embedded .res File Contains SIMU Records          3 Report
PLAT186_ALERT_4_G The CIF-Embedded .res File Contains ISOR Records          1 Report
PLAT188_ALERT_3_G A Non-default SIMU Restraint Value has been used     0.0100 Report
PLAT188_ALERT_3_G A Non-default SIMU Restraint Value has been used     0.0100 Report
PLAT188_ALERT_3_G A Non-default SIMU Restraint Value has been used     0.0100 Report
PLAT191_ALERT_3_G A Non-default SADI Restraint Value has been used     0.0010 Report
PLAT299_ALERT_4_G Atom Site Occupancy Constrained at ............         0.5 Check
             O6      N1      N2      N3      C1      C2      C4      C5
             C6      C7      C8      C9      C10     C11     C12     C13
             H2      H2A     H3      H5      H6A     H6B     H8      H9
             H11     H12     N9      C3
PLAT300_ALERT_4_G Atom Site Occupancy of Cl3        Constrained at     0.0625 Check
PLAT300_ALERT_4_G Atom Site Occupancy of O2         Constrained at     0.0625 Check
PLAT300_ALERT_4_G Atom Site Occupancy of O7         Constrained at     0.0625 Check
PLAT300_ALERT_4_G Atom Site Occupancy of O9         Constrained at     0.0625 Check
PLAT300_ALERT_4_G Atom Site Occupancy of O11        Constrained at     0.0625 Check
PLAT300_ALERT_4_G Atom Site Occupancy of O12        Constrained at       0.75 Check
PLAT300_ALERT_4_G Atom Site Occupancy of C14        Constrained at       0.75 Check
PLAT300_ALERT_4_G Atom Site Occupancy of H14        Constrained at       0.75 Check
PLAT300_ALERT_4_G Atom Site Occupancy of H3A        Constrained at       0.25 Check
PLAT300_ALERT_4_G Atom Site Occupancy of H3B        Constrained at       0.25 Check
PLAT300_ALERT_4_G Atom Site Occupancy of H3C        Constrained at       0.25 Check
PLAT300_ALERT_4_G Atom Site Occupancy of H9A        Constrained at       0.25 Check
PLAT300_ALERT_4_G Atom Site Occupancy of H9B        Constrained at       0.25 Check
PLAT300_ALERT_4_G Atom Site Occupancy of Cl1        Constrained at       0.25 Check
PLAT300_ALERT_4_G Atom Site Occupancy of O3         Constrained at       0.25 Check
```

```
PLAT300_ALERT_4_G Atom Site Occupancy of O5        Constrained at       0.25 Check
PLAT300_ALERT_4_G Atom Site Occupancy of O8        Constrained at       0.25 Check
PLAT300_ALERT_4_G Atom Site Occupancy of O10       Constrained at       0.25 Check
PLAT301_ALERT_3_G Main Residue  Disorder .............(Resd    1)        75% Note
PLAT302_ALERT_4_G Anion/Solvent/Minor-Residue Disorder (Resd    2)       100% Note
PLAT302_ALERT_4_G Anion/Solvent/Minor-Residue Disorder (Resd    3)       100% Note
PLAT302_ALERT_4_G Anion/Solvent/Minor-Residue Disorder (Resd    4)       100% Note
PLAT304_ALERT_4_G Non-Integer Number of Atoms in ..... (Resd    3)       5.50 Check
PLAT304_ALERT_4_G Non-Integer Number of Atoms in ..... (Resd    4)       1.25 Check
PLAT304_ALERT_4_G Non-Integer Number of Atoms in ..... (Resd    5)       0.12 Check
PLAT606_ALERT_4_G Solvent Accessible VOID(S) in  Crystal Structure          ! Info
PLAT779_ALERT_4_G Suspect or Irrelevant (Bond) Angle(s) in CIF ...      42.20 Deg.
              H3A  -C3   -H3C     1_555   1_555  21_575 ......     #  107 Check
PLAT779_ALERT_4_G Suspect or Irrelevant (Bond) Angle(s) in CIF ...      42.20 Deg.
              H3B  -C3   -H3B     1_555   1_555  21_575 ......     #  111 Check
PLAT779_ALERT_4_G Suspect or Irrelevant (Bond) Angle(s) in CIF ...      42.20 Deg.
              H3C  -C3   -H3A     1_555   1_555  21_575 ......     #  115 Check
PLAT780_ALERT_1_G Coordinates do not Form a Properly Connected Set     Please Do !
PLAT789_ALERT_4_G Atoms with Negative _atom_site_disorder_group  #         36 Check
PLAT811_ALERT_5_G No ADDSYM Analysis: Too Many Excluded Atoms ....          ! Info
PLAT822_ALERT_4_G CIF-embedded .res Contains Negative PART Numbers          5 Check
PLAT860_ALERT_3_G Number of Least-Squares Restraints ............         220 Note
PLAT868_ALERT_4_G ALERTS Due to the Use of _smtbx_masks Suppressed          ! Info
PLAT912_ALERT_4_G Missing # of FCF Reflections Above STh/L=  0.600         35 Note
PLAT951_ALERT_5_G Calculated (ThMax) and CIF-Reported Kmax Differer          3 Units
PLAT969_ALERT_5_G The 'Henn et al.' R-Factor-gap value ..........      8.129 Note
              Predicted wR2: Based on SigI**2  3.97 or SHELX Weight 29.62
```

---

   0 **ALERT level A** = Most likely a serious problem - resolve or explain
   1 **ALERT level B** = A potentially serious problem, consider carefully
  19 **ALERT level C** = Check. Ensure it is not caused by an omission or oversight
  62 **ALERT level G** = General information/check it is not something unexpected

```
  10 ALERT type 1 CIF construction/syntax error, inconsistent or missing data
  19 ALERT type 2 Indicator that the structure model may be wrong or deficient
  10 ALERT type 3 Indicator that the structure quality may be low
  39 ALERT type 4 Improvement, methodology, query or suggestion
   4 ALERT type 5 Informative message, check
```

---

---

It is advisable to attempt to resolve as many as possible of the alerts in all categories. Often the minor alerts point to easily fixed oversights, errors and omissions in your CIF or refinement strategy, so attention to these fine details can be worthwhile. In order to resolve some of the more serious problems it may be necessary to carry out additional measurements or structure refinements. However, the purpose of your study may justify the reported deviations and the more serious of these should normally be commented upon in the discussion or experimental section of a paper or in the "special_details" fields of the CIF. checkCIF was carefully designed to identify outliers and unusual parameters, but every test has its limitations and alerts that are not important in a particular case may appear. Conversely, the absence of alerts does not guarantee there are no aspects of the results needing attention. It is up to the individual to critically assess their own results and, if necessary, seek expert advice.

**Publication of your CIF in IUCr journals**

A basic structural check has been run on your CIF. These basic checks will be run on all CIFs submitted for publication in IUCr journals (*Acta Crystallographica*, *Journal of Applied Crystallography*, *Journal of Synchrotron Radiation*); however, if you intend to submit to *Acta Crystallographica Section C* or *E* or *IUCrData*, you should make sure that full publication checks are run on the final version of your CIF prior to submission.

**Publication of your CIF in other journals**

Please refer to the *Notes for Authors* of the relevant journal for any special instructions relating to CIF submission.

---

**PLATON version of 04/06/2025; check.def file version of 30/05/2025**

**Datablock xzz-240831-191928tetra_auto** - ellipsoid plot

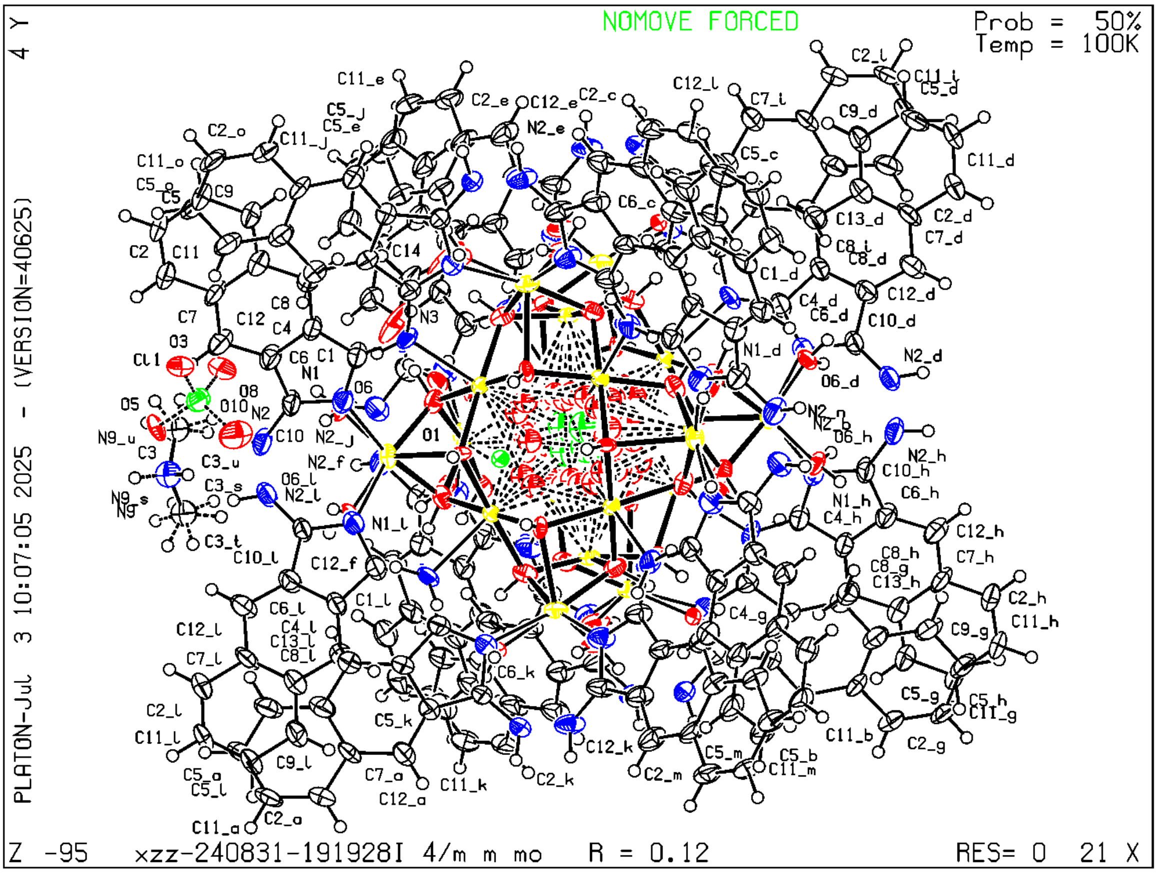

Supplement: Supplementary file 2 — Supporting cif files [file ADVS-13-e19498-s001.zip › Checkcif (compound 2).pdf]
